# Morphological Acclimation of Durum Wheat Spikes in Response to Foliar Micronutrient Applications

**DOI:** 10.3390/plants14193079

**Published:** 2025-10-05

**Authors:** Despina Dimitriadi, Georgios P. Stylianidis, Ioannis Tsirogiannis, Lampros D. Bouranis, Styliani N. Chorianopoulou, Dimitris L. Bouranis

**Affiliations:** 1 Plant Physiology and Morphology Laboratory, Crop Science Department, Agricultural University of Athens, Iera Odos 75, 11855 Athens, Greeceg.stylianidis@aua.gr (G.P.S.); tsiros02@yahoo.com (I.T.); s.chorianopoulou@aua.gr (S.N.C.); 2 Karvelas AVEE, 80th km Athinon-Lamias, 32200 Ypato, Greece; 3 Department of Statistics, Athens University of Economics and Business, 28is Oktovriou 76, 10434 Athens, Greece; bouranis@aueb.gr; 4 PlanTerra, Institute for Plant Nutrition and Soil Quality, Agricultural University of Athens, Iera Odos 75, 11855 Athens, Greece

**Keywords:** durum wheat spikes, spike morphological components, spike biofortification, spike acclimation, foliar biofortification additives

## Abstract

A cultivation of durum wheat that established in a field with soil poor in micronutrients received foliar applications at the initiation of the dough stage towards biofortifying the spikes with micronutrients. The morphology of the spike is crucial in determining grain yield, and the spikelets, the components of the inflorescence, influence each other. The number and arrangement of these spike components affect spike length, spike weight, spike chaff (the non-grain biomass in the spike), grain number per spike, grain weight per spike, and spikelet number per spike, and all contribute to final grain yield per spike. The spike’s developmental program responded to the interventions regarding the morphological traits; this response was analyzed for each spike component, and an acclimation program seemed to be activated by each intervention. Cysteine or methionine has been added as a potential enhancer of the biofortification process, and the application mixtures were coupled with selected surfactants, an organosilicon ethoxylate or an alcohol ethoxylate one, while products with targeted composition for biofortification with micronutrients have also been studied. Their effect on the developmental acclimation program of the treated spike is presented and discussed. The action of this program provided grains of similar weight, regardless of the intervention.

## 1. Introduction

Durum wheat, a tetraploid species, is the most resilient wheat type. It thrives in semiarid and hot, dry climates, outperforming bread wheat and other small grains in regions like the Mediterranean [1]. The term ‘durum’ derives from Latin, meaning ‘hard’, and this species is recognized as the hardest among all wheat varieties. This hardness specifically refers to the grain’s resistance to milling, particularly with respect to the starchy endosperm.

The morphology of the inflorescence, termed the spike, is crucial in determining the grain yield of the wheat plant. The spike (or head) is one of the most important parts of wheat [2]. It includes the reproductive organs, produces the seeds, and ensures dispersal of the mature grains. The spike has a rachis (stem) made up of nodes and short, flattened internodes.

At the nodes are the floral structures, the spikelets, which are the basic units of the inflorescence and contain florets, glumes, and lemmas. Spikelets can hold up to 10 florets containing the flower of the wheat plant, where grain is formed. The number and arrangement of spikelets affect spike length, spike weight, spike chaff (i.e., the non-grain biomass in the spike), grain number per spike, grain weight per spike, and spikelet number per spike, which all contribute to final grain yield per spike [3]. During the spike’s development, the spike’s components influence each other. Grain yield improvement in the last century was highly associated with an increase in grain number per unit area, which is largely determined by the grain number per spike [4]. Grain number per spike is determined by the combination of the number of spikelets per spike and the number of grains per spikelet. Each wheat spikelet has more than one grain. This makes the wheat spikelet one of the most essential grain yield components [5,6,7,8,9,10,11,12].

Moreover, awns are a characteristic feature of wheat spikes, being floral expansion. Awns are lemma-derived and photosynthetically active [13], with their known functions to include photosynthesis, carbohydrate storage, and increased water-use efficiency [14]. Awns contribute significantly to the photosynthetic output of the spike during the early phases of rapid grain growth, whilst during periods of water-deficit stress, the awns maintain a positive carbon exchange rate of the spike.

Spike morphology is a crucial phenotype for displaying the distribution of assimilates. The spike morphology-related traits demonstrate great variation under variable growth conditions. Therefore, environmentally induced plasticity of spike morphology-related traits can in principle affect the redistribution of assimilates. Source–sink relations is an important characteristic of the environmentally induced plasticity. It has been reported [15] that awns modify spike morphology by increasing sterile spikelet number and grain size, which implies that assimilates are redistributed within the spike due to the allocation of assimilates to large and rapidly developing awns.

Grain development is the period from flowering to physiological maturity when fertilized florets fill and ripen to form grain. The wheat grain has three growth stages: grain enlargement, grain fill, and physiological maturity [16]. During grain filling, grain weight increases at a constant rate as carbohydrate and protein are deposited into the grain. The grain moves from the milk stage through to the dough stage. Once the grain has reached the early dough stage, all the structural elements are laid down in the grain, and it is potentially viable. After this point the filling process continues loading carbohydrates and protein. At the soft dough stage, the grain starts to change color from green to yellow, maximum grain weight is achieved, and the grain has a moisture content of 40%. At hard dough, the grain is golden.

Biofortifying wheat grains with the essential micronutrients (EMi), zinc (Zn), iron (Fe), manganese (Mn), and copper (Cu) is a key strategy to reduce micronutrient deficiencies in human populations. Since agriculture-based foods are a primary source of nutrition, the link between soil nutrients, crops, and human health is clear [17]. Micronutrient deficiencies typically arise in regions where soils contain inadequate concentrations of plant-available micronutrients [18]. This objective is accomplished through foliar fertilization, among other strategies. Incorporating EMi-enriched fertilizers represents an effective strategy to address hunger and malnutrition. EMi fertilizers are crucial for wheat growth by improving nutrition, grain development, physiological traits, yield, and nutrient deficiencies, and they also benefit humans and animals [19,20]. Plants react to the external EMi deficiencies of the rhizosphere, and among other reactions, roots release Fe-chelators like phytosiderophores to mobilize Fe and Zn during nutrient deficiency [21]. The organic acids citrate and malate facilitate the transport of iron in the xylem, while nicotianamine (NA) facilitates Zn and Fe transport in the phloem and intracellularly [22]. In foliar applications, a wetter or surfactant (surface active agent) is usually incorporated. Surfactants are commonly incorporated into agrochemical formulations to enhance the biological efficiency of foliar sprays by improving the wetting behavior of the spray and/or the penetration of the active ingredients into the leaf tissues [23]. Penetration-accelerating surfactants are known to increase the cuticular permeability and may subject the cuticular barrier to water loss [24]. Among the various types of surfactants, two are in common use in the experimental area: alcohol ethoxylate (AE) surfactants or organosilicon-based ethoxylate (SiE) ones. Ethoxylated surfactants may improve spray retention and leaf wetting, while they may also increase cuticular permeability [25].

In this study, durum wheat was subjected to various interventions for two experimental years at the onset of the transition from milk to dough stage with the aim of biofortifying the grain with EMi (Fe, Cu, Mn, Zn) provided as sulfate salts, alone or coupled with cysteine (Cys) or methionine (Met), as precursors of NA, hence as potential biofortification enhancers. The mixtures were applied alone or in combination with commercial surfactants, namely SW7 (SiE type) or Saldo (AE type). The performance of two commercial products, FytoAmino-Bo (FABo) and Phillon, have been studied too. The study aimed at revealing any effects of these spray applications on spike morphological traits, if any. To our knowledge, there is no literature deepening on the effect of such EMi fertilizers applied foliarly for biofortification reasons on the spike’s developmental program and its potential reactions.

## 2. Results

### 2.1. Effects on Morphological Traits

The response of the spike population in each intervention has been presented by box plots where the reference range of each morphological trait has been highlighted to visualize the effect of the intervention on the population. The corresponding experimental material for both experimental years is provided in the Supplementary Materials Section.

**Figure 1:**
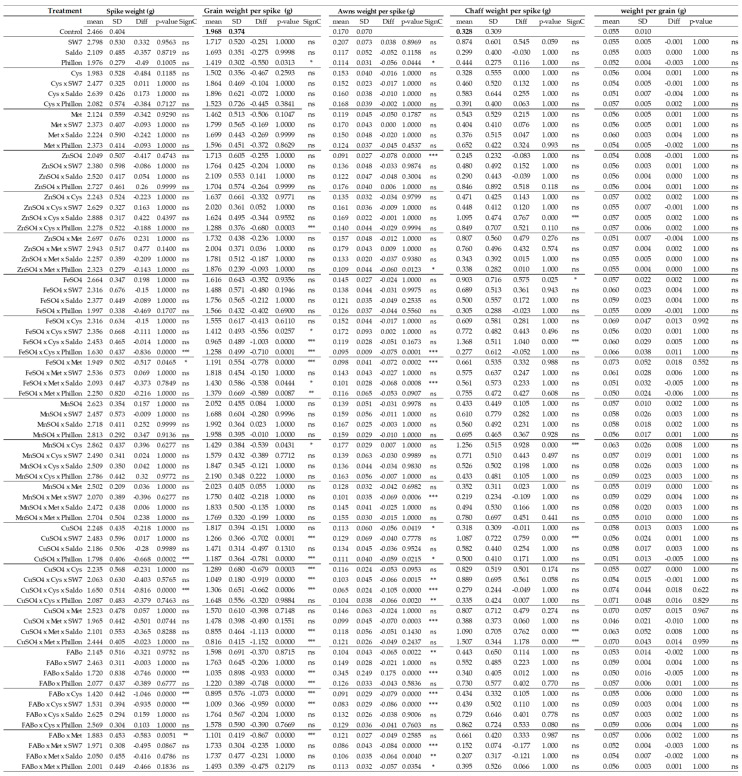
Spike weight, grain weight per spike, awns weight per spike, chaff weight per spike, and weight per grain as affected by the treatments (experimental year 2022–2023). Mean: mean values; SD: standard deviation; Diff: the difference between the mean value of the treatment and that of the control; SC: significance code; ns: not statistically significant; * *p* < 0.05; ** *p* < 0.01; *** *p* < 0.001.

Spike weight (SW)—The reference range for spike weight (SW) was found to be 2.062–2.870 g per spike with a mean value of 2.466 g (Figure 1 and Table S1), and the box plots for each treatment are depicted in Figure S1a,b.

Grain weight per spike (GWS)—The reference range was found to be 1.58–2.36 g (Figure 1 and Table S1) with a mean value of 1.97 g, and the box plots for each treatment are provided in Figure S2a,b. Twenty eight cases (out of 71) were found to present significantly lower grain weight per spike, while no case was found to be above the upper reference limit. This morphological trait is of significant agronomical importance.

Awns weight per spike (AWS)—The reference range for awn weight per spike was found to be 0.097–0.423 g per spike with a mean value of 0.170 g (Figure 1 and Table S1), and the box plots of all treatments are depicted in Figure S3a,b. Awns, in most cases, decreased their weight as a response to the interventions.

Chaff weight per spike (CWS)—The reference range for chaff weight per spike was found to be 0.637–0.0019 g per spike with a mean value of 0.328 g (Figure 1 and Table S1), and the box plots of all treatments are depicted in Figure S4a,b. In most cases, chaff weight was increased as a response to the interventions.

Weigh per grain (GW)—The reference range was found to be 0.065–0.045 g (Figure 1 and Table S1) with a mean value of 0.55 g, and the box plots for each treatment are provided in Figure S5a,b. All cases presented not statistically significant changes; however, there were tendencies to increasing GW. This finding implies that after the intervention, the reaction mechanism included strong support of the existing grains within the reference range (or more).

**Figure 2:**
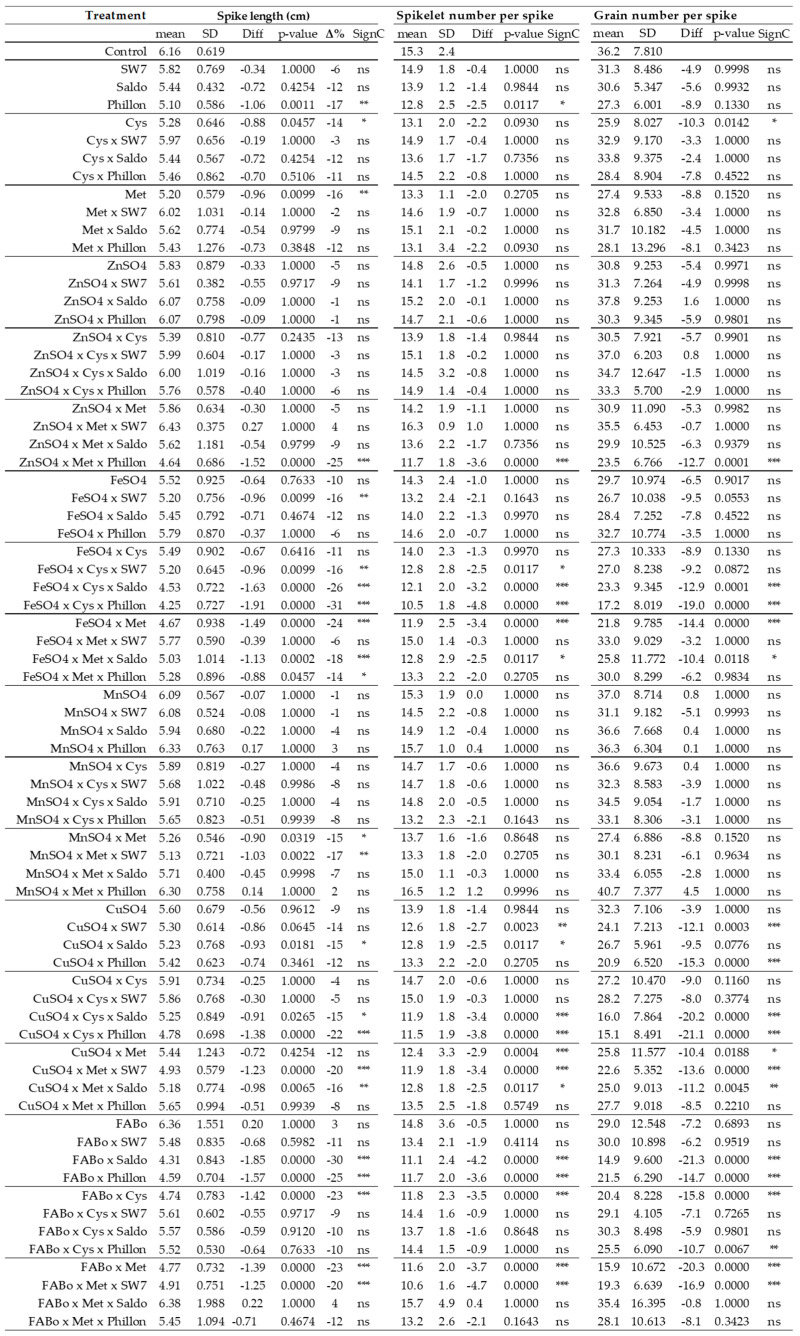
Spike length, spikelet number per spike, and grain number per spike as affected by the treatments (experimental year 2022–2023). Mean: mean values; SD: standard deviation; Diff: the difference between the mean value of the treatment and that of the control; SC: significance code; ns: not statistically significant; * *p* < 0.05; ** *p* < 0.01; *** *p* < 0.001.

Spike length (SL)—The reference SL was 6.2 cm, and the reference range was between 5.6 and 6.8 cm (Figure 2 and Figure S6a,b and Table S2). There were 24 interventions (out of 71) that presented statistically significant reductions in mean SL (Δ% < −15%), while 19/71 presented not statistically significant tendencies to decrease mean SL (−15% < Δ% < −10%). ZnSO_4_ treatments presented a milder reduction in SL from 3% to −26%; ZnSO_4_ itself by −6%. All treatments with FeSO_4_ reduced SL from −6% to −31%; FeSO_4_ itself by −11%. CuSO_4_ reduced SL from −5% to −23%; MnSO_4_ presented two tendencies to increase SL (both by 2%) and then reductions in SL of up to −23%; MnSO_4_ itself by −2%. CuSO_4_ itself by −10%. A combination of FABo presented a statistically significant increase (by 15%), and FABo itself by 3%; other than that, reductions in SL were observed up to −31%.

Spikelet number per spike (SNS)—The observed reference range for spikelet number per spikelet was 13–17, with a mean of 15 (Figure 2 and Table S2). Box plots illustrating the distribution of populations across each treatment are presented in Figure S7a,b. Seventeen cases were found to present significantly fewer spikelet numbers per spike, while four cases (out of 71) were in the upper limit of the reference range.

Grain number per spike (GNS)—The reference range for grain number per spike was 28–44 (Figure 2 and Table S2) with a mean value of 36. The corresponding box plots are provided in Figure S8a,b. Several cases presented significantly lower grain number per spike, while no case was found with a number above the upper reference limit.

**Figure 3:**
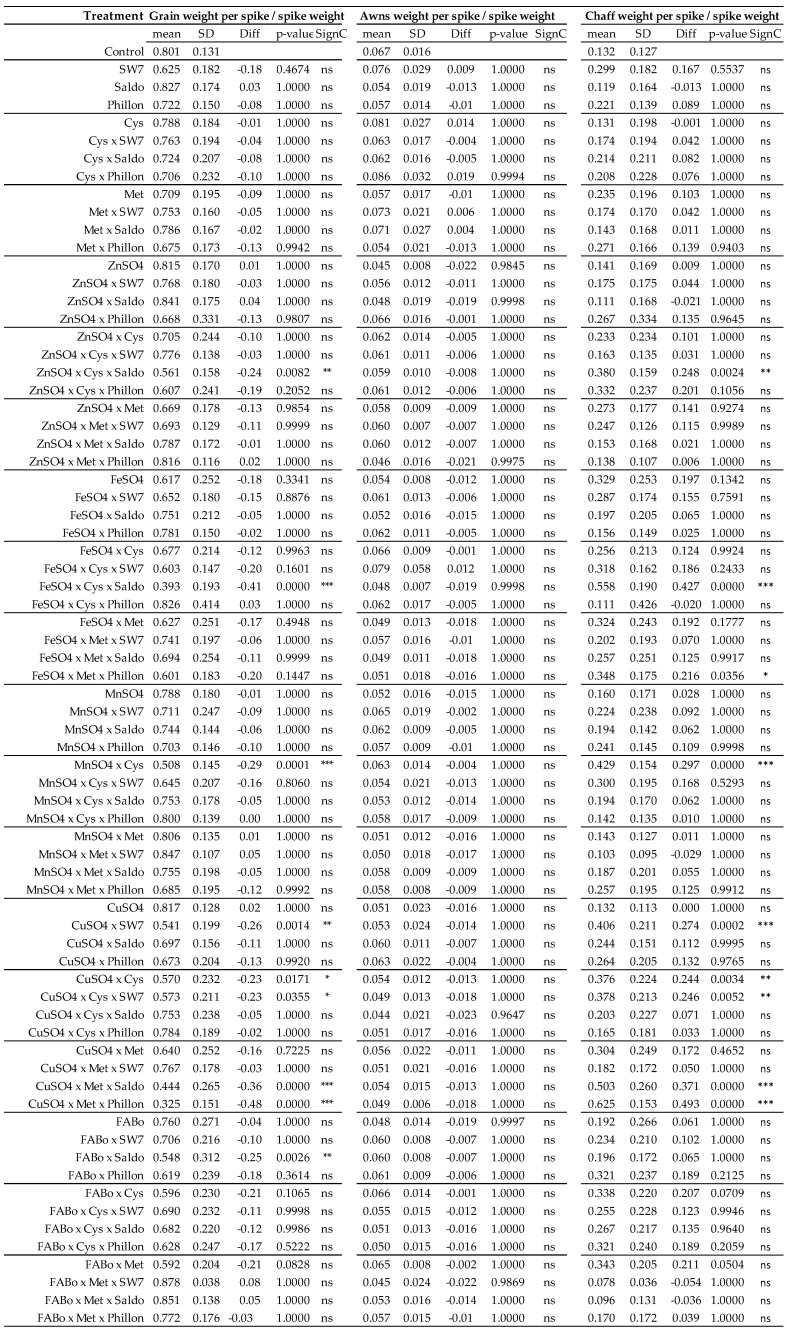
Grain weight per spike/spike weight, awns weight per spike/spike weight, chaff weight per spike/spike weight as affected by the treatments (experimental year 2022–2023). Mean: mean values; SD: standard deviation; Diff: the difference between the mean value of the treatment and that of the control; SC: significance code; ns: not statistically significant; * *p* < 0.05; ** *p* < 0.01; *** *p* < 0.001.

**Figure 4:**
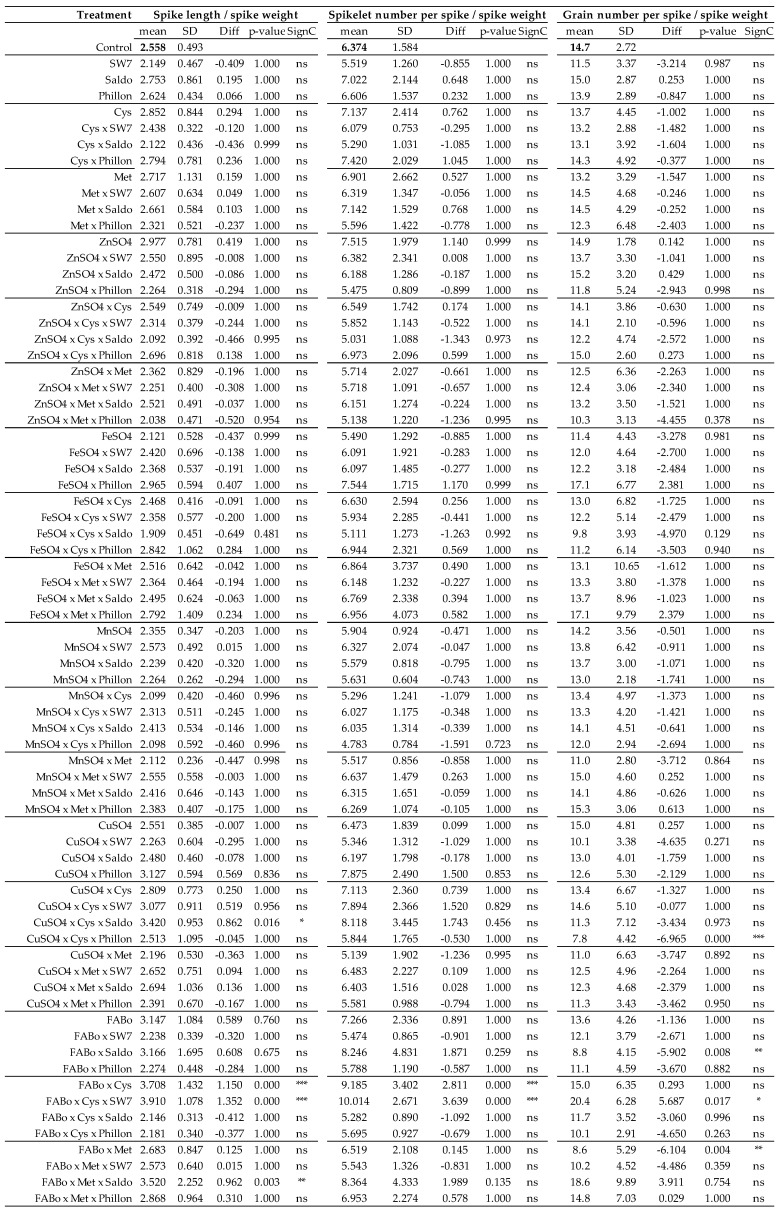
Spike length/spike weight, spikelet number per spike/spike weight, grain number per spike/spike weight as affected by the treatments (experimental year 2022–2023). Mean: mean values; SD: standard deviation; Diff: the difference between the mean value of the treatment and that of the control; SC: significance code; ns: not statistically significant; * *p* < 0.05; ** *p* < 0.01; *** *p* < 0.001.

These results have also been expressed in per unit of spike weight, including the following ratios: GWS/SW (Figure 3, Figure S9a,b and Table S3), AWS/SW (Figure 3, Figure S10a,b and Table S3), CWS/SW (Figure 3, Figure S11a,b and Table S3), SL/SW (Figure 4, Figure S12a,b and Table S4), SlNS/SW (Figure 4, Figure S13a,b and Table S4), and GNS/SW (Figure 4, Figure S14a,b and Table S4).

### 2.2. Correlations Between Morphological Traits

Findings indicated that the morphological traits of the spike examined were influenced by the foliar interventions. To further investigate these findings, we conducted correlation analysis of the morphological parameters (Pearson’s test; Table 1 and Table S5).

**Figure 5:**
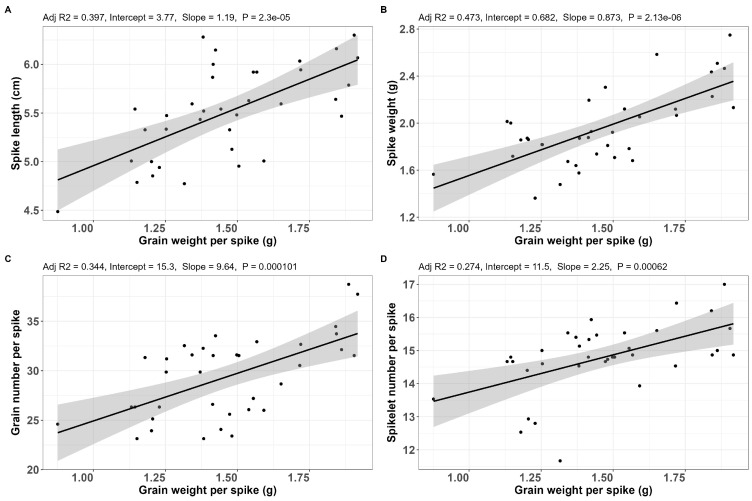
Correlations between GWS and SL (**A**), SW (**B**), GNS (**C**), and SlNS (**D**) (experimental year 2022–2023, Figure S1 in the Supplementary Materials).

Grain weight per spike vs. spike length—The correlation showed (Figure 5A) that there was a linear relationship between the spike shortening and the corresponding grain weight per spike. A good proportion of the points is lower than the min reference values. A shorter spike length indicates a decrease in grain weight per spike, which is the most important agronomic trait.

Grain weight per spike vs. spike weight—This relationship proved to be less significant compared to that of GWS vs. SL (Figure 5B). The min reference value for spike weight was 1.8 g.

Grain weight per spike vs. grain number per spike—This relationship proved to be a very strong linear one (Figure 5C). The min reference value for grain number per spike was 28 grains. Again, a good proportion of the points is out of the reference range, towards the reduction side, which indicates that reduction in grain weight per spike reflects reduction in grain number per spike, which is also a trait of agronomical significance.

Grain weight per spike vs. spikelet number per spike—Another strong linear relationship, with the same profile as the previous ones (Figure 5D). The reference range for spikelet number per spike was 13–17 spikelets. Reduction in grain weight per spike reflects reduction in spikelet number per spike.

Similar results have been received for the experimental year 2021–2022 (Figure S1 see Supplementary Materials).

### 2.3. Comparison of the Effects on Morphological Traits

**Figure 6:**
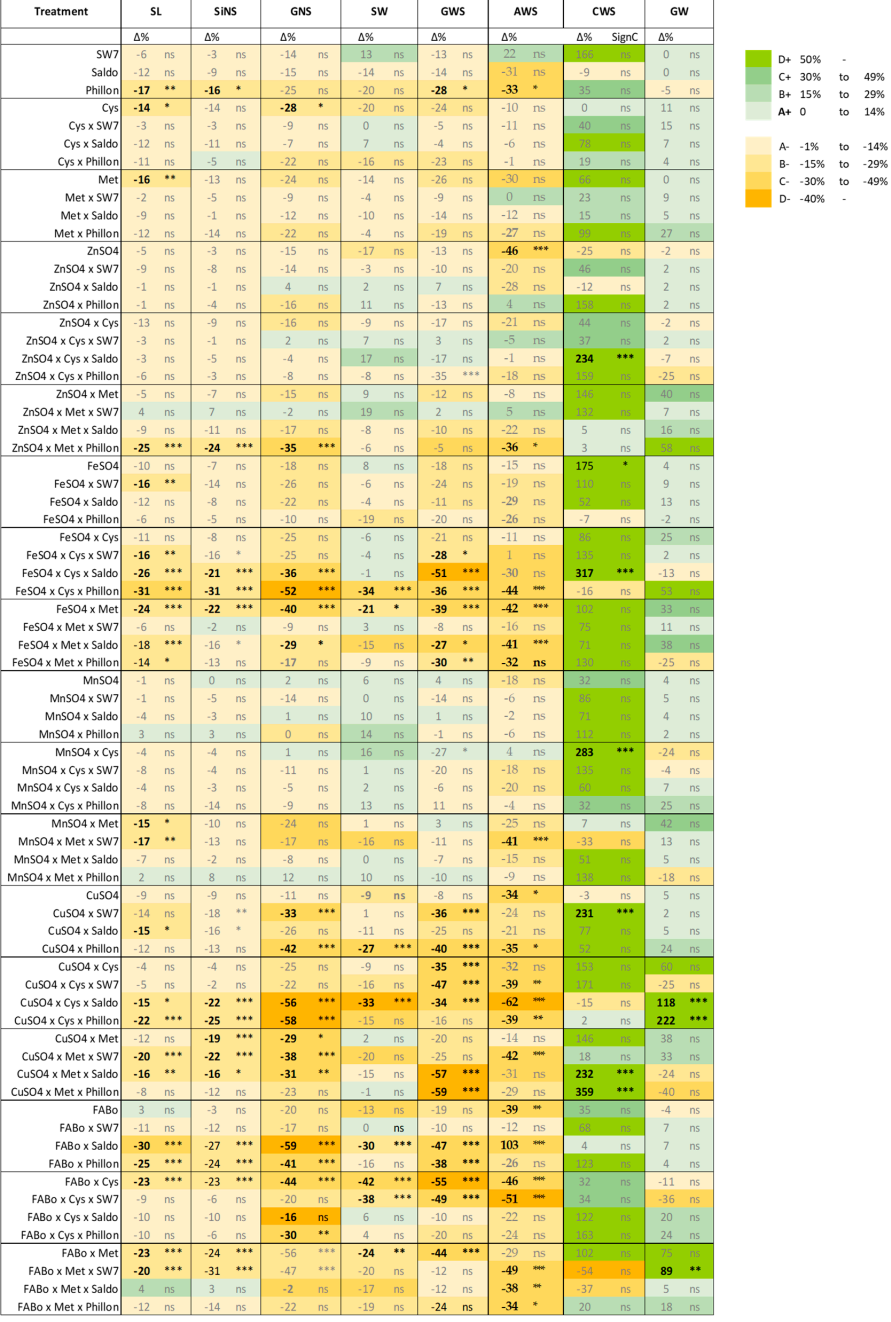
The percentage change in each trait’s mean value is relative to the reference treatment (control; Δ%), along with the statistical significance. SL: spike length, SlNS: spikelet number per spike, GNS: grain number per spike, SW: spike weight, AWS: awns weight per spike, CWS: chaff weight per spike, GWS: grain weight per spike, GW: grain weight. A scale has been adopted to visualize also the tendencies for increasing or decreasing when Δ% is not statistically significant. ns: not statistically significant; *: *p*-value < 0.05; **: *p*-value < 0.01; ***: *p*-value < 0.001. The *p*-values are provided in the corresponding Tables.

**Figure 7:**
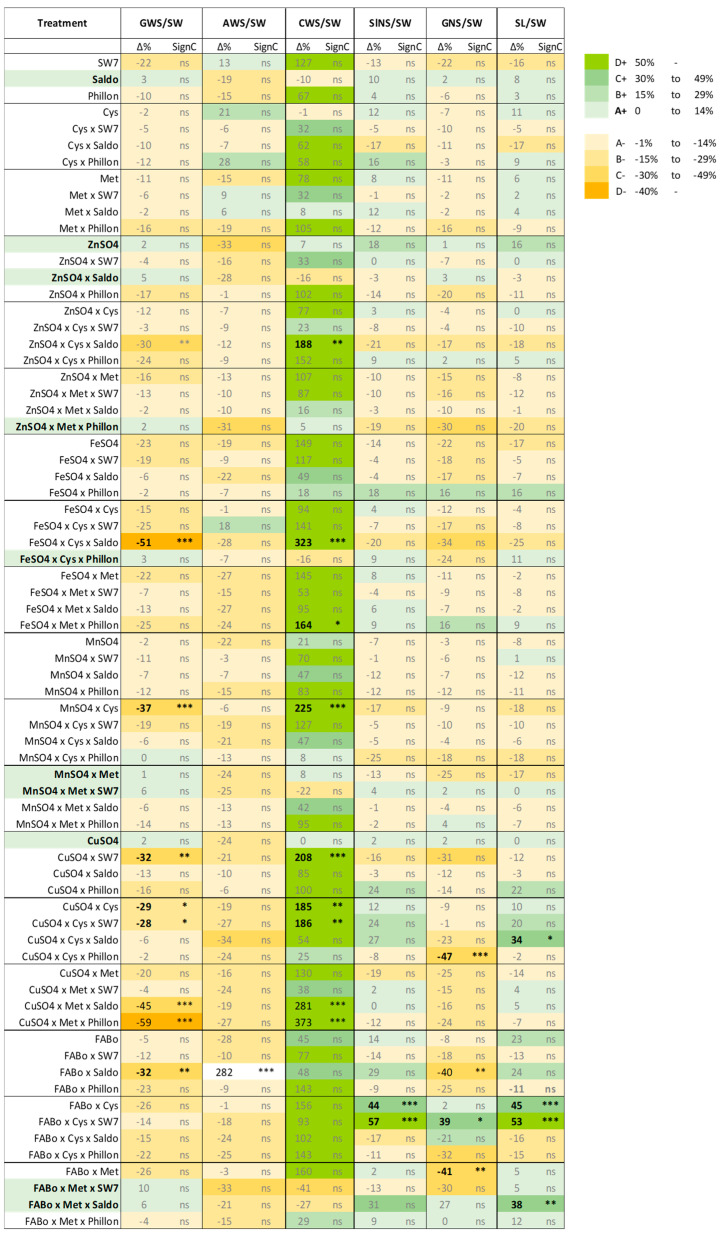
The percentage change in each trait’s mean value (MV) relative to the reference treatment (control; Δ%); along with the statistical significance. GWS/SW: grain weight per spike/spike weight; AWS/SW: awns weight per spike/spike weight; CWS/SW: chaff weight per spike/spike weight; SlNS/SW: spikelet number per spike/spike weight; GNS/SW: grain number per spike/spike weight; SL/SW: spike length/spike weight/spike weight. A scale has been adobted to visualize also the tendencies for increasing or decreasing; when Δ% is not statistically significant. ns: not statistically significant; * *p*-value < 0.05; ** *p*-value < 0.01; *** *p*-value < 0.001. The *p*-values are provided in the corresponding Tables.

The effect of the interventions on each morphological trait was differentiated between them, revealing an acclimation program of the treated spike in action (TSAP), and the broad picture is provided in Figure 6 and Figure 7.

One of the observations is that the intervention affected SL by reducing it or attempting to reduce it. On the other hand, the weight of each grain (GW) remained stable. Reduction was also observed in the awns. In contrast, apart from decreases in SW, there are also increases in some treatments. The same holds true for SlNS. We hypothesize that after the intervention, the upper part of the spike ceases developing and the existing grains receive priority in resource allocation within spike components.

**Figure 8:**
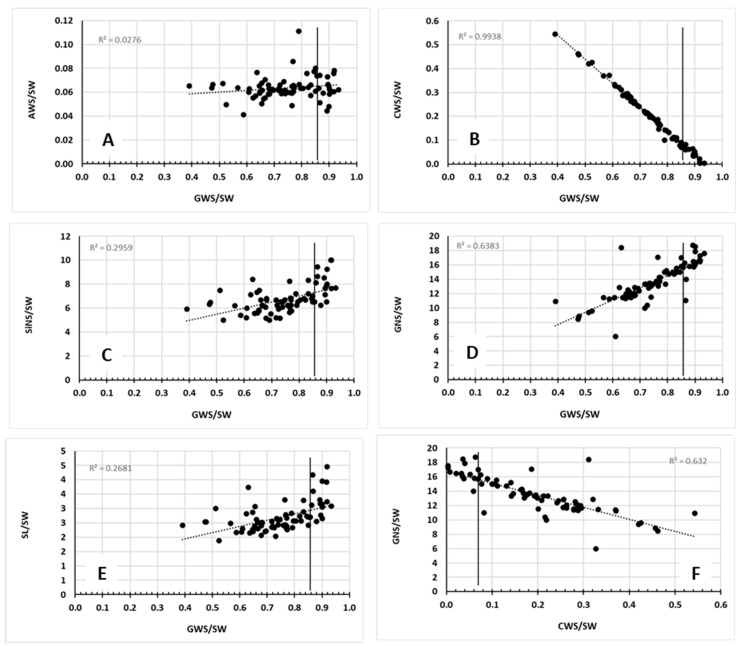
Relationships between GWS/SW and AWS/SW (**A**), WS/SW (**B**), SlNS/SW (**C**), GNS/SW (**D**), and SL/SW (**E**), along with CWS/SW vs. GNS/SW (**F**). Line indicates the corresponding value of control treatment.

The economic aspect of the TSAP is highlighted by the GW per kg of spike ratio (GWS/SW), which shows that there were some increases as shown by Δ%. The ratios AWS/SW, CWS/SW, SlNS/SW, and GNS/SW provide the percentages of the carbon allocation between the various components of the spike, i.e., grains, awns, and chaff, along with the maximum numbers of spikelets and grains per spike, which are flexible within this allocation program. Figure 8 corroborates the fact that when GW increases, this causes a linear decrease in CW and vice versa. Similar correlations have been received for the experimental year 2021–2022 (Figure S2).

## 3. Discussion

This study shows that the spray application of the treatments affected the spike’s developmental program, which switched to the acclimation process. The described alterations suggest that the signaling is affected by the treatments. The applications took place at the beginning of the dough stage. Final grain yield is predominantly a function of grain number hosted in the spikelets. The terminal spikelet signals the end of the spike development phase. After the terminal spikelet is initiated, rapid spike growth begins, and the spike stem elongates. The alterations observed in spike architecture may stem from disruptions in hormonal equilibrium induced by foliar sprays. Gradients and signaling pathways of auxin, cytokinins, and gibberellins are critical for coordinating meristem activity and the development of yield-related organs [26,27,28]. Foliar micronutrient application can influence hormonal balance by modifying the activity of enzymes involved in hormone biosynthesis: for instance, zinc-dependent auxin synthesis and iron-dependent synthesis of gibberellins and cytokinins. Notably, previous studies have demonstrated that foliar fertilization can alter endogenous hormone levels in both wheat and grapevine [29,30].

On the other hand, if we focus on the micronutrients’ treatments that resulted in statistically significant changes in all measured parameters (except grain weight), i.e., SL, SiNS, GNS, SW, AWS, and GWS, we will have the list of those treatments that had the greatest effect on spike architecture. In this line, combining Figure 6 and Figure 7, we see that those treatments included Fe and Cu. This fact highlights another potential player in shaping the architecture of the spike under the conditions studied: reactive oxygen species (ROS). Iron and Cu are redox-active metals that can switch between two oxidation states and are known to have a pro-oxidant role and be directly involved in ROS generation. On the contrary, Zn and Mn mostly have roles in ROS scavenging processes. Moreover, the fact that in various treatments with Fe and Cu, Cys is also present in the formulation points out its role as a superior ligand for both metals [31,32,33]. Reactive oxygen species may have several roles in wheat spike growth and architecture, i.e., as morphogenic signals regulating meristem activity and floral organ development or as players in hormonal crosstalk through their interactions with key hormones in spike development.

Agronomic biofortification involves applying EMi-containing mineral fertilizers to soil or plant foliage to boost EMi levels in edible crop parts [22,34]. The agronomic biofortification activity to increase the EMi content is associated with an increase in yield and quality. The sulfate salts Fe, Cu, Zn, and Mn have been employed to address deficiencies of EMi in seeds and edible tissues, as compared to the use of EMi complexed with synthetic ligands. Hence, in this work, EMis have been provided as sulfate salts. It has been reported [35] the available information describes the connections between the homeostasis of S and Fe, Cu, Zn, and Mn in plants. The roles of S- or S-derived organic ligands in metal uptake and translocation within the plant are highlighted. Moreover, the roles of these micronutrients in S homeostasis have been discussed [22].

S-containing compounds are provided through foliar application, including the S metabolites cysteine and methionine, among others. Free EM can be toxic; therefore, EMs in plants exist as free ions in either very small amounts or not at all. Instead, the metals that are present in plant fluids must be in less reactive chemical forms, bound to “proper” organic compounds, to prevent the uncontrolled binding. Selected organic molecules are implicated in metal ion binding and are known as metal ion ligands or chelators. Chelation improves acquisition and transport of EM with low solubility, along with immobilization, toward EM storage and tolerance. The formation of EM complexes provides solubility as well as protection during long-distance transport, as the EM atom is surrounded by the ligands. These chemical species donate a number of electron pairs to the EM to form the complexes. Possible candidates as ligands are several small molecules, the amino acids Cys and Met among them. Nicotianamine has been shown to participate in the transport of the EMi Fe, Cu, Mn, and Zn. NA is produced by methionine and acts as a chelator through its carboxyl groups, possessing a special role in the interaction between S and EMi homeostasis. NA is synthesized by NA synthase (NAS) from S-adenosyl-L-methionine. It is a ubiquitous metal chelator in all plants. NA is an Fe chelator, and it has demonstrated its ability to bind both Fe and Cu. It is believed to play a primary role in EMi homeostasis, and therefore it links EMi homeostasis to S homeostasis [22].

Several mechanisms are involved in phloem unloading and post-phloem movement of EMi in the developing seed. These include the movement through apoplastic barriers. The loading rates of EMi imported through the phloem are regulated by translocation processes localized in both the sources, i.e., the leaves and the stems, and the seed sinks [35]. Phloem unloading in the developing seed seems to be symplastic into a specific domain, with symplastic connections to the entire seed coat. Active transport is required for EMi to exit from living cells, as membranes present highly negative potential on the inside. Yield and seed quality are related to the S content of the seed. S presents a great impact on the improvement of seed yield and quality. The S is transported from leaves, as the major source, during the various developmental phases of seed. Remobilization of S takes place in mature leaves and/or stems, which are S transient storage pools. The information on the source-to-sink relationship during the time of seed development is rather poor. The same holds true for the role of sulfate exported from the seed vacuoles during the seed developmental stage in order to maintain cellular homeostasis. SULTR3 and SULTR4 family transporters seem to be involved in S transportation mechanisms during seed development [35]. As regards the form of S delivered to seeds through the phloem, this is diverse; in wheat, S is delivered as S-methylmethionine. Transportation of sulfate through sulfate transporters localized in the phloem contributes to the import of S in seeds. The S-reducing enzymes are also present in the developing seeds, resulting in the accumulation of reduced sulfur in mature seeds [36,37,38,39,40].

The thiol group reacts with H_2_O_2_ (oxidation of thiol), NO (S-nitrosation), and H_2_S (persulfidation) [41]. H_2_S contributes to the physiological processes in action [42]. Perhaps H_2_S produced by sulfate in the spike contributes to the physiological processes in action at the time following foliar application; H_2_S contributes to signal network [43]. The role of H_2_S in plant resistance to abiotic stress, including drought and heat, by regulating miRNAs [44]. A model of thiol oxidation, persulfidation, and S-nitrosylation modifications has been presented [45].

In agricultural applications, S-containing compounds are typically used in combination with other substances in spray solutions, making the selection of appropriate combinations essential [34]. Alcohol ethoxylates are used as surfactants in a wide variety of agrochemical formulations to enhance the effectiveness of the active constituents. Alcohol ethoxylates belong to the class of compounds that are synthesized via the reaction of a fatty alcohol and ethylene oxide, resulting in a molecule that consists of two parts: one a carbon-rich, fatty alcohol and the second part a hydrophilic polyoxyethylene chain [18]. Saldo contains isodecyl alcohol ethoxylate, while Phillon contains ZnSO_4_, coupled with another AE, and humic acids. As regards SiE, the silicon–oxygen bonds are hydrophobic, whilst the ethoxylated clusters are hydrophilic, creating a wetting agent that spreads quickly, thus covering a large surface area, greater than conventional surfactants. Silicone surfactants undergo a relatively rapid hydrolytic cleavage in the environment, as do linear silicone polymers, to give monomers that are more slowly converted by oxidation back to water, CO_2_, and sand [6,7]. Generally, surfactant effects are species- and compound-specific [46].

Taken together, the applied treatments are founded on the previously mentioned S-based combinations, and the results indicate that additional combinations with selected common surfactants have yielded compound-specific effects on the phenotypical traits of the spike.

## 4. Materials and Methods

Two field experiments were carried out in 2021–2022 and 2022–2023 at the area of the Experimental Fields of the Agricultural University of Athens, at the location of Aliartos, Viotia, Greece (coordinates: 38°23′48.3″ N, 23°05′23.8″ E). The soil was found to present the following characteristics: clay 42%, calcium carbonate 50.4%, organic matter 2.8%, pH 8.4, K: 110 ppm, P: 9.2 ppm, exchangeable Mg 528 ppm, Fe 5.1 ppm, Mn 3.7 ppm, Zn 0.72 ppm, Cu 0.32 ppm, and B 0.4 ppm.

**Figure 9:**
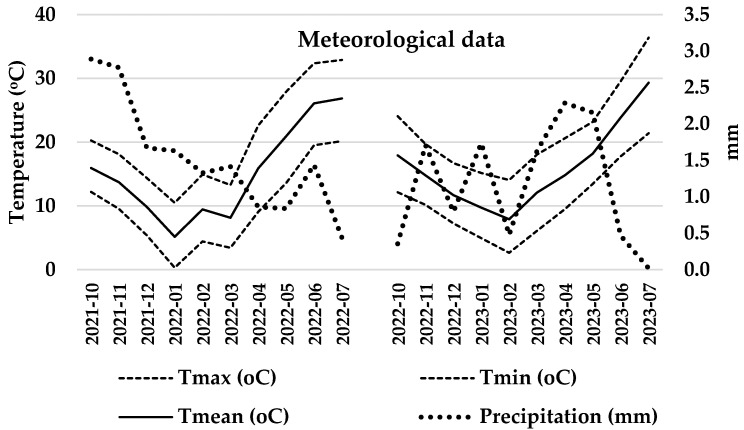
Meteorological data.

The durum wheat variety studied was Don Matteo. The agronomic program included the following: basic fertilization, NOVATEC 20-20-5 + B + Zn (Compo Expert Hellas, Athens, Greece); herbicide application, Corello 75wg (Corteva Agriscience, Athens, Greece) and Mustang (DOW Agriscience, Athens, Greece); fungicide application, Madison (Bayer Cropscience, Athens, Greece); top dressing fertilization, OMEGA 26-0-0 (Hellagrolip, Kavala, Greece) were applied, while no irrigation was applied to the crop. The dates of the experiment’s agronomic works are provided in Table 2. The experimental plots were formed with dimensions of 3 m × 1.5 m and 1 m distance between them. The interventions followed with foliar application at the developmental stage Z80, following a randomized block design. The volume of spray liquid was 1 L per experimental plot, while the sprays took place during the afternoon-early evening hours (18:00–21:30). During the harvest, 10 heads were sampled from the main stem per experimental plot. Per treatment, three plots were studied, and in total, 30 spikes were analyzed per treatment. The meteorological data are depicted in Figure 9.

For each spike, the initial weight was measured and recorded, and then the awns were removed. All morphological traits were measured manually and recorded after the removal of awns.

The treatments are provided in Table 3. The results of the first year, where SW7 was used as a surfactant, encouraged us to try two more and different surfactants, those of Saldo and Phillon. The interventions included EMi as sulfates, alone or in combination with cysteine (CJ CheilJedang, Seoul, Republic of Korea) or methionine (CJ CheilJedang, Seoul, Republic of Korea). Thus, applications of copper sulfate (0.31 mM), ferrous sulfate (8.93 mM), zinc sulfate (0.31 mM), and manganese sulfate (3.64 mM) were carried out in combination with or without the addition of methionine (5 mM) or cysteine (5 mM). The Fytoamino-Bo (prepared by D. Dimitriadi at Karvelas AVEE, Greece) product’s composition was as follows: 2% *w*/*w* Zn sulfate, 5% ethanolamine borate, 1% sodium molybdate, and 5% L-arginine. The water used was a drinkable, commercial one with composition presented in Table 4.

The surfactants used were the following: SW7 (Omex Ltd, Norfolk, Great Britain; distributor: KARVELAS AVEE, Greece): It is an ethoxylated organosilicate formulation containing 6.5% *w*/*v* silicon (Si), and the applied dosage was 1 mL of wetting agent per L of application solution (0.1% *v*/*v*). Salso (Saldo Plus 15SL, SEGE SA, Athens Greece): It contained ethoxylated isodecyl alcohol 15% *w*/*v*, and the applied dosage was 0.3 mL of wetting agent per L of application solution (0.03% *v*/*v*). Phillon (prepared by D. Dimitriadi at KARVELAS AVEE, Greece). This product contains in its composition soybean oil and 1.2% *w*/*w* zinc as zinc sulfate. The wetting agent incorporated in the formulation was Lutensol T08 (BASF SA, Athens, Greece), a non-ionic wetting agent and emulsifier made from a saturated iso-C13 alcohol containing 8 moles of ethylene oxide. The applied dosage was 1 mL of product per L of application solution (0.1% *v*/*v*).

### Statistical Analysis

The morphological trait measurements were handled as continuous variables. Analysis of variance (ANOVA) was used to explore the effects of various treatments on the trait of interest. Due to multiple testing, Tukey’s ‘Honest Significant Difference’ method [47] was used to control the 95% family-wise confidence level. Significance tests were declared significant when *p*-value < 0.05. All analyses were performed using the R statistical software (Version 4.0.0) [48].

## 5. Conclusions

Following the foliar treatments, the spike activated an acclimation program. All treatments demonstrated either a tendency to decrease SW or resulted in a statistically significant reduction. This outcome indicates that modification of spike morphological traits is one of the responses to the applied solutions at the initiation of the dough phase. The target of this program was to maintain or even increase the weight of the existing grains. The development does not follow the rate of the reference treatment and ceases source allocation to the upper spikelets and grains; hence, the final spike is shorter than the reference one. Awns become shorter, and chaff weight is regulated accordingly. Grain weight per spike emerged as the primary determinant, exhibiting linear associations with spike length, grain number per spike, and spikelet number per spike; however, no such strong relationship was observed with spike weight. Despite these modifications, the weight per grain consistently remained within the 50–59 mg range, indicating that the spike maintained stable grain mass. Furthermore, the data indicated that combinations of the micronutrients Fe, Cu, Zn, or Mn in their sulfate forms can be applied without the need for a surfactant. Similarly, the combinations of ZnSO_4_ with either Cys or Met do not require the addition of a surfactant. In all other instances, trisiloxane SW7 was determined to be the optimal choice for ZnSO_4_ or FABo combinations, whereas the evaluated alcohol ethoxylate surfactants were found to be effective with FeSO_4_, CuSO_4_, or MnSO_4_ applications.

## Figures and Tables

**Table 1:** Pearson’s correlation test providing Pearson’s linear correlation coefficient, along with the estimated confidence interval, and the *p*-value for the hypothesis that the coefficient is a statistical ratio of zero. LB: lower bound; UB: upper bound.

Trait 1	Trait 2	Coefficient	*p*-Value	LB	UB
AWS	GNS	0.22	5.930 × 10^−2^	−0.009	0.433
AWS	GWS	0.19	1.140 × 10^−1^	−0.046	0.402
AWS	SL	0.16	1.730 × 10^−1^	−0.072	0.380
AWS	SlNS	0.22	6.620 × 10^−2^	−0.015	0.428
AWS	SW	0.42	2.130 × 10^−4^	0.212	0.596
GNS	GWS	0.63	2.710 × 10^−9^	0.468	0.753
GNS	SL	0.84	3.370 × 10^−20^	0.754	0.897
GNS	SlNS	0.88	1.060 × 10^−24^	0.819	0.925
GNS	SW	0.68	3.910 × 10^−11^	0.536	0.790
GWS	SL	0.53	1.640 × 10^−6^	0.341	0.679
GWS	SlNS	0.49	9.940 × 10^−6^	0.297	0.652
GWS	SW	0.61	1.380 × 10^−8^	0.439	0.737
SL	SlNS	0.92	4.840 × 10^−31^	0.882	0.952
SL	SW	0.59	5.560 × 10^−8^	0.413	0.721
SlNS	SW	0.57	1.310 × 10^−7^	0.396	0.712

**Table 2:** Dates of agronomic works.

Dates	Agronomic Works
**Experimental 2021–2022**	
22 December 2021	Sowing
7 March 2022	Formation of the experimental plots
6 May 2022	Application of interventions
8 June 2022	Harvest day
**Experimental 2022–2023**	
4 January 2023	Sowing
24 April 2023	Formation of the experimental plots
28 June 2023	Harvest day
30 June 2023	Harvest day

**Table 3:** Treatments per experimental year.

**Experimental 2021–2022**			
Reference	SW7		
Cys	Cys,SW7		
Met	Met,SW7		
ZnSO_4_	ZnSO_4_,SW7		
ZnSO_4_,Cys	ZnSO_4_,Cys,SW7		
ZnSO_4_,Met	ZnSO_4_,Met,SW7		
FeSO_4_	FeSO_4_,SW7		
FeSO_4_,Cys	FeSO_4_,Cys,SW7		
FeSO_4_,Met	FeSO_4_,Met,SW7		
MnSO_4_	MnSO_4_,SW7		
MnSO_4_,Cys	MnSO_4_,Cys,SW7		
MnSO_4_,Met	MnSO_4_,Met,SW7		
CuSO_4_	CuSO_4_,SW7		
CuSO_4_,Cys	CuSO_4_,Cys,SW7		
CuSO_4_,Met	CuSO_4_,Met,SW7		
FABo	FABo,SW7		
FABo,Cys	FABo,Cys,SW7		
FABo,Met	FABo,Met,SW7		
**Experimental 2022–** **2023**			
Reference	SW7	Saldo	Phillon
Cys	Cys,SW7	Cys,Saldo	Cys,Phillon
Met	Met,SW7	Met,Saldo	Met,Phillon
ZnSO_4_	ZnSO_4_,SW7	ZnSO_4_,Saldo	ZnSO_4_,Phillon
ZnSO_4_,Cys	ZnSO_4_,Cys,SW7	ZnSO_4_,Cys,Saldo	ZnSO_4_,Cys,Phillon
ZnSO_4_,Met	ZnSO_4_,Met,SW7	ZnSO_4_,Met,Saldo	ZnSO_4_,Met,Phillon
FeSO_4_	FeSO_4_,SW7	FeSO_4_,Saldo	FeSO_4_,Phillon
FeSO_4_,Cys	FeSO_4_,Cys,SW7	FeSO_4_,Cys,Saldo	FeSO_4_,Cys,Phillon
FeSO_4_,Met	FeSO_4_,Met,SW7	FeSO_4_,Met,Saldo	FeSO_4_,Met,Phillon
MnSO_4_	MnSO_4_,SW7	MnSO_4_,Saldo	MnSO_4_,Phillon
MnSO_4_,Cys	MnSO_4_,Cys,SW7	MnSO_4_,Cys,Saldo	MnSO_4_,Cys,Phillon
MnSO_4_,Met	MnSO_4_,Met,SW7	MnSO_4_,Met,Saldo	MnSO_4_,Met,Phillon
CuSO_4_	CuSO_4_,SW7	CuSO_4_,Saldo	CuSO_4_,Phillon
CuSO_4_,Cys	CuSO_4_,Cys,SW7	CuSO_4_,Cys,Saldo	CuSO_4_,Cys,Phillon
CuSO_4_,Met	CuSO_4_,Met,SW7	CuSO_4_,Met,Saldo	CuSO_4_,Met,Phillon
FABo	FABo,SW7	FABo,Saldo	FABo,Phillon
FABo,Cys	FABo,Cys,SW7	FABo,Cys,Saldo	FABo,Cys,Phillon
FABo,Met	FABo,Met,SW7	FABo,Met,Saldo	FABo,Met,Phillon

**Table 4:** Spray water characteristics: Marata (Sklavenitis). Source: “Hitos” AVEE, Kranoula Ioanninon, Greece.

pH	7.75	Ca^2+^	79.90 mg L^−1^	Cl^−^	5.65 mg L^−1^
Electric conductivity (20 °C)	359 μS cm^−1^	Mg^2+^	4.34 mg L^−1^	SO_4_^2−^	17.00 mg L^−1^
Hardness (as CaCO_3_)	217.45 mg L^−1^	Na^+^	4.61 mg L^−1^	NO_3_^−^	15.15 mg L^−1^
		K^+^	0.89 mg L^−1^	NO_2_^−^	-
		NH_4_^+^	-	CO_3_^2−^	-

## Data Availability

The original contributions presented in this study are included in the article/Supplementary Materials. Further inquiries can be directed to the corresponding author.

## Supplementary Materials

The following supporting information can be downloaded at: https://www.mdpi.com/article/10.3390/plants14193079/s1, Figures and Tables regarding the experimental year 2021–2022 are provided as Supplementary Material. Figure S1. Correlations between GWS and SL (A), SW (B), GNS (C), and SlNS (D) (experimental year 2021–2022). Figure S1a. Box plot diagram for spike weight per treatment (experimental year 2022–2023). Figure S1b. Box plot diagram for spike weight per treatment (experimental year 2021–2022). Figure S2a. Box plot diagram for grain weight per spike, for each treatment (experimental year 2022–2023). Figure S2b. Box plot diagram for grain weight per spike, for each treatment (experimental year 2021–2022). Figure S3a. Box plot diagram for awns weight per spike, for each treatment (experimental year 2022–2023). Figure S3b. Box plot diagram for awns weight per spike, for each treatment (experimental year 2021–2022). Figure S4a. Box plot diagram for chaff weight per spike, for each treatment (experimental year 2022–2023). Figure S4b. Box plot diagram for chaff weight per spike, for each treatment (experimental year 2021–2022). Figure S5a. Box plot diagram for weight per grain, for each treatment (experimental year 2022–2023). Figure S5b. Box plot diagram for weight per grain, for each treatment (experimental year 2021–2022). Figure S6a. Box plot diagram for spike length, for each treatment (experimental year 2022–2023). Figure S6b. Box plot diagram for spike length, for each treatment (experimental year 2021–2022). Figure S7a. Box plot diagram for spikelet number per spike, for each treatment (experimental year 2022–2023). Figure S7b. Box plot diagram for spikelet number per spike, for each treatment (experimental year 2021–2022). Figure S8a. Box plot diagram for grain number per spike, for each treatment (experimental year 2022–2023). Figure S8b. Box plot diagram for grain number per spike, for each treatment (experimental year 2021–2022). Figure S9a. Box plot diagram for grain weight per spike/spike weight ratio, for each treatment (experimental year 2022–2023). Figure S9b. Box plot diagram for grain weight per spike/spike weight ratio, for each treatment (experimental year 2021–2022). Figure S10a. Box plot diagram for awns weight per spike/spike length ratio, for each treatment (experimental year 2022–2023). Figure S10b. Box plot diagram for awns weight per spike/spike length ratio, for each treatment (experimental year 2021-2022). Figure S11a. Box plot diagram for chaff weight per spike/spike weight ratio, for each treatment (experimental year 2022–2023). Figure S11b. Box plot diagram for chaff weight per spike/spike weight ratio, for each treatment (experimental year 2021–2022). Figure S12a. Box plot diagram for spike length/spike weight ratio, for each treatment (experimental year 2022–2023). Figure S12b. Box plot diagram for spike length/spike weight ratio, for each treatment (experimental year 2021–2022). Figure S13a. Box plot diagram for spikelet number per spike/spike weight ratio, for each treatment (experimental year 2022–2023). Figure S13b. Box plot diagram for spikelet number per spike/spike weight ratio, for each treatment (experimental year 2021–2022). Figure S14a. Box plot diagram for grain number per spike/spike weight ratio, for each treatment (experimental year 2022–2023). Figure S14b. Box plot diagram for grain number per spike/spike weight ratio, for each treatment (experimental year 2021–2022). Figure S15. Relationships between GWS/SW and AWS/SW (A), WS/SW (B), SlNS/SW (C), GNS/SW (D), and SL/SW (E), along with CWS/SW vs. GNS/SW (F). Line indicates the corresponding value of control treatment. Table S1. Spike weight, grain weight per spike, awns weight per spike chaff weight per spike, weight per grain as affected by the treatments (experimental year 2021–2022). Mean: mean values; SD: standard deviation, Diff: the difference between the mean value of the treatment and that of the control, SC: significance code; ns: not statistically significant, * *p* < 0.05, ** *p* < 0.01, *** *p* < 0.001. Table S2. Spike length, spikelet number per spike, and grain number per spike as affected by the treatments (experimental year 2021–2022). Mean: mean values; SD: standard deviation, Diff: the difference between the mean value of the treatment and that of the control, SC: significance code; ns: not statistically significant, * *p* < 0.05, ** *p* < 0.01, *** *p* < 0.001. Table S3. Grain weight per spike/spike weight, awns weight per spike/spike weight, chaff weight per spike/spike weight as affected by the treatments (experimental year 2021–2022). Mean: mean values; SD: standard deviation, Diff: the difference between the mean value of the treatment and that of the control, SC: significance code; ns: not statistically significant, * *p* < 0.05, ** *p* < 0.01, *** *p* < 0.001. Table S4. Spike length/spike weight, spikelet number per spike/spike weight, grain number per spike/spike weight as affected by the treatments (experimental year 2021–2022). Mean: mean values; SD: standard deviation, Diff: the difference between the mean value of the treatment and that of the control, SC: significance code; ns: not statistically significant, * *p* < 0.05, ** *p* < 0.01, *** *p* < 0.001. Table S5. Pearson’s Correlation Test providing Pearson’s linear correlation coefficient, along with the estimated confidence interval, and the *p*-value for the hypothesis that the coefficient is a statistical ratio of zero. LB: lower bound, UB: upper bound. Table S6. The percentage change in each trait’s mean value relative to the reference treatment (control; Δ%), along with the statistical significance (experimental year 2021–2022). SC: significance code, SL: spike length, SlNS: spikelet number per spike, GNS: grain number per spike, SW: spike weight, AWS: awns weight per spike, CWS: chaff weight per spike, GWS: grain weight per spike, GW: grain weight. A scale has been adobted to visualize the tendencies for increasing or decreasing, when Δ% is not statistically significant. ns: not statistically significant, *: *p*-value < 0.05, **: *p*-value < 0.01, ***: *p*-value < 0.001. The *p*-values are provided in the corresponding Tables. Table S7. The percentage change in each trait’s mean value (MV) relative to the reference treatment (control; Δ%), along with the statistical significance (experimental year 2021-2022). SC: significance code GWS/SW: grain weight per spike/spike weight, AWS/SW: awns weight per spike/spike weight, CWS/SW: chaff weight per spike/spike weight, SlNS/SW: spikelet number per spike/spike weight, GNS/SW: grain number per spike/spike weight, SL/SW: spike length/spike weight/spike weight. A scale has been adobted to visualize the tendencies for increasing or decreasing, when Δ% is not statistically significant. ns: not statistically significant, *: *p*-value < 0.05, **: *p*-value < 0.01, ***: *p*-value < 0.001. The *p*-values are provided in the corresponding Tables.

## Abbreviations

The following abbreviations are used in this manuscript:

Cys	Cysteine
Met	Methionine
EMi	Essential micronutrients
AE	Alcohol ethoxylate
SiE	Organosilicone-based ethoxylate
FABo	FytoAmino-Bo (product)
NA	Nicotianamine
SL	Spike length
SlNS	Spikelet number per spike
GNS	Grain number per spike
SW	Spike weight
GWS	Grain weight per spike
AWS	Awns weight per spike
CWS	Chaff weight per spike
GW	Grain weight (or weight per grain)
GWS/SW	Grain weight per spike/spike weight
AWS/SW	Awns weight per spike/spike weight
CWS/SW	Chaff weight per spike/spike weight
SlNS/SW	Spikelet number per spike/spike weight
GNS/SW	Grain number per spike/spike weight
SL/SW	Spike length/spike weight
