# Supplementary material for: Morphological Acclimation of Durum Wheat Spikes in Response to Foliar Micronutrient Applications
Source: Plants (Basel). 2025 Oct 5;14(19):3079. doi: 10.3390/plants14193079 (PMC12526200; doi:10.3390/plants14193079)
Supplement: Supplementary file 1 [file plants-14-03079-s001.zip › plants-3807460-supplementary.pdf]

Article

Morphological acclimation of durum wheat spike in response to foliar micronutrient applications

Despina Dimitriadi[1,2], Georgios P. Stylianidis[1], Ioannis Tsirogiannis[1], Lampros D. Bouranis[3], Styliani N. Chorianopoulou[1,4], Dimitris L. Bouranis[1,4,*]

Supplementary material

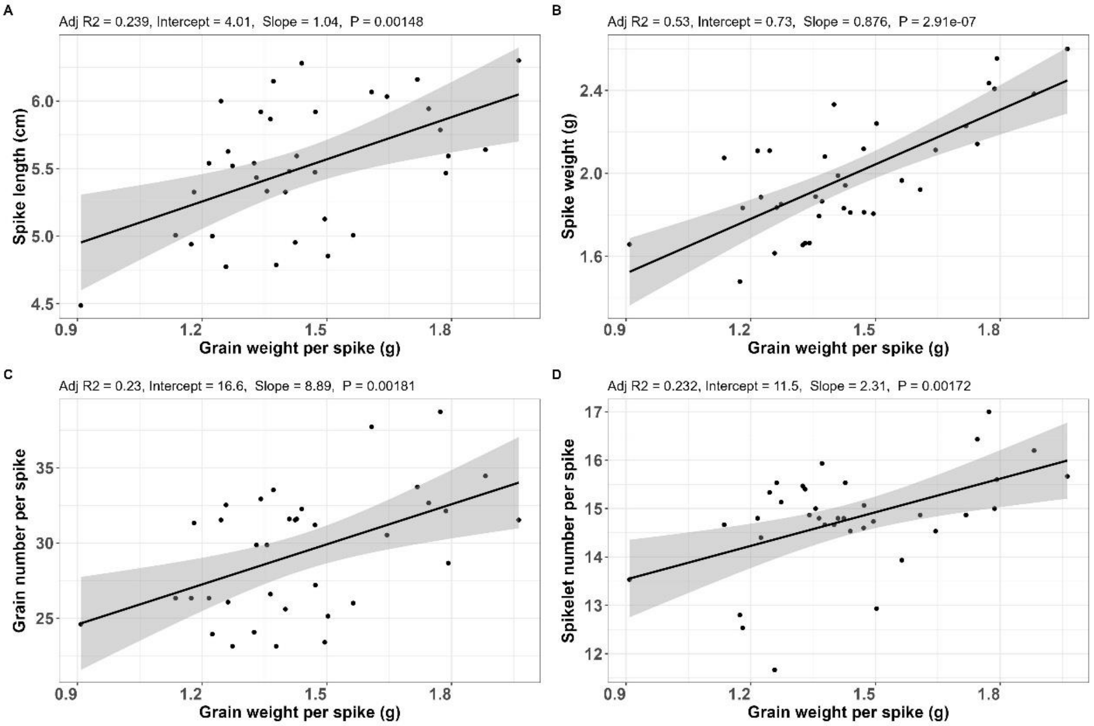

Figure S1. Correlations between GWS and SL (A), SW (B), GNS (C), and SlNS (D) (experimental year 2021-2022).

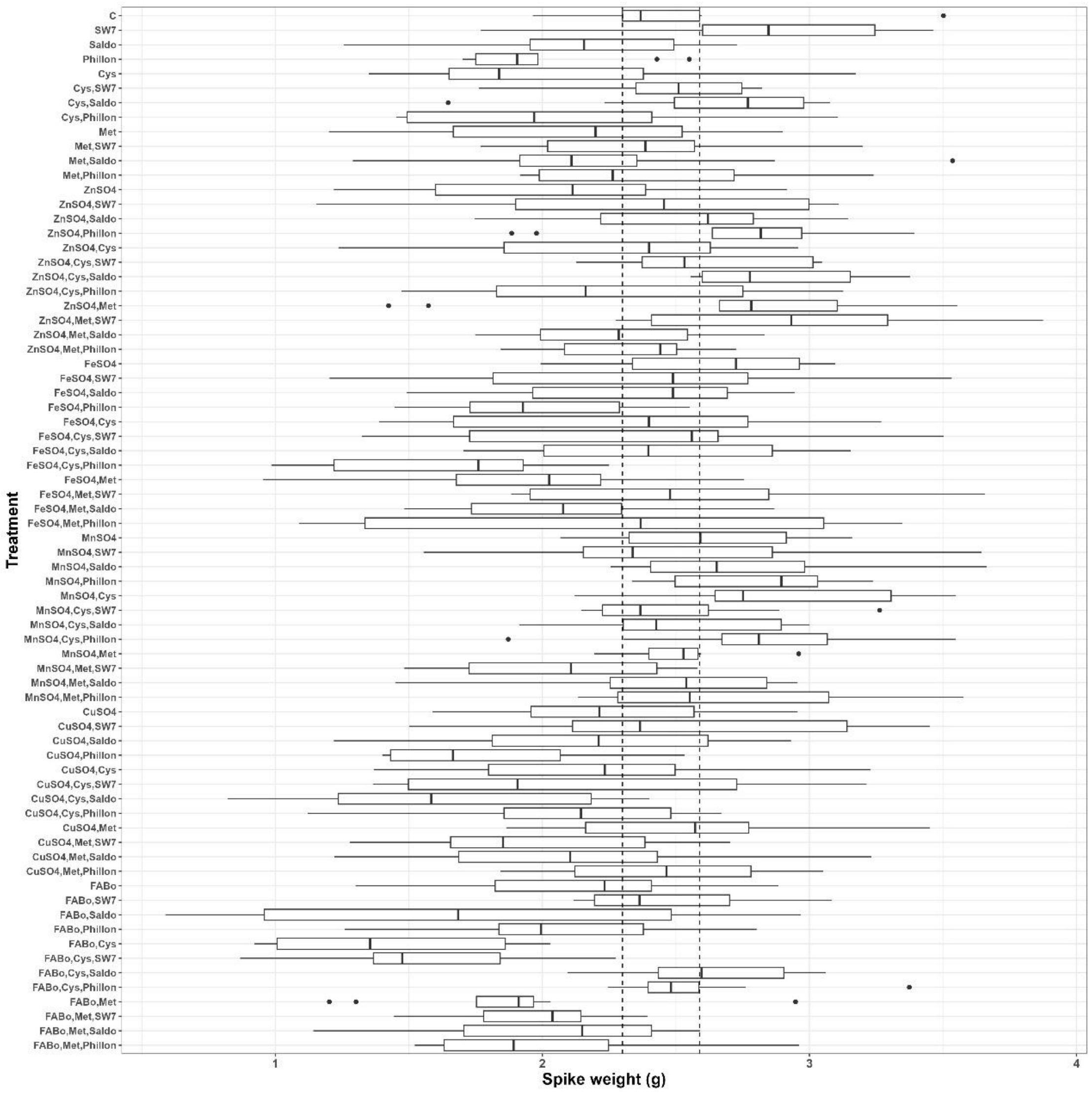

Figure S1a. Box plot diagram for spike weight per treatment (experimental year 2022-2023).

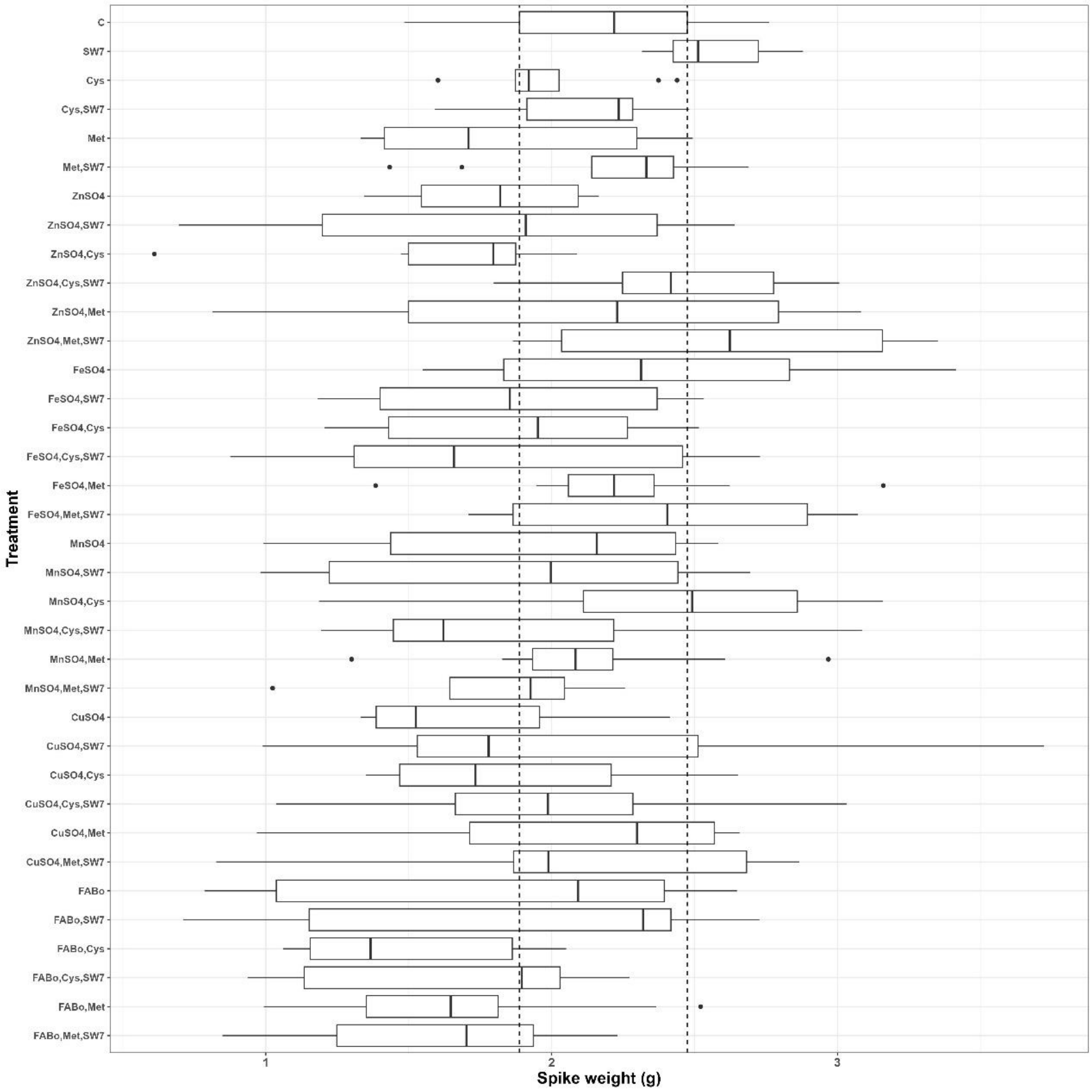

Figure S1b. Box plot diagram for spike weight per treatment (experimental year 2021-2022).

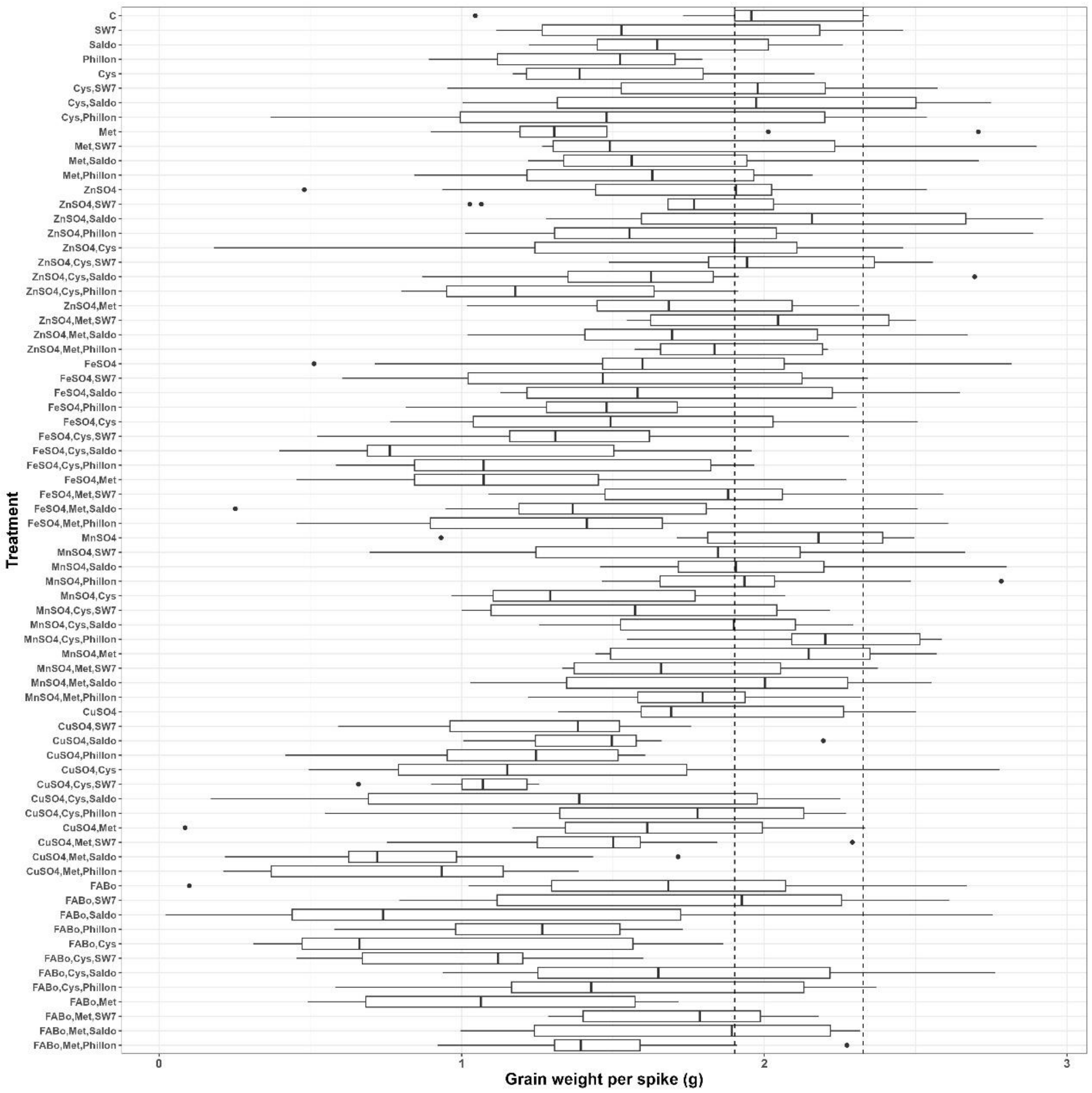

Figure S2a. Box plot diagram for grain weight per spike, for each treatment (experimental year 2022-2023).

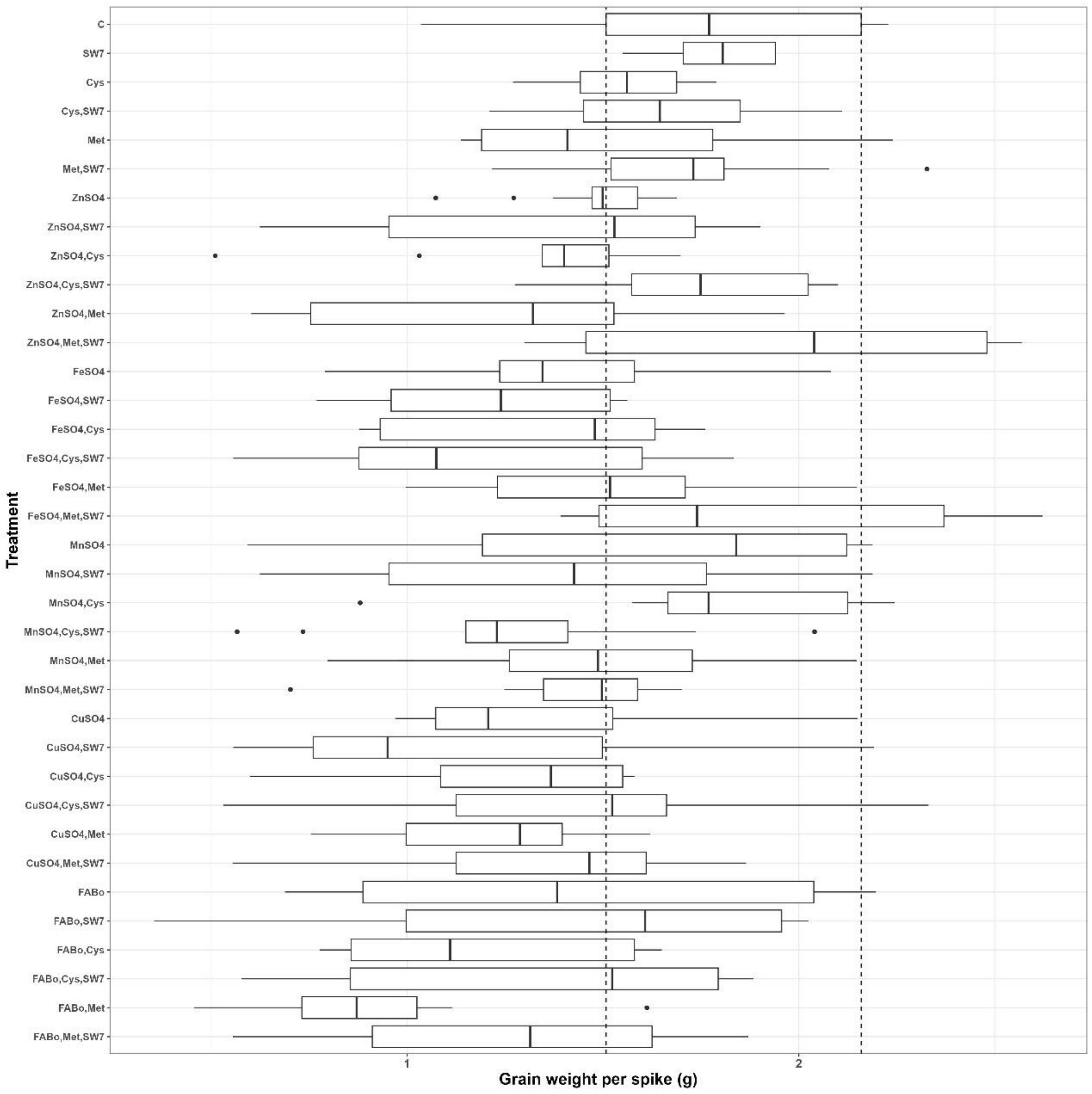

Figure S2b. Box plot diagram for grain weight per spike, for each treatment (experimental year 2021-2022).

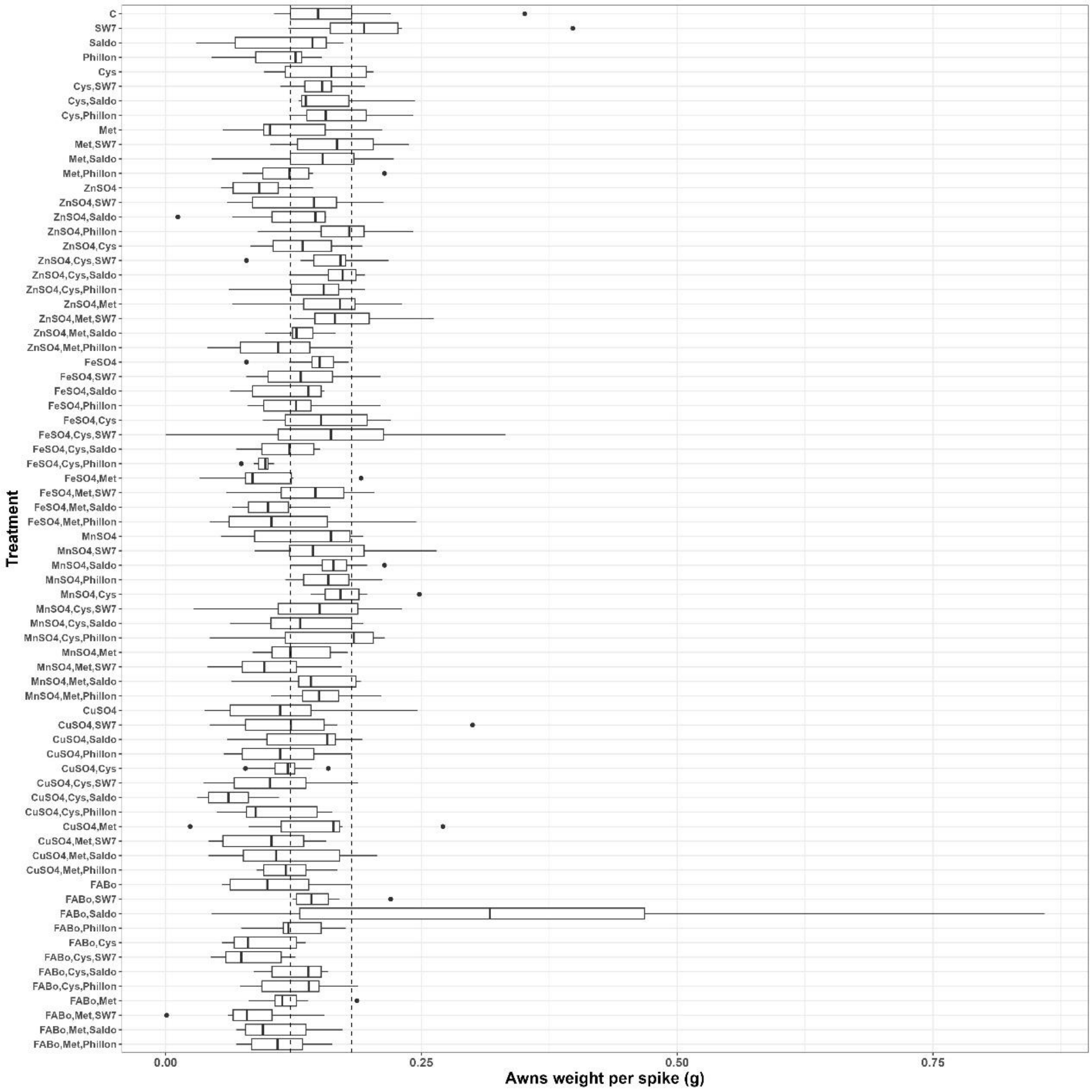

Figure S3a. Box plot diagram for awns weight per spike, for each treatment (experimental year 2022-2023).

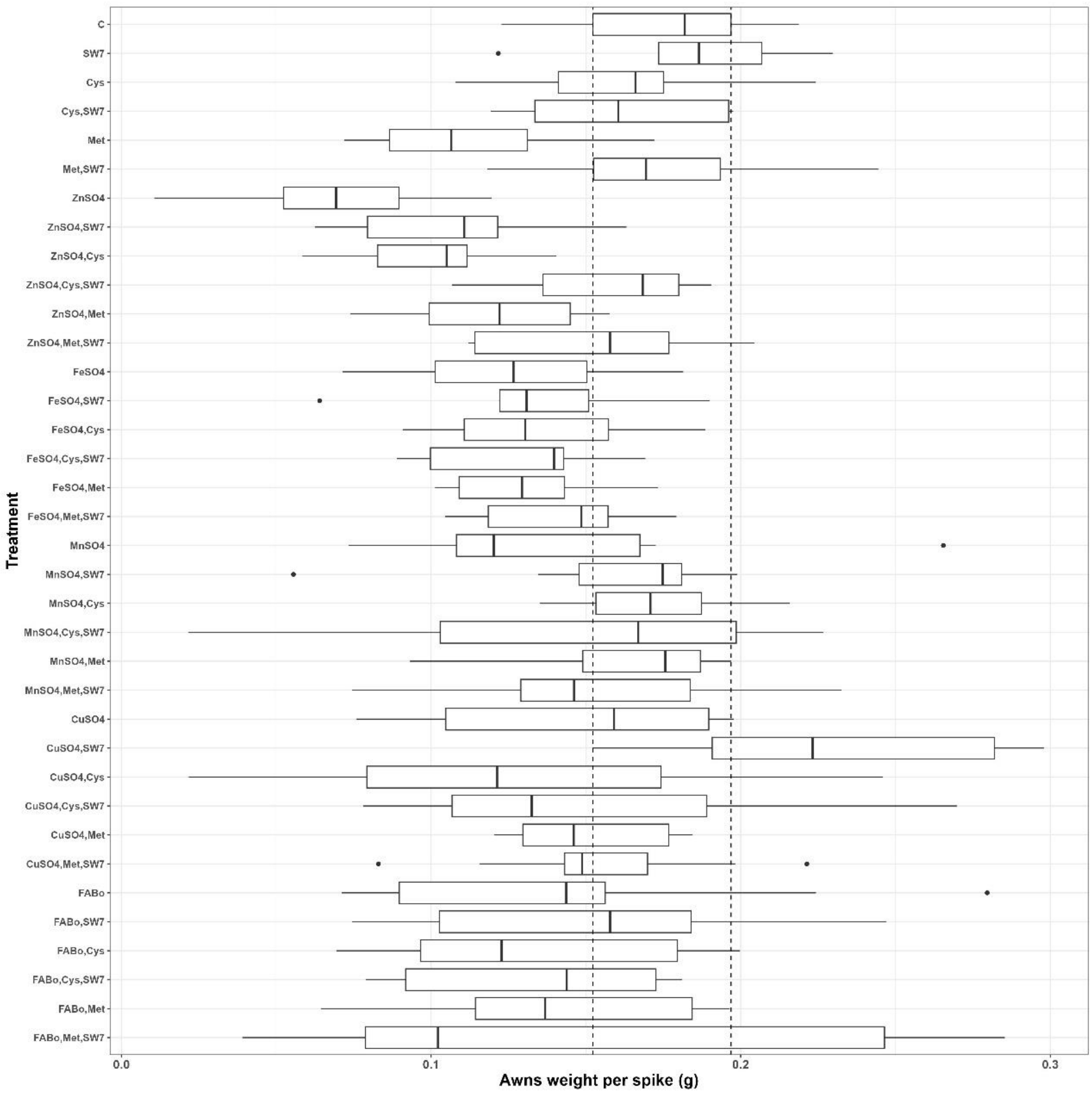

Figure S3b. Box plot diagram for awns weight per spike, for each treatment (experimental year 2021-2022).

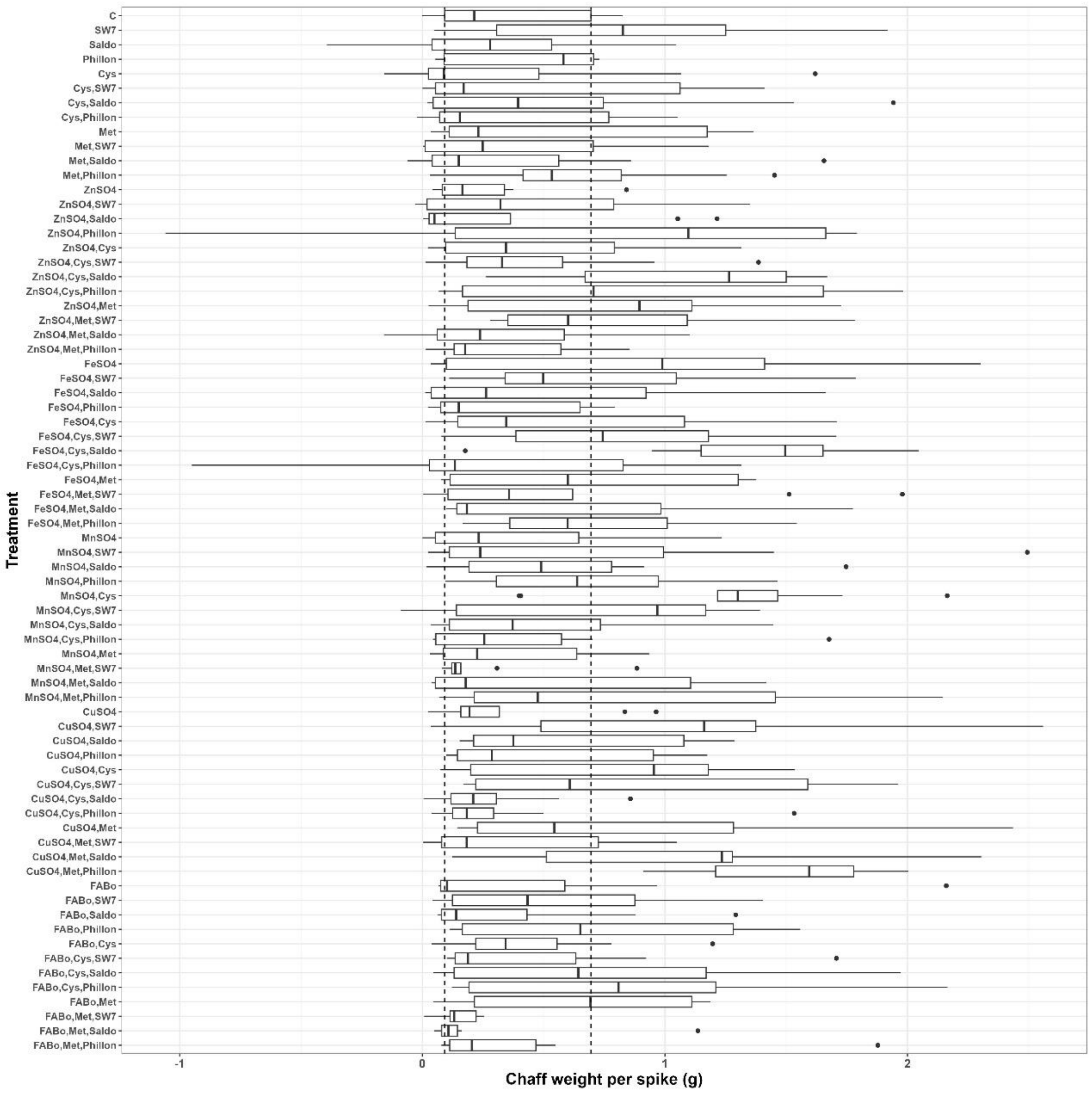

Figure S4a. Box plot diagram for chaff weight per spike, for each treatment (experimental year 2022-2023).

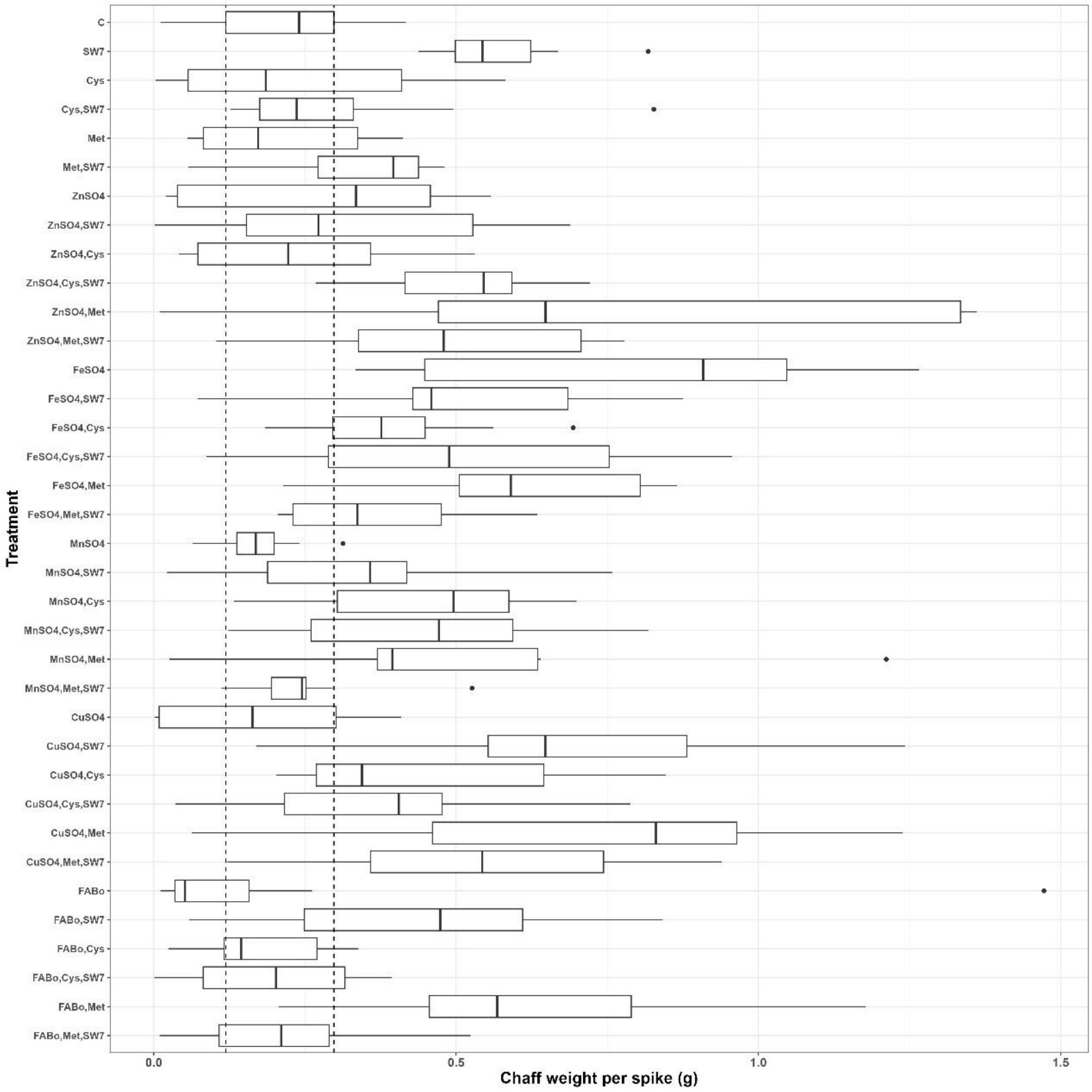

Figure S4b. Box plot diagram for chaff weight per spike, for each treatment (experimental year 2021-2022).

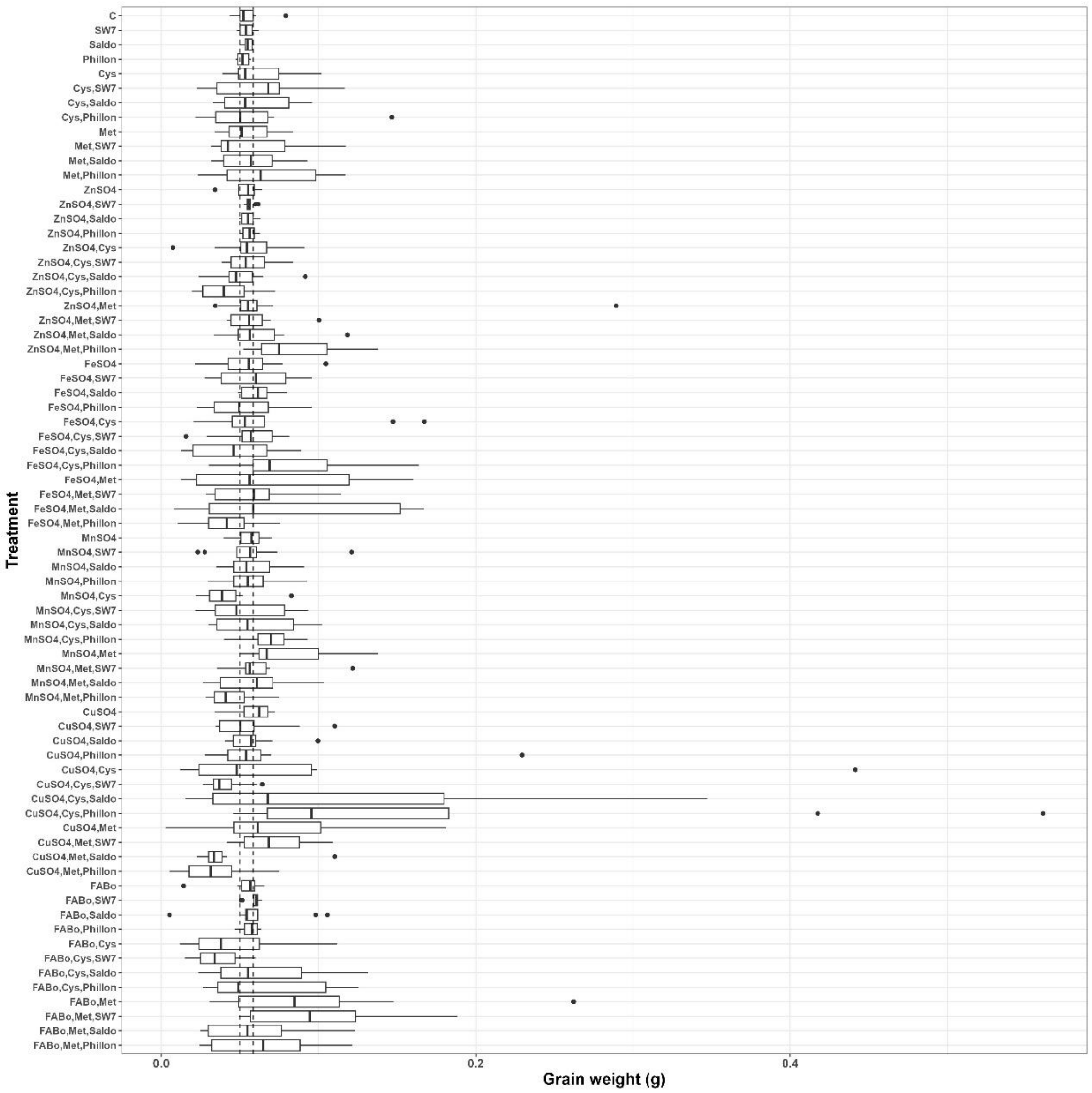

Figure S5a. Box plot diagram for weight per grain, for each treatment (experimental year 2022-2023).

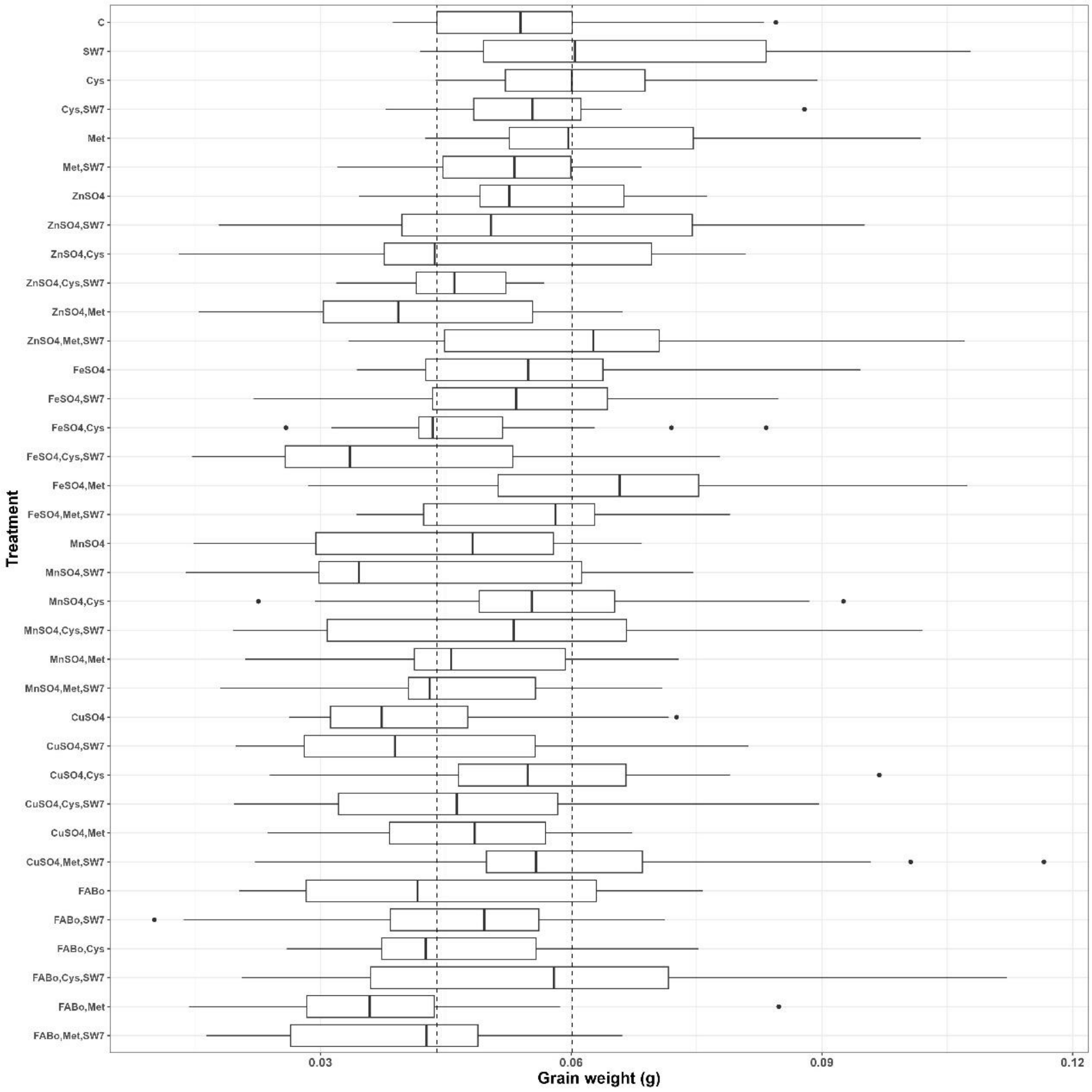

Figure S5b. Box plot diagram for weight per grain, for each treatment (experimental year 2021-2022).

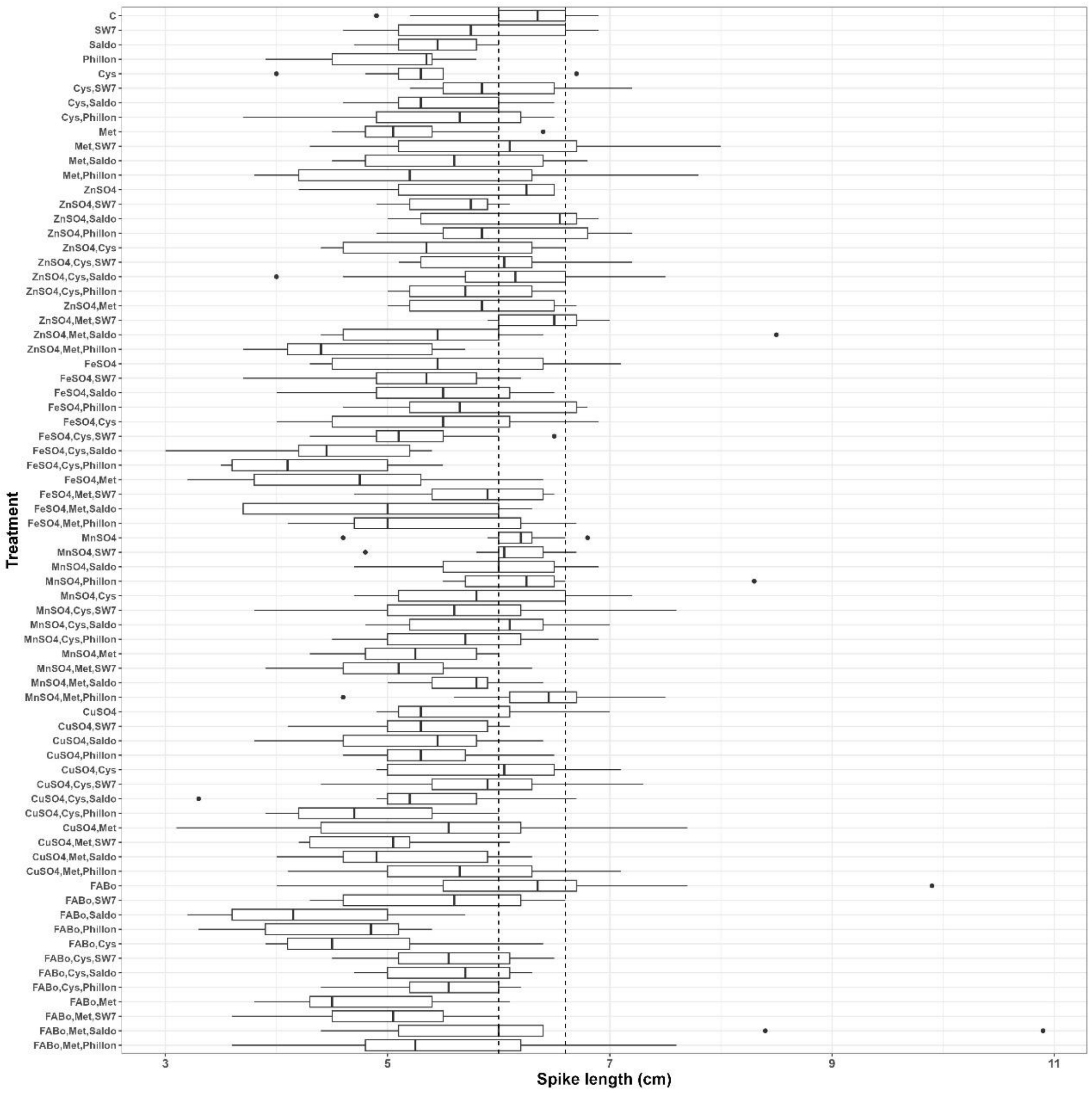

Figure S6a. Box plot diagram for spike length, for each treatment (experimental year 2022-2023).

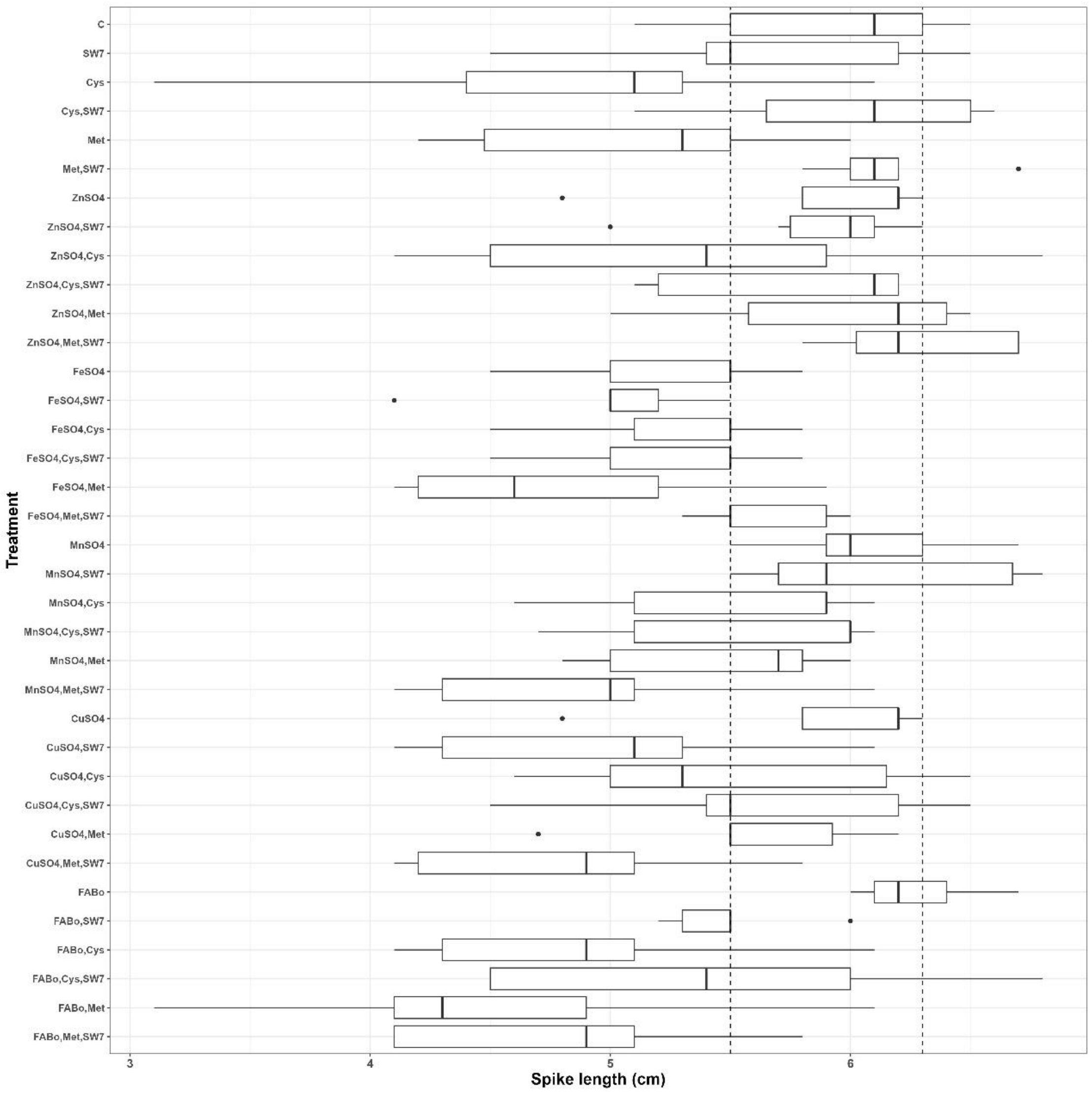

Figure S6b. Box plot diagram for spike length, for each treatment (experimental year 2021-2022).

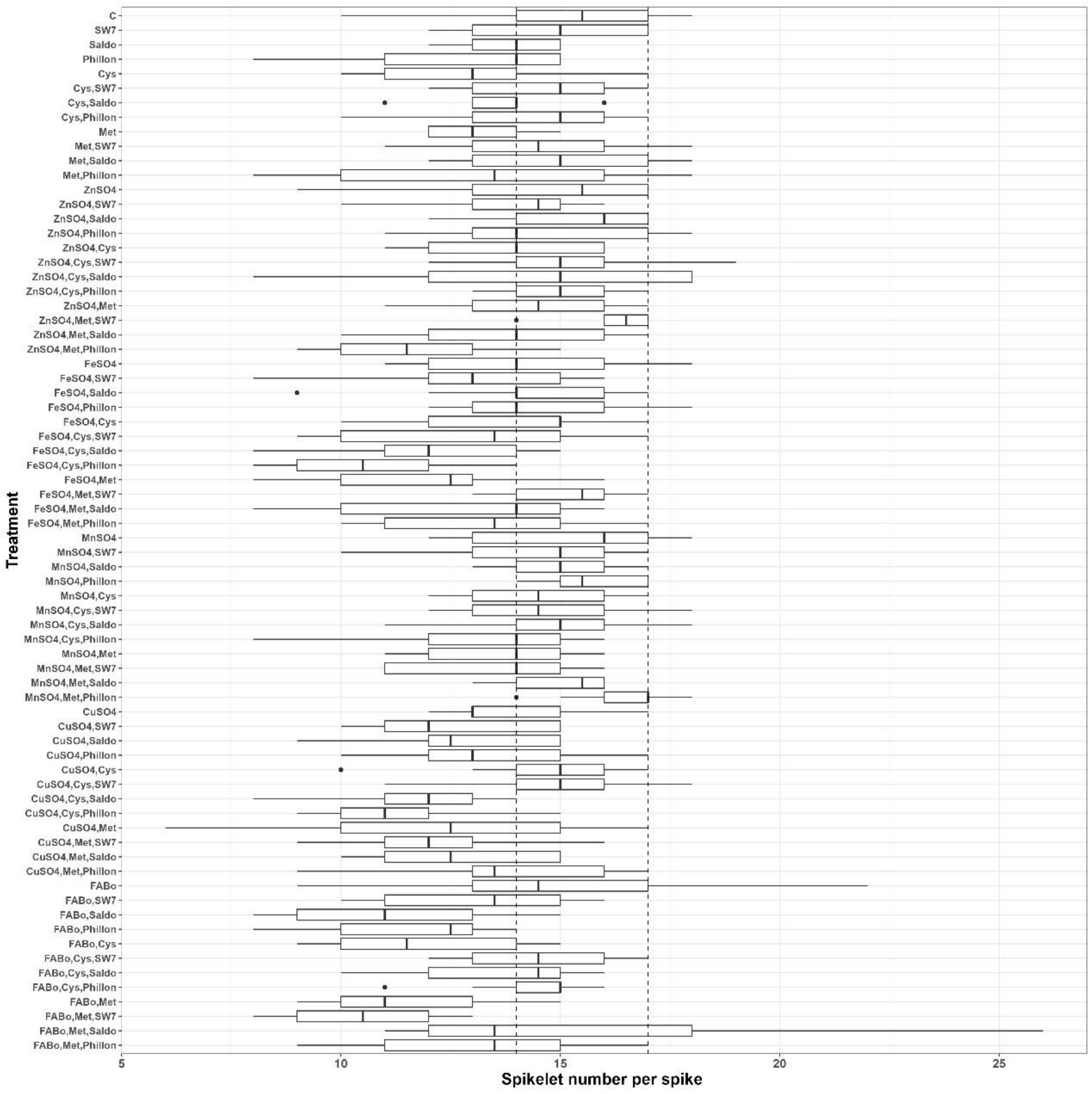

Figure S7a. Box plot diagram for spikelet number per spike, for each treatment (experimental year 2022-2023).

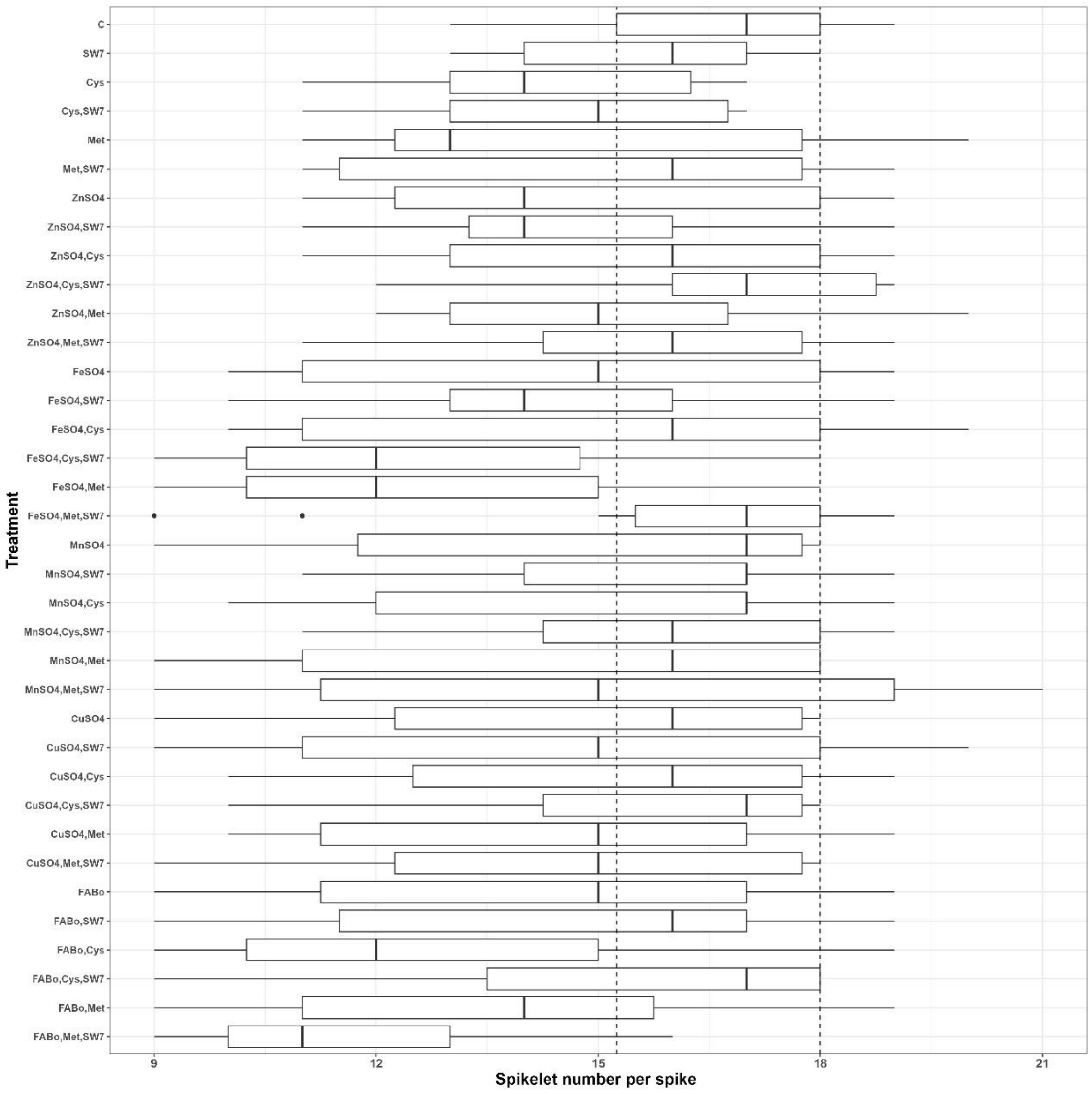

Figure S7b. Box plot diagram for spikelet number per spike, for each treatment (experimental year 2021-2022).

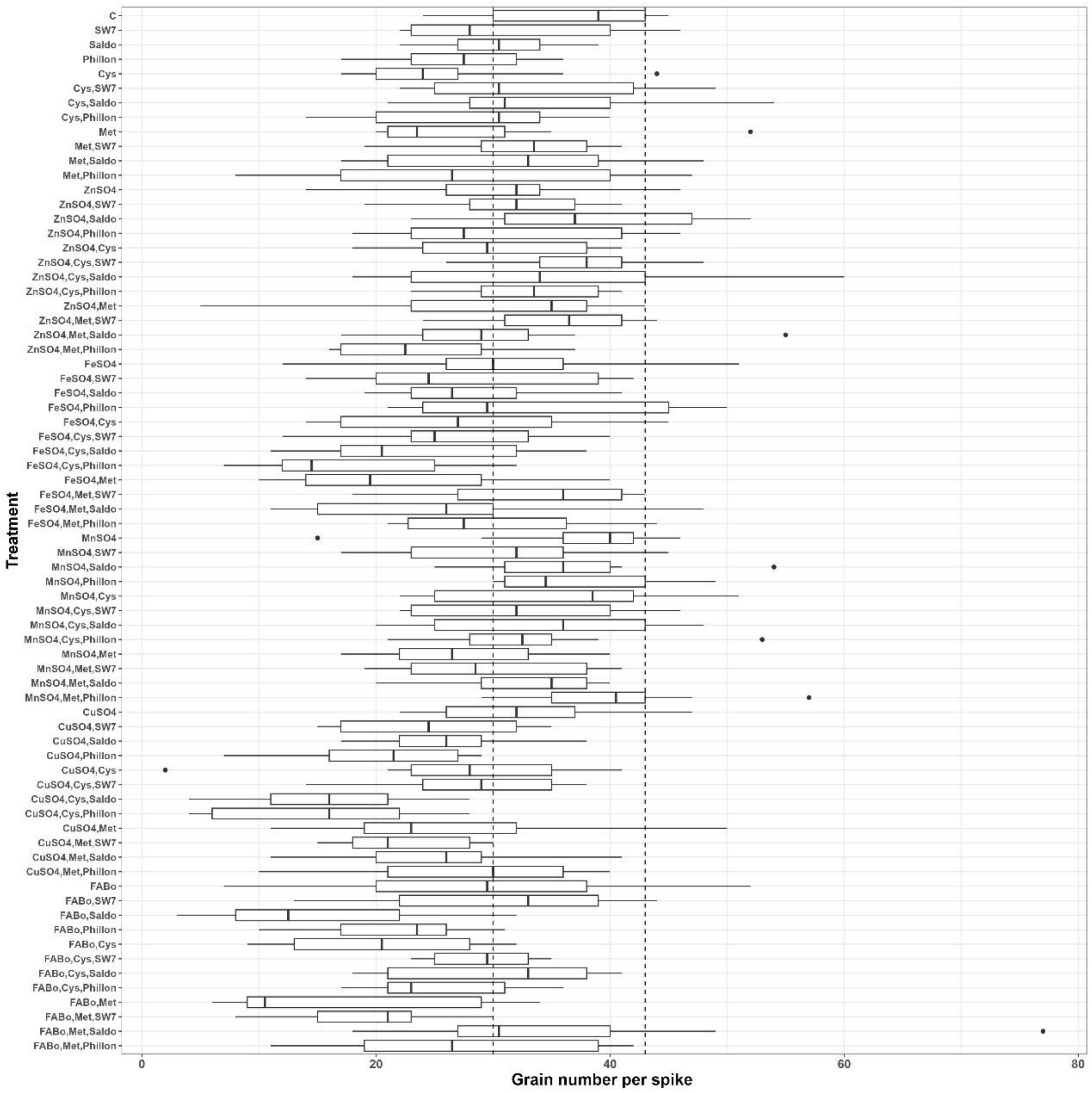

Figure S8a. Box plot diagram for grain number per spike, for each treatment (experimental year 2022-2023).

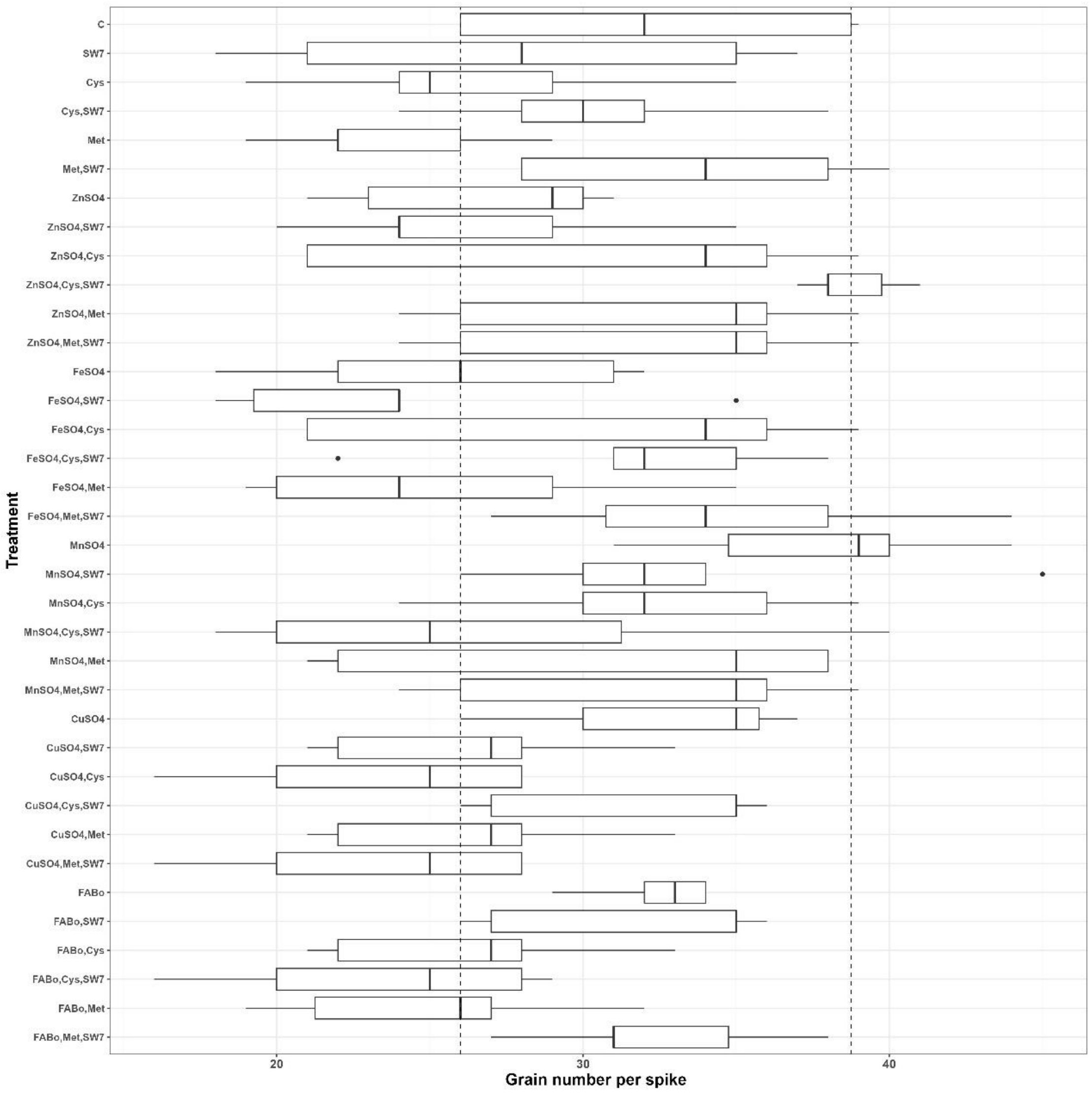

Figure S8b. Box plot diagram for grain number per spike, for each treatment (experimental year 2021-2022).

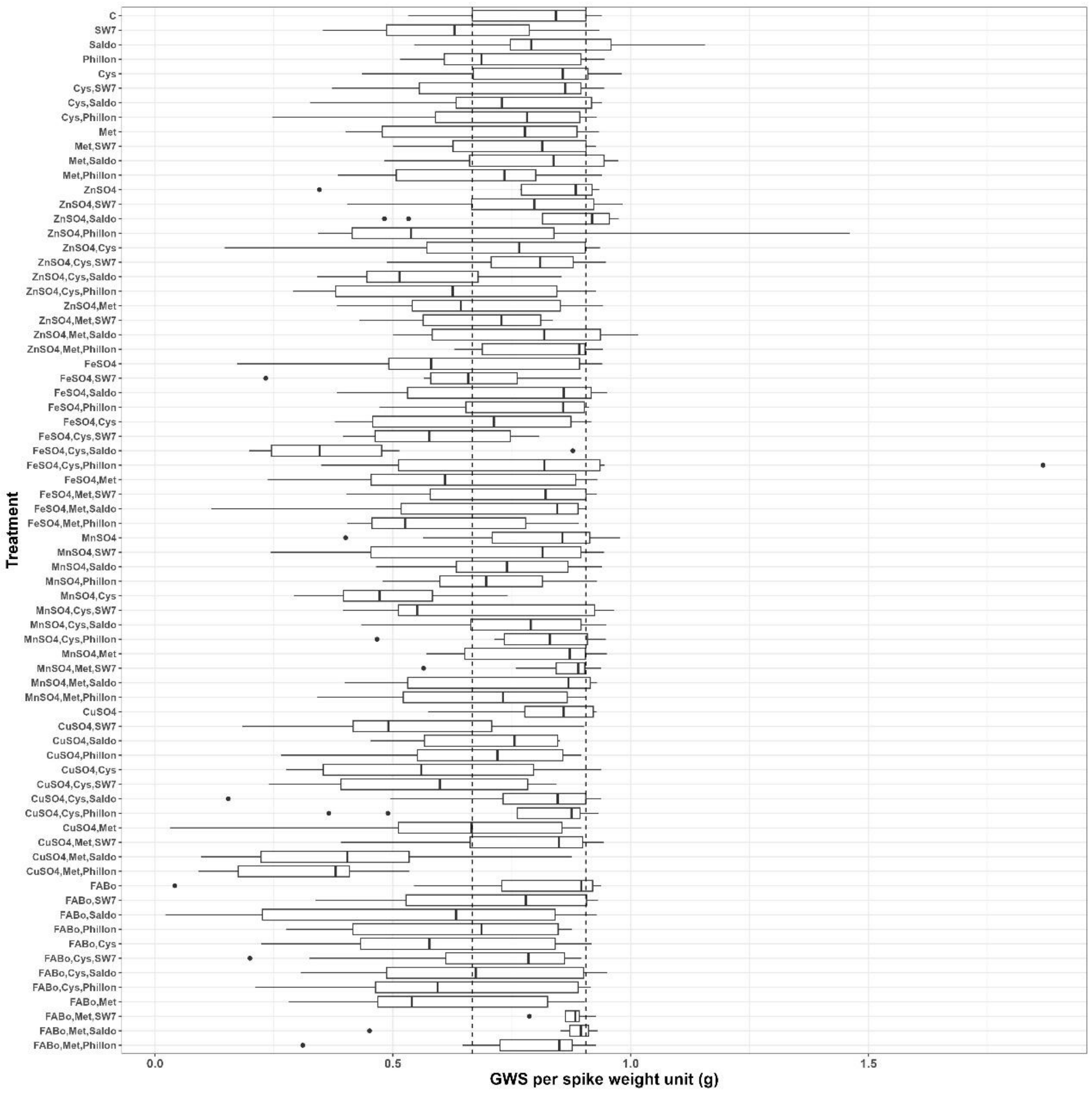

Figure S9a. Box plot diagram for grain weight per spike / spike weight ratio, for each treatment (experimental year 2022-2023).

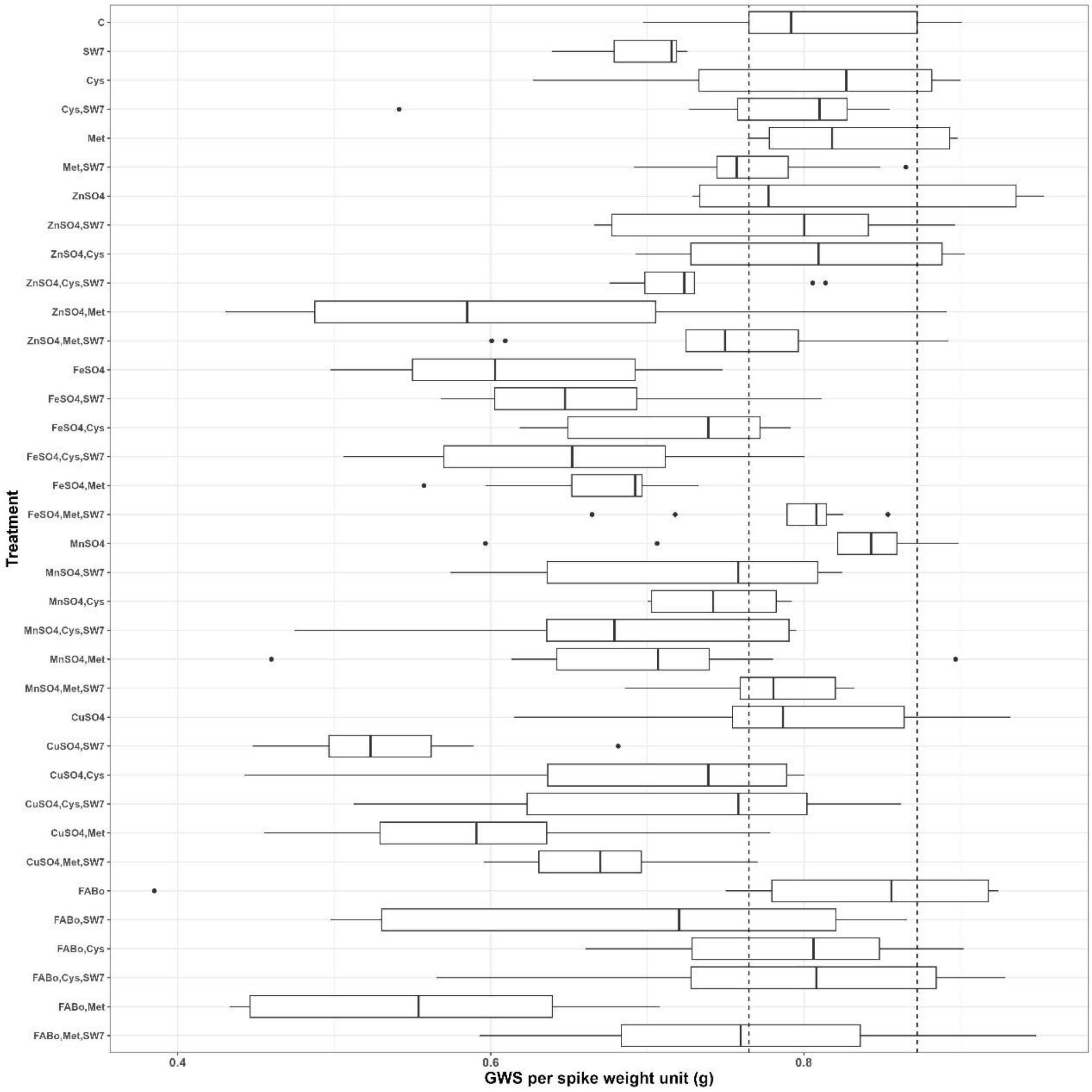

Figure S9b. Box plot diagram for grain weight per spike / spike weight ratio, for each treatment (experimental year 2021-2022).

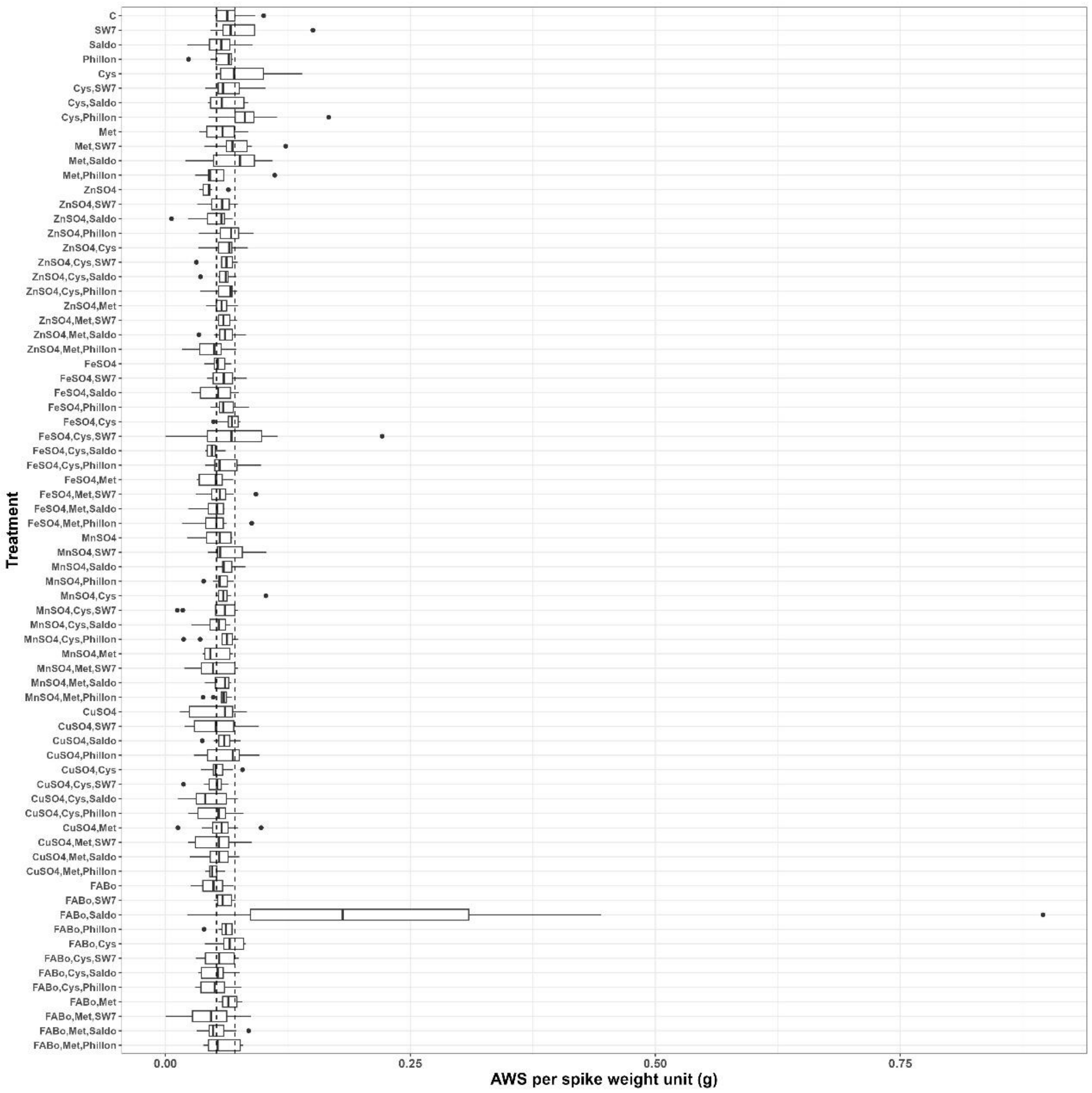

Figure S10a. Box plot diagram for awns weight per spike / spike length ratio, for each treatment (experimental year 2022-2023).

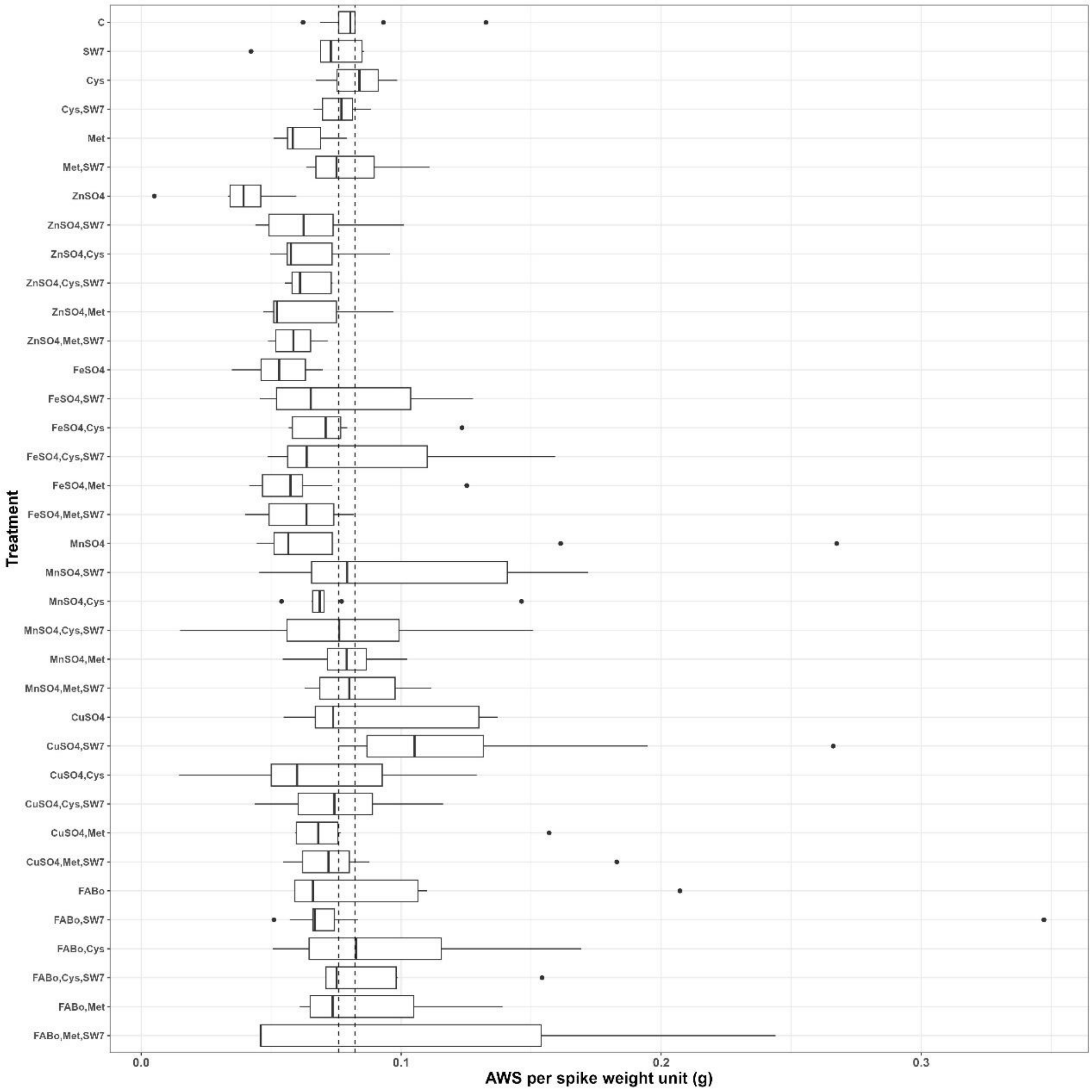

Figure S10b. Box plot diagram for awns weight per spike / spike length ratio, for each treatment (experimental year 2021-2022).

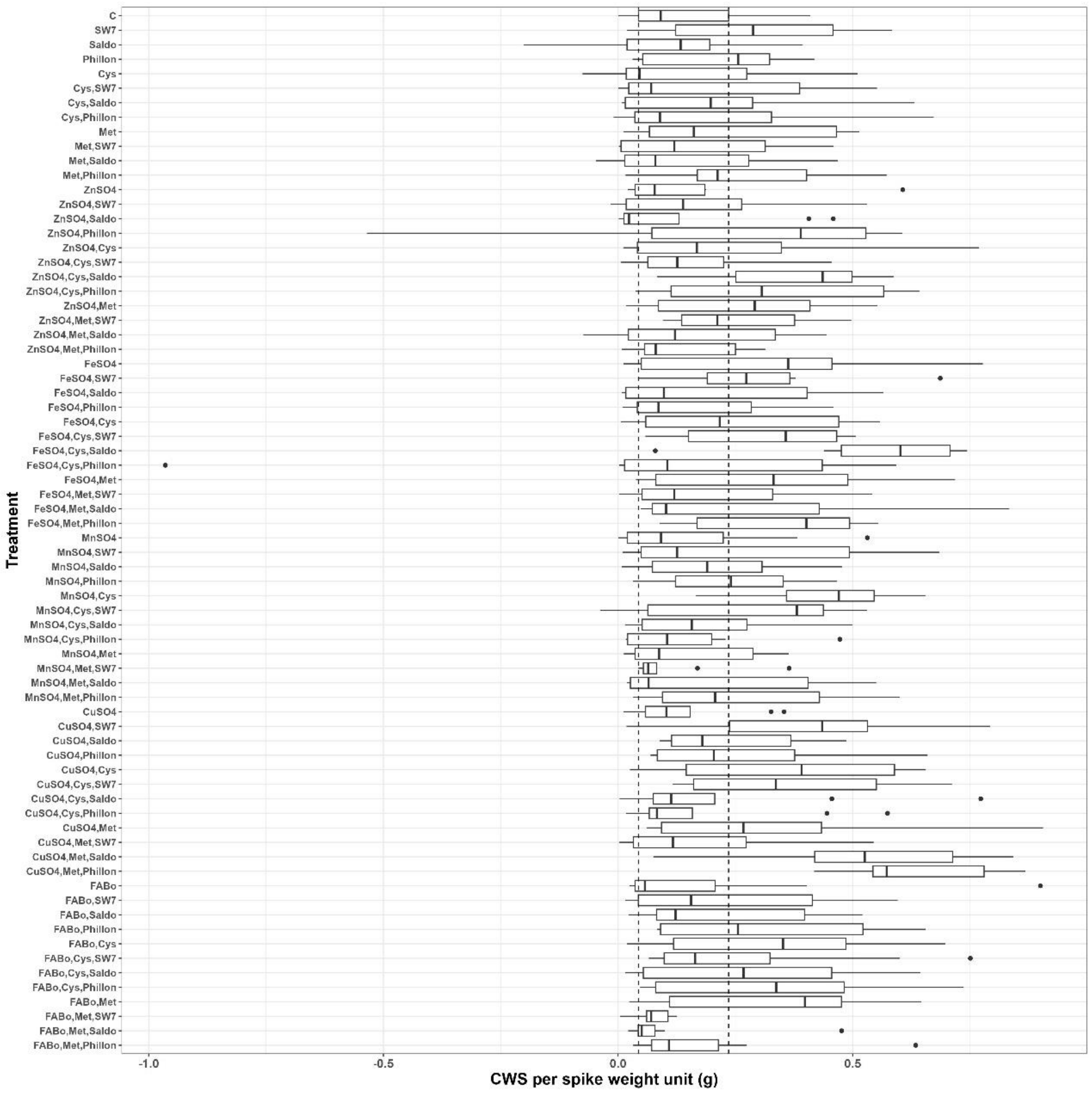

Figure S11a. Box plot diagram for chaff weight per spike / spike weight ratio, for each treatment (experimental year 2022-2023).

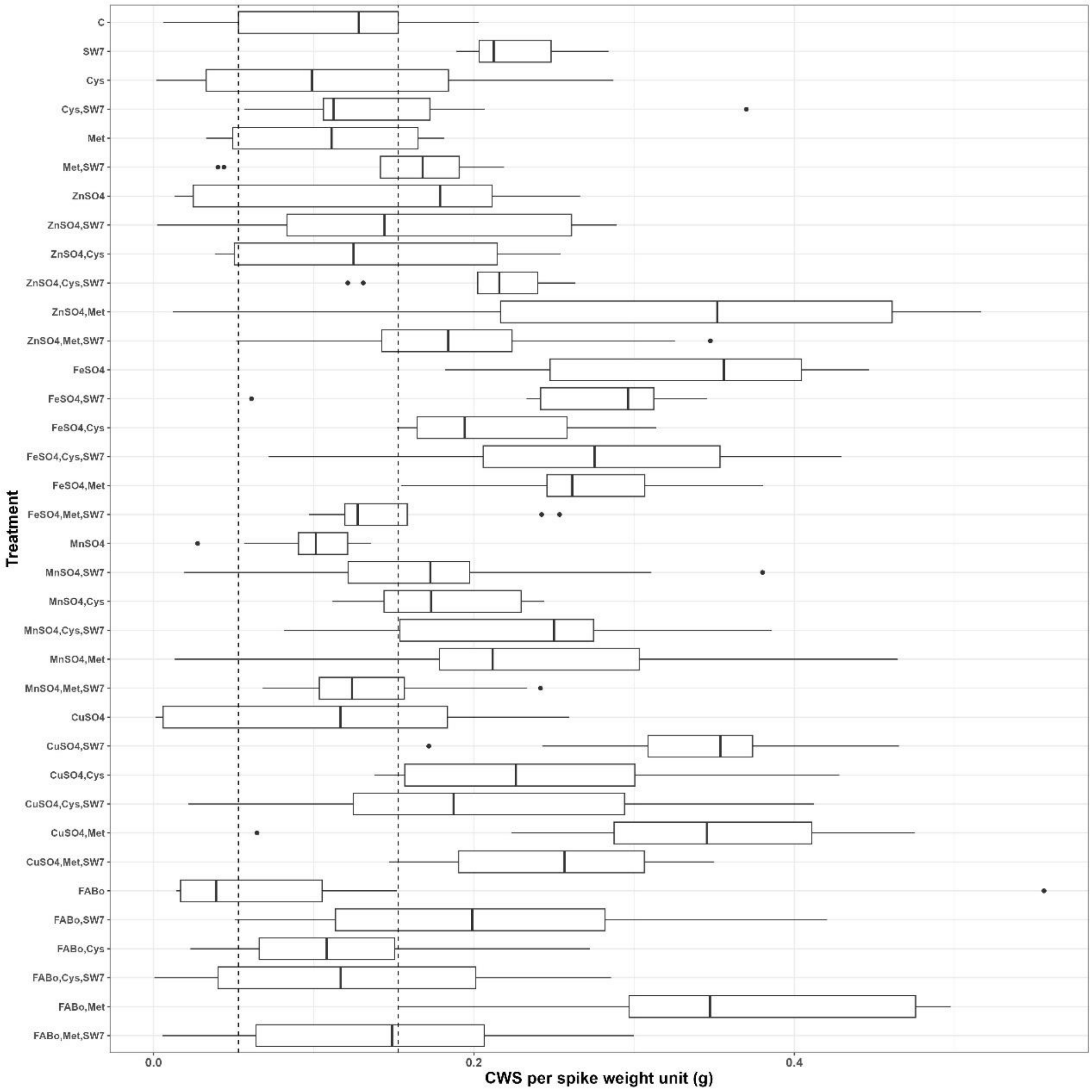

Figure S11b. Box plot diagram for chaff weight per spike / spike weight ratio, for each treatment (experimental year 2021-2022).

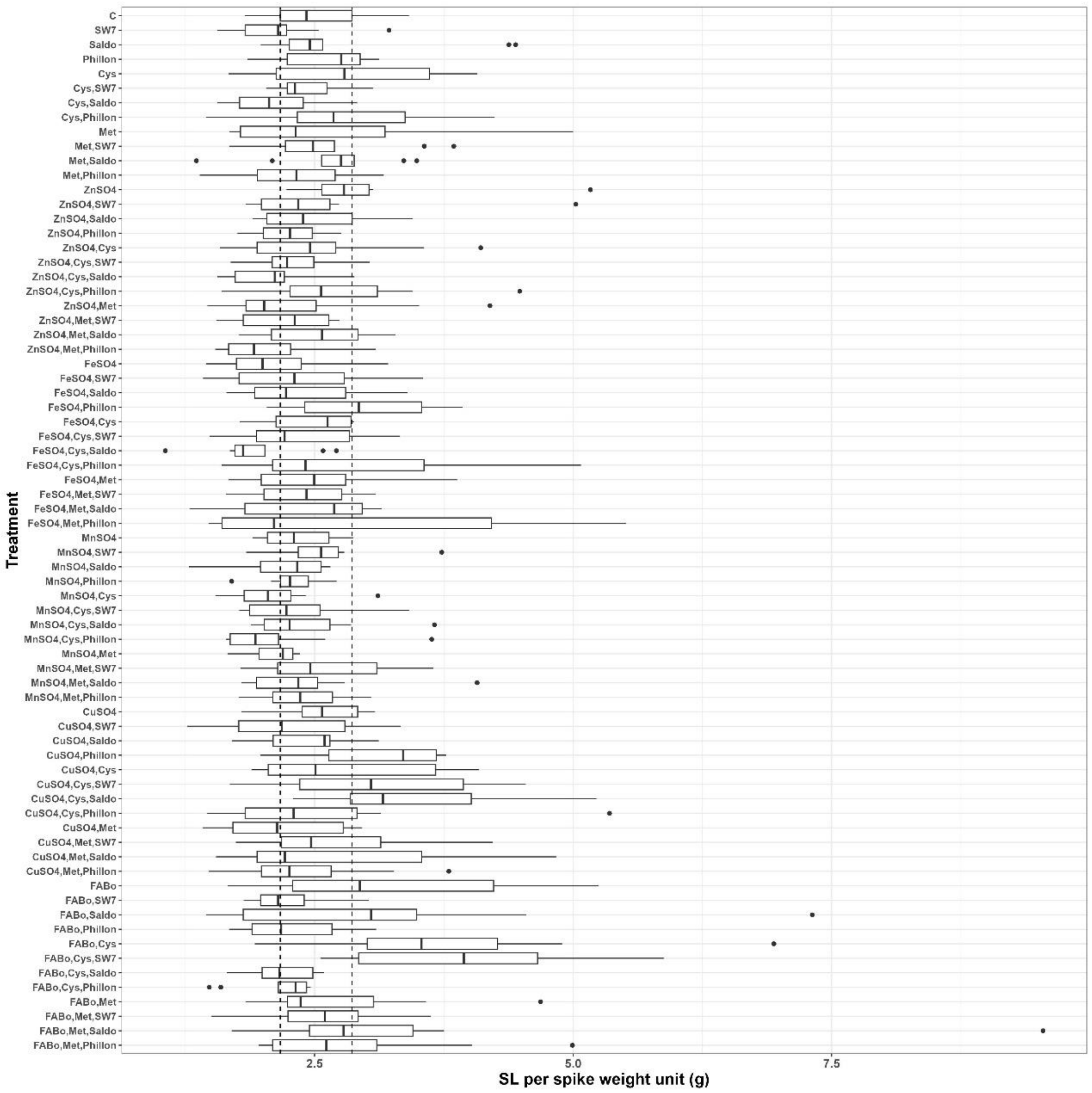

Figure S12a. Box plot diagram for spike length / spike weight ratio, for each treatment (experimental year 2022-2023).

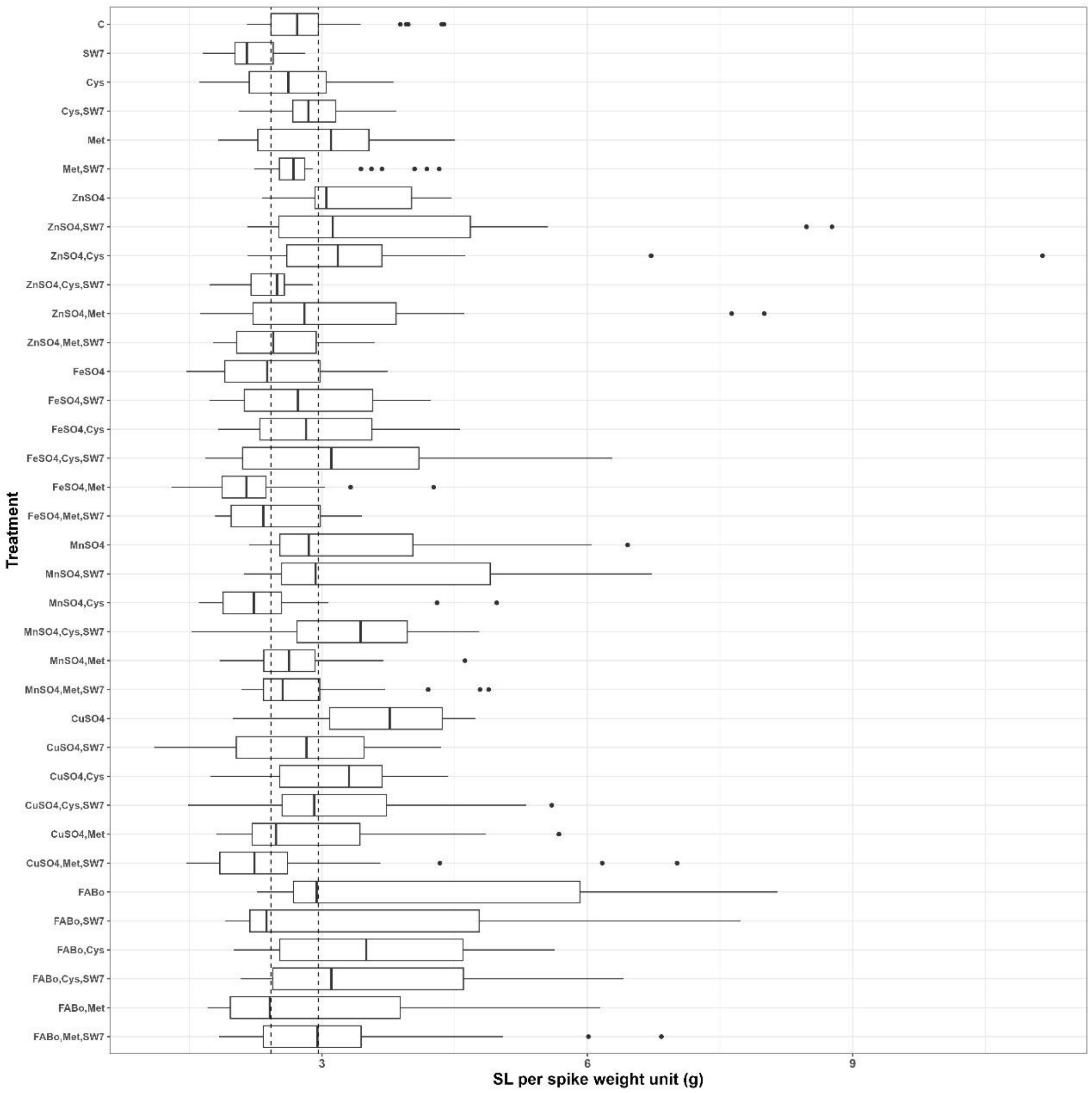

Figure S12b. Box plot diagram for spike length / spike weight ratio, for each treatment (experimental year 2021-2022).

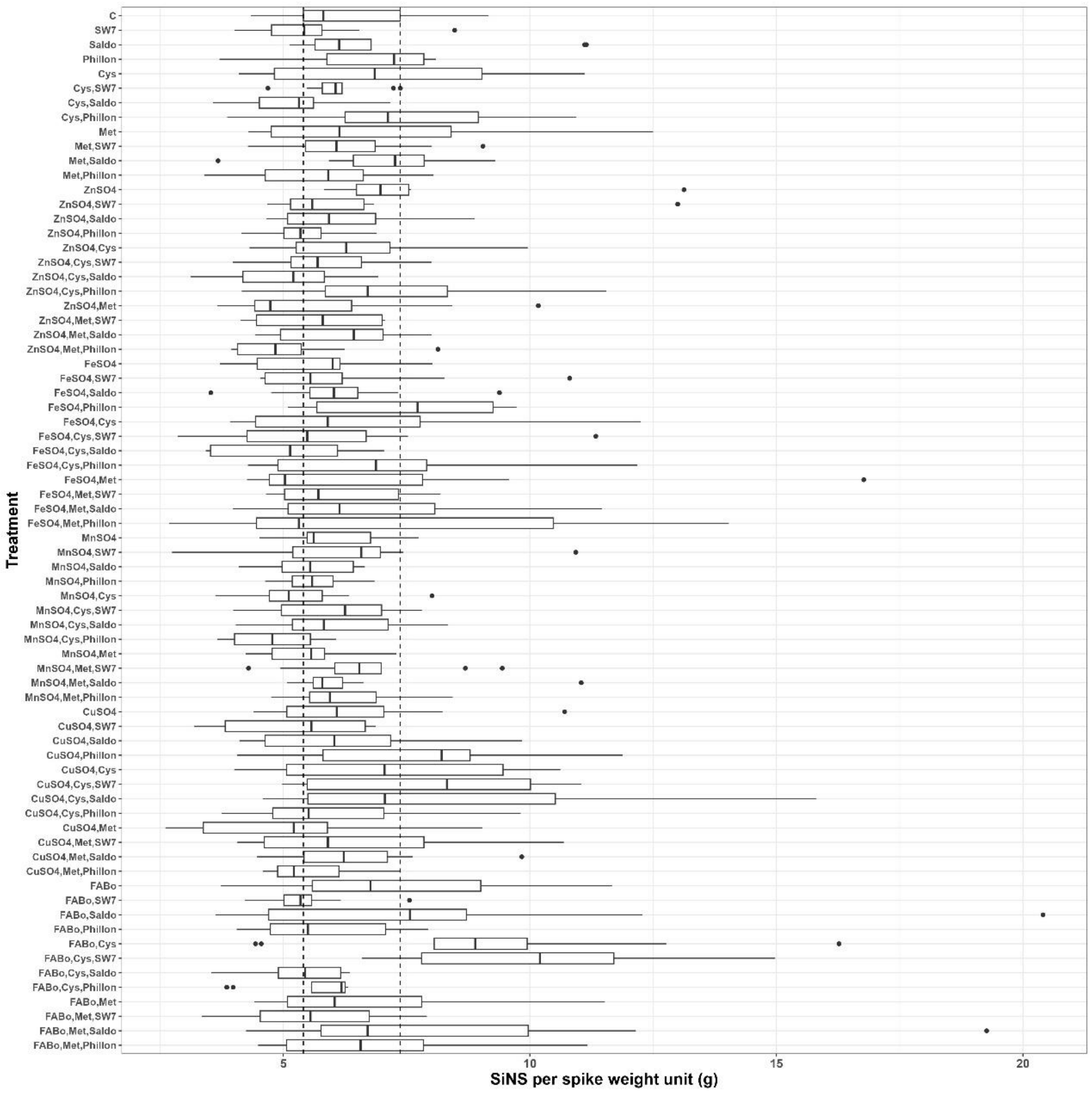

Figure S13a. Box plot diagram for spikelet number per spike / spike weight ratio, for each treatment (experimental year 2022-2023).

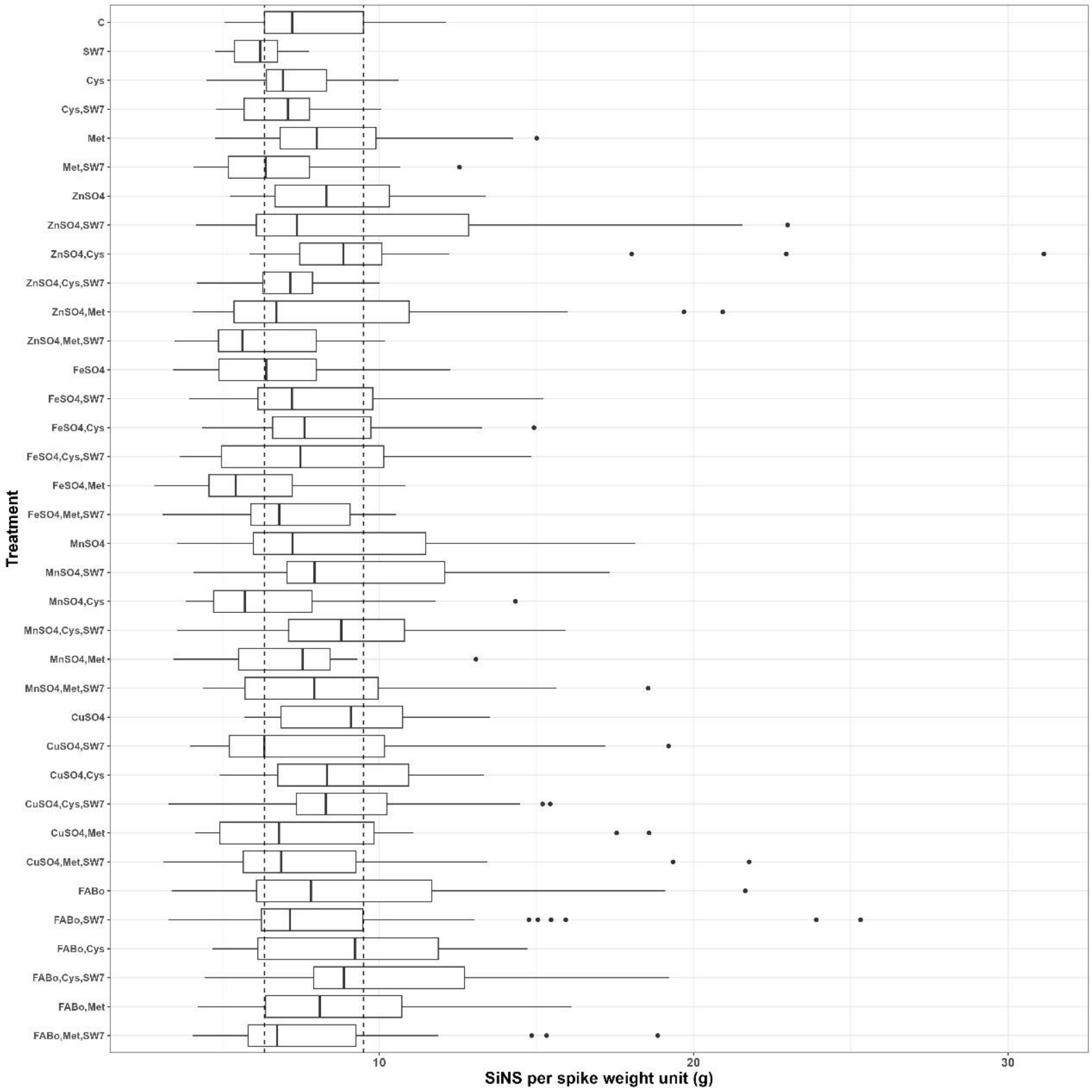

Figure S13b. Box plot diagram for spikelet number per spike / spike weight ratio, for each treatment (experimental year 2021-2022).

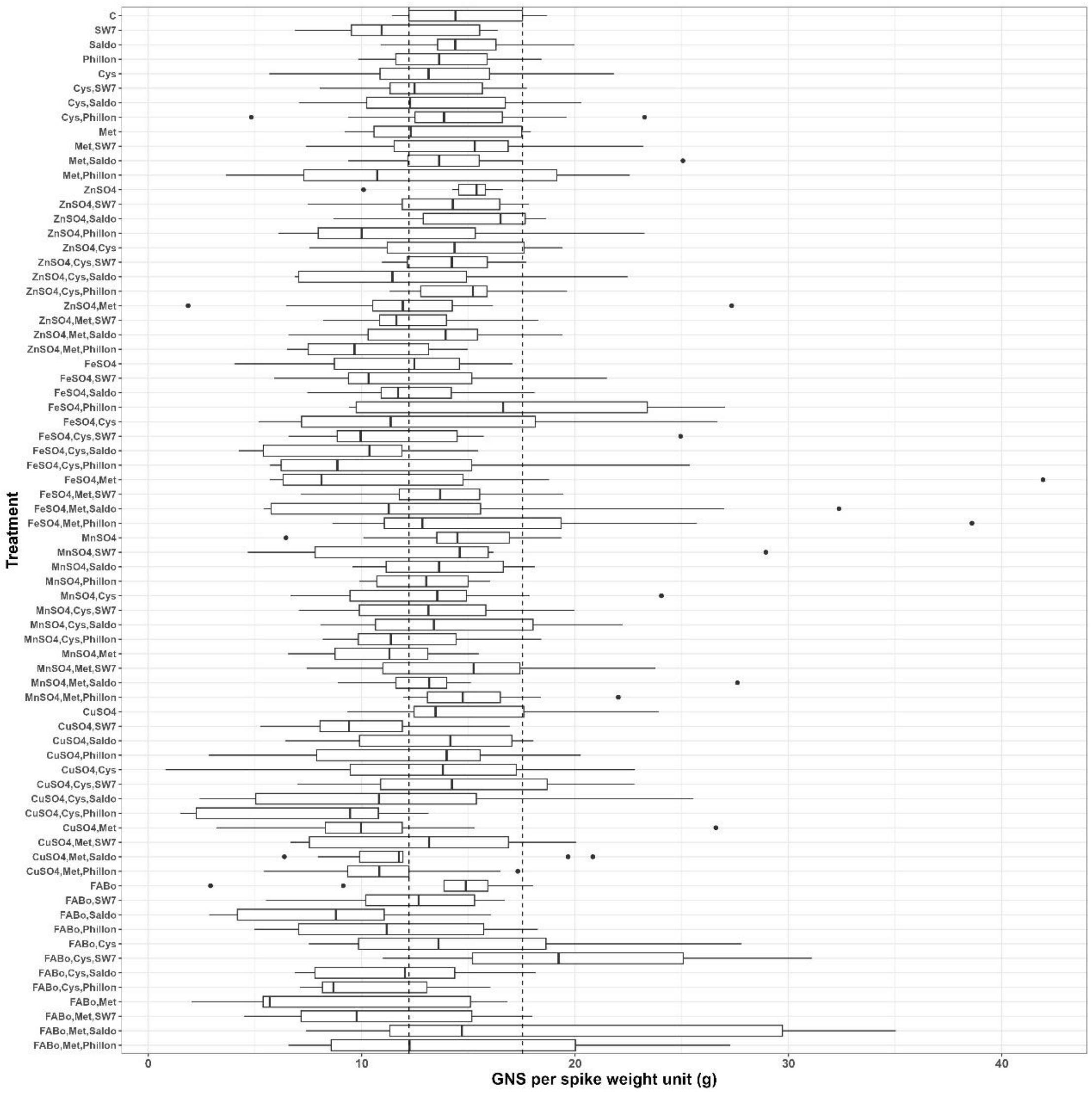

Figure S14a. Box plot diagram for grain number per spike / spike weight ratio, for each treatment (experimental year 2022-2023).

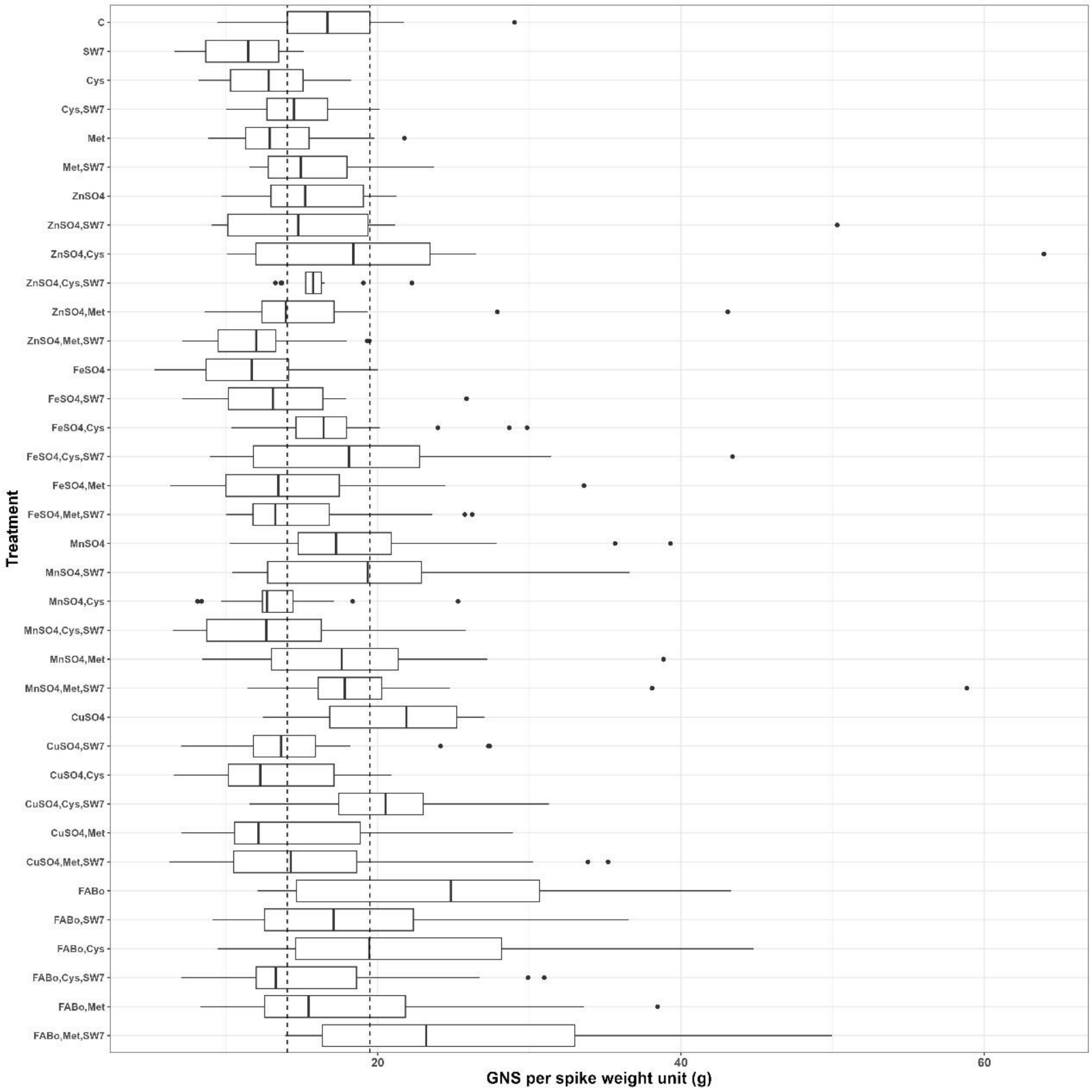

Figure S14b. Box plot diagram for grain number per spike / spike weight ratio, for each treatment (experimental year 2021-2022).

Table S1. Spike weight, grain weight per spike, awns weight per spike chaff weight per spike, weight per grain as affected by the treatments (experimental year 2021-2022). Mean: mean values; SD: standard deviation, Diff: the difference between the mean value of the treatment and that of the control, SC: significance code; ns: not statistically significant, * <0.05, **<0.01, ***<0.001.

Treatment	Spike weight (g)					Grain weight per spike (g)					Awns weight per spike (g)					Chaff weight per spike (g)					Grain weight (g)				
	mean	SD	Diff	p-value	SC	mean	SD	Diff	p-value	SC	mean	SD	Diff	p-value	SC	mean	SD	Diff	p-value	SC	mean	SD	Diff	p-value	SC
Control	2.142	0.410				1.745	0.404				0.175	0.030				0.222	0.120				0.054	0.013			
SW7	2.554	0.164	0.41	0.3492	ns	1.792	0.123	0.047	1.0000	ns	0.187	0.028	0.012	1.0000	ns	0.575	0.104	0.35	0.0000	***	0.067	0.021	0.013	0.4266	ns
Cys	1.966	0.266	-0.18	1.0000	ns	1.563	0.163	-0.182	0.9988	ns	0.163	0.033	-0.012	1.0000	ns	0.239	0.199	0.02	1.0000	ns	0.062	0.013	0.008	0.9990	ns
Cys x SW7	2.113	0.280	-0.03	1.0000	ns	1.645	0.282	-0.101	1.0000	ns	0.162	0.028	-0.013	1.0000	ns	0.306	0.203	0.09	1.0000	ns	0.055	0.011	0	1.0000	ns
Met	1.806	0.451	-0.34	0.8091	ns	1.495	0.364	-0.25	0.8721	ns	0.112	0.030	-0.063	0.0000	***	0.198	0.129	-0.02	1.0000	ns	0.064	0.016	0.01	0.8957	ns
Met x SW7	2.228	0.382	0.09	1.0000	ns	1.718	0.320	-0.027	1.0000	ns	0.173	0.034	-0.002	1.0000	ns	0.337	0.141	0.12	0.9925	ns	0.052	0.011	-0.002	1.0000	ns
ZnSO4	1.813	0.314	-0.33	0.8427	ns	1.472	0.165	-0.273	0.7281	ns	0.070	0.029	-0.105	0.0000	***	0.270	0.207	0.05	1.0000	ns	0.056	0.012	0.001	1.0000	ns
ZnSO4 x SW7	1.794	0.627	-0.35	0.7485	ns	1.364	0.431	-0.381	0.0693	ns	0.106	0.031	-0.069	0.0000	***	0.324	0.229	0.1	0.9991	ns	0.054	0.022	0	1.0000	ns
ZnSO4 x Cys	1.664	0.399	-0.48	0.0933	ns	1.331	0.327	-0.414	0.0216	*	0.102	0.024	-0.073	0.0000	***	0.231	0.161	0.01	1.0000	ns	0.048	0.019	-0.006	1.0000	ns
ZnSO4 x Cys x SW7	2.435	0.357	0.29	0.9561	ns	1.773	0.265	0.028	1.0000	ns	0.156	0.030	-0.019	0.9990	ns	0.506	0.137	0.28	0.0006	***	0.046	0.007	-0.008	0.9967	ns
ZnSO4 x Met	2.109	0.736	-0.03	1.0000	ns	1.245	0.408	-0.5	0.0006	***	0.120	0.028	-0.055	0.0001	***	0.744	0.450	0.52	0.0000	***	0.041	0.015	-0.013	0.4361	ns
ZnSO4 x Met x SW7	2.600	0.551	0.46	0.1480	ns	1.962	0.502	0.217	0.9774	ns	0.153	0.033	-0.022	0.9796	ns	0.486	0.227	0.26	0.0032	**	0.065	0.023	0.011	0.8529	ns
FeSO4	2.332	0.591	0.19	1.0000	ns	1.400	0.337	-0.345	0.1966	ns	0.124	0.033	-0.051	0.0008	***	0.808	0.313	0.59	0.0000	***	0.056	0.015	0.002	1.0000	ns
FeSO4 x SW7	1.885	0.464	-0.26	0.9937	ns	1.224	0.269	-0.521	0.0002	***	0.134	0.032	-0.041	0.0480	*	0.527	0.230	0.31	0.0001	***	0.054	0.016	0	1.0000	ns
FeSO4 x Cys	1.888	0.440	-0.25	0.9948	ns	1.356	0.339	-0.389	0.0527	ns	0.135	0.032	-0.041	0.0546	ns	0.398	0.140	0.18	0.4425	ns	0.047	0.013	-0.007	0.9998	ns
FeSO4 x Cys x SW7	1.834	0.628	-0.31	0.9201	ns	1.180	0.415	-0.565	0.0000	***	0.131	0.025	-0.045	0.0137	*	0.523	0.293	0.3	0.0002	***	0.039	0.017	-0.015	0.2094	ns
FeSO4 x Met	2.240	0.440	0.1	1.0000	ns	1.503	0.317	-0.242	0.9077	ns	0.130	0.021	-0.045	0.0102	*	0.608	0.189	0.39	0.0000	***	0.064	0.021	0.01	0.9628	ns
FeSO4 x Met x SW7	2.383	0.505	0.24	0.9978	ns	1.882	0.442	0.137	1.0000	ns	0.142	0.024	-0.033	0.3488	ns	0.360	0.148	0.14	0.9108	ns	0.055	0.012	0.001	1.0000	ns
MnSO4	1.922	0.591	-0.22	0.9996	ns	1.608	0.589	-0.137	1.0000	ns	0.138	0.053	-0.037	0.1517	ns	0.176	0.065	-0.05	1.0000	ns	0.044	0.018	-0.01	0.8925	ns
MnSO4 x SW7	1.865	0.604	-0.28	0.9797	ns	1.371	0.501	-0.374	0.0866	ns	0.160	0.039	-0.016	1.0000	ns	0.334	0.202	0.11	0.9950	ns	0.042	0.018	-0.012	0.7062	ns
MnSO4 x Cys	2.408	0.535	0.27	0.9890	ns	1.786	0.385	0.041	1.0000	ns	0.171	0.024	-0.004	1.0000	ns	0.450	0.173	0.23	0.0374	*	0.058	0.017	0.004	1.0000	ns
MnSO4 x Cys x SW7	1.835	0.581	-0.31	0.9237	ns	1.262	0.412	-0.483	0.0012	**	0.143	0.066	-0.032	0.4837	ns	0.429	0.212	0.21	0.1187	ns	0.052	0.022	-0.002	1.0000	ns
MnSO4 x Met	2.118	0.428	-0.02	1.0000	ns	1.472	0.350	-0.274	0.7219	ns	0.164	0.033	-0.011	1.0000	ns	0.483	0.299	0.26	0.0039	**	0.049	0.013	-0.005	1.0000	ns
MnSO4 x Met x SW7	1.832	0.336	-0.31	0.9143	ns	1.424	0.279	-0.321	0.3404	ns	0.155	0.043	-0.02	0.9977	ns	0.253	0.105	0.03	1.0000	ns	0.047	0.013	-0.007	0.9997	ns
CuSO4	1.664	0.346	-0.48	0.0950	ns	1.341	0.382	-0.404	0.0316	*	0.149	0.044	-0.026	0.8847	ns	0.174	0.146	-0.05	1.0000	ns	0.041	0.013	-0.013	0.5506	ns
CuSO4 x SW7	2.075	0.767	-0.07	1.0000	ns	1.136	0.501	-0.609	0.0000	***	0.230	0.050	0.055	0.0002	***	0.709	0.296	0.49	0.0000	***	0.044	0.019	-0.01	0.9238	ns
CuSO4 x Cys	1.851	0.441	-0.29	0.9605	ns	1.273	0.302	-0.472	0.0020	**	0.127	0.062	-0.048	0.0032	**	0.451	0.220	0.23	0.0343	*	0.056	0.015	0.002	1.0000	ns
CuSO4 x Cys x SW7	1.942	0.577	-0.2	1.0000	ns	1.428	0.515	-0.318	0.3673	ns	0.145	0.056	-0.03	0.5893	ns	0.370	0.210	0.15	0.8225	ns	0.046	0.018	-0.008	0.9947	ns
CuSO4 x Met	2.109	0.523	-0.03	1.0000	ns	1.216	0.280	-0.529	0.0001	***	0.150	0.023	-0.025	0.9169	ns	0.743	0.326	0.52	0.0000	***	0.047	0.012	-0.007	0.9998	ns
CuSO4 x Met x SW7	2.081	0.633	-0.06	1.0000	ns	1.378	0.398	-0.367	0.1062	ns	0.154	0.038	-0.021	0.9925	ns	0.549	0.256	0.33	0.0000	***	0.061	0.022	0.007	0.9994	ns
FABo	1.811	0.669	-0.33	0.8356	ns	1.440	0.584	-0.306	0.4614	ns	0.147	0.062	-0.028	0.7488	ns	0.225	0.430	0	1.0000	ns	0.045	0.019	-0.009	0.9774	ns
FABo x SW7	1.989	0.699	-0.15	1.0000	ns	1.410	0.597	-0.335	0.2481	ns	0.152	0.050	-0.023	0.9693	ns	0.428	0.242	0.21	0.1289	ns	0.044	0.017	-0.01	0.9600	ns
FABo x Cys	1.479	0.367	-0.66	0.0003	***	1.174	0.330	-0.571	0.0000	***	0.132	0.044	-0.043	0.0226	*	0.173	0.102	-0.05	1.0000	ns	0.045	0.013	-0.009	0.9886	ns
FABo x Cys x SW7	1.654	0.504	-0.49	0.0745	ns	1.325	0.511	-0.42	0.0174	*	0.138	0.038	-0.037	0.1587	ns	0.191	0.129	-0.03	1.0000	ns	0.058	0.026	0.004	1.0000	ns
FABo x Met	1.657	0.489	-0.48	0.0805	ns	0.908	0.303	-0.837	0.0000	***	0.140	0.043	-0.035	0.2329	ns	0.610	0.284	0.39	0.0000	***	0.038	0.017	-0.016	0.1097	ns
FABo x Met x SW7	1.615	0.460	-0.53	0.0263	*	1.257	0.452	-0.488	0.0010	***	0.144	0.089	-0.031	0.5526	ns	0.213	0.149	-0.01	1.0000	ns	0.039	0.014	-0.015	0.1645	ns

Table S2. Spike length, spikelet number per spike, and grain number per spike as affected by the treatments (experimental year 2021-2022). Mean: mean values; SD: standard deviation, Diff: the difference between the mean value of the treatment and that of the control, SC: significance code; ns: not statistically significant, * <0.05, **<0.01, ***<0.001.

Treatment	Spike length (cm)					Spikelet number per spike					Grain number per spike				
	mean	SD	Diff	p-value	SC	mean	SD	Diff	p-value	SC	mean	SD	Diff	p-value	SC
Control	5.94	0.43				16.4	1.74				32.7	5.95			
SW7	5.59	0.67	-0.35	0.9158	ns	15.6	1.77	-0.833	1.0000	ns	28.7	7.10	-4	0.4222	ns
Cys	5.01	0.72	-0.94	0.0000	***	13.9	2.15	-2.5	0.2142	ns	26.0	4.00	-6.667	0.0002	***
Cys x SW7	6.03	0.50	0.09	1.0000	ns	14.5	2.13	-1.9	0.8190	ns	30.5	3.96	-2.133	0.9998	ns
Met	5.13	0.63	-0.82	0.0000	***	14.7	2.98	-1.7	0.9438	ns	23.4	2.65	-9.267	0.0000	***
Met x SW7	6.16	0.30	0.217	1.0000	ns	14.9	2.87	-1.567	0.9810	ns	33.7	4.69	1.067	1.0000	ns
ZnSO4	5.92	0.48	-0.02	1.0000	ns	15.1	2.96	-1.367	0.9980	ns	27.2	3.76	-5.467	0.0149	*
ZnSO4 x SW7	5.87	0.39	-0.08	1.0000	ns	14.8	2.44	-1.633	0.9662	ns	26.6	5.20	-6.067	0.0021	**
ZnSO4 x Cys	5.43	0.97	-0.51	0.1704	ns	15.4	2.90	-1.033	1.0000	ns	29.9	7.43	-2.8	0.9763	ns
ZnSO4 x Cys x SW7	5.79	0.48	-0.16	1.0000	ns	17.0	1.86	0.567	1.0000	ns	38.7	1.26	6.067	0.0021	**
ZnSO4 x Met	6.00	0.52	0.057	1.0000	ns	15.3	2.45	-1.1	1.0000	ns	31.5	5.58	-1.133	1.0000	ns
ZnSO4 x Met x SW7	6.30	0.35	0.357	0.8967	ns	15.7	2.34	-0.767	1.0000	ns	31.5	5.58	-1.133	1.0000	ns
FeSO4	5.33	0.42	-0.62	0.0153	*	14.7	3.38	-1.767	0.9123	ns	25.6	4.91	-7.067	0.0000	***
FeSO4 x SW7	5.00	0.40	-0.94	0.0000	***	14.4	2.46	-2.033	0.6896	ns	23.9	6.06	-8.733	0.0000	***
FeSO4 x Cys	5.33	0.40	-0.61	0.0182	*	15.0	3.60	-1.433	0.9953	ns	29.9	7.43	-2.8	0.9763	ns
FeSO4 x Cys x SW7	5.33	0.42	-0.62	0.0153	*	12.5	2.62	-3.9	0.0001	***	31.3	5.40	-1.333	1.0000	ns
FeSO4 x Met	4.85	0.66	-1.09	0.0000	***	12.9	2.91	-3.5	0.0016	**	25.1	5.87	-7.533	0.0000	***
FeSO4 x Met x SW7	5.64	0.27	-0.3	0.9872	ns	16.2	3.18	-0.233	1.0000	ns	34.5	5.10	1.8	1.0000	ns
MnSO4	6.07	0.40	0.123	1.0000	ns	14.9	3.38	-1.567	0.9810	ns	37.7	3.89	5.067	0.0459	*
MnSO4 x SW7	6.15	0.53	0.203	1.0000	ns	15.9	2.12	-0.5	1.0000	ns	33.5	6.42	0.867	1.0000	ns
MnSO4 x Cys	5.47	0.58	-0.48	0.2987	ns	15.0	3.26	-1.433	0.9953	ns	32.1	4.94	-0.533	1.0000	ns
MnSO4 x Cys x SW7	5.63	0.52	-0.32	0.9761	ns	15.5	2.62	-0.9	1.0000	ns	26.1	7.36	-6.6	0.0003	***
MnSO4 x Met	5.47	0.46	-0.47	0.3300	ns	14.6	3.46	-1.833	0.8707	ns	31.2	7.25	-1.467	1.0000	ns
MnSO4 x Met x SW7	4.95	0.70	-0.99	0.0000	***	14.8	3.83	-1.633	0.9662	ns	31.5	5.58	-1.133	1.0000	ns
CuSO4	5.92	0.48	-0.02	1.0000	ns	14.9	2.99	-1.567	0.9810	ns	32.9	4.06	0.267	1.0000	ns
CuSO4 x SW7	5.01	0.71	-0.94	0.0000	***	14.7	3.76	-1.767	0.9123	ns	26.3	3.87	-6.333	0.0008	***
CuSO4 x Cys	5.52	0.64	-0.42	0.5835	ns	15.1	2.90	-1.3	0.9992	ns	23.1	4.58	-9.533	0.0000	***
CuSO4 x Cys x SW7	5.59	0.67	-0.35	0.9158	ns	15.5	2.73	-0.9	1.0000	ns	31.6	4.15	-1.067	1.0000	ns
CuSO4 x Met	5.54	0.49	-0.4	0.6951	ns	14.8	3.02	-1.633	0.9662	ns	26.3	3.87	-6.333	0.0008	***
CuSO4 x Met x SW7	4.79	0.64	-1.16	0.0000	***	14.7	3.25	-1.767	0.9123	ns	23.1	4.58	-9.533	0.0000	***
FABo	6.28	0.25	0.337	0.9465	ns	14.5	3.13	-1.9	0.8190	ns	32.3	1.84	-0.4	1.0000	ns
FABo x SW7	5.48	0.24	-0.46	0.3629	ns	14.8	3.28	-1.633	0.9662	ns	31.6	4.15	-1.067	1.0000	ns
FABo x Cys	4.94	0.70	-1	0.0000	***	12.8	3.07	-3.633	0.0007	***	26.3	3.87	-6.333	0.0008	***
FABo x Cys x SW7	5.54	0.87	-0.4	0.6951	ns	15.5	3.10	-0.967	1.0000	ns	24.1	4.96	-8.6	0.0000	***
FABo x Met	4.49	1.00	-1.46	0.0000	***	13.5	3.10	-2.9	0.0417	*	24.6	4.08	-8.067	0.0000	***
FABo x Met x SW7	4.77	0.66	-1.17	0.0000	***	11.7	2.02	-4.767	0.0000	***	32.5	3.26	-0.133	1.0000	ns

Table S3. Grain weight per spike / spike weight, awns weight per spike / spike weight, chaff weight per spike / spike weight as affected by the treatments (experimental year 2021-2022). Mean: mean values; SD: standard deviation, Diff: the difference between the mean value of the treatment and that of the control, SC: significance code; ns: not statistically significant, * <0.05, **<0.01, ***<0.001.

Treatment	Grain weight per spike / spike weight					Awns weight per spike / spike weight					Chaff weight per spike / spike weight				
	mean	SD	Diff	p-value	SC	mean	SD	Diff	p-value	SC	mean	SD	Diff	p-value	SC
Control	0.809	0.066				0.084	0.018				0.107	0.061			
SW7	0.702	0.027	-0.11	0.0016	**	0.074	0.013	-0.01	1.0000	ns	0.224	0.032	0.117	0.0004	***
Cys	0.803	0.093	-0.01	1.0000	ns	0.083	0.010	-0.001	1.0000	ns	0.114	0.091	0.007	1.0000	ns
Cys x SW7	0.780	0.089	-0.03	1.0000	ns	0.077	0.008	-0.007	1.0000	ns	0.144	0.088	0.036	0.9999	ns
Met	0.832	0.052	0.023	1.0000	ns	0.062	0.009	-0.021	0.8790	ns	0.106	0.054	-0.002	1.0000	ns
Met x SW7	0.772	0.051	-0.04	0.9998	ns	0.079	0.014	-0.005	1.0000	ns	0.149	0.058	0.042	0.9988	ns
ZnSO4	0.824	0.098	0.015	1.0000	ns	0.039	0.014	-0.045	0.0002	***	0.137	0.098	0.029	1.0000	ns
ZnSO4 x SW7	0.776	0.082	-0.03	1.0000	ns	0.063	0.017	-0.021	0.9110	ns	0.161	0.092	0.054	0.9368	ns
ZnSO4 x Cys	0.804	0.077	-0.01	1.0000	ns	0.063	0.013	-0.02	0.9280	ns	0.133	0.082	0.025	1.0000	ns
ZnSO4 x Cys x SW7	0.729	0.045	-0.08	0.1413	ns	0.064	0.007	-0.02	0.9480	ns	0.207	0.045	0.1	0.0103	*
ZnSO4 x Met	0.614	0.140	-0.2	0.0000	***	0.061	0.016	-0.023	0.7759	ns	0.325	0.151	0.218	0.0000	***
ZnSO4 x Met x SW7	0.752	0.091	-0.06	0.8462	ns	0.059	0.007	-0.025	0.6019	ns	0.189	0.092	0.082	0.1489	ns
FeSO4	0.607	0.081	-0.2	0.0000	***	0.054	0.011	-0.03	0.1700	ns	0.339	0.087	0.232	0.0000	***
FeSO4 x SW7	0.656	0.065	-0.15	0.0000	***	0.076	0.030	-0.008	1.0000	ns	0.268	0.080	0.161	0.0000	***
FeSO4 x Cys	0.717	0.063	-0.09	0.0236	*	0.073	0.019	-0.01	1.0000	ns	0.210	0.055	0.103	0.0059	**
FeSO4 x Cys x SW7	0.648	0.092	-0.16	0.0000	***	0.081	0.037	-0.003	1.0000	ns	0.271	0.105	0.163	0.0000	***
FeSO4 x Met	0.671	0.053	-0.14	0.0000	***	0.061	0.023	-0.022	0.8109	ns	0.267	0.060	0.16	0.0000	***
FeSO4 x Met x SW7	0.789	0.054	-0.02	1.0000	ns	0.062	0.014	-0.022	0.8144	ns	0.150	0.052	0.043	0.9983	ns
MnSO4	0.815	0.090	0.006	1.0000	ns	0.087	0.070	0.003	1.0000	ns	0.098	0.033	-0.01	1.0000	ns
MnSO4 x SW7	0.728	0.087	-0.08	0.1290	ns	0.095	0.043	0.011	1.0000	ns	0.177	0.104	0.069	0.4903	ns
MnSO4 x Cys	0.744	0.037	-0.07	0.5940	ns	0.075	0.025	-0.008	1.0000	ns	0.181	0.047	0.073	0.3618	ns
MnSO4 x Cys x SW7	0.686	0.104	-0.12	0.0001	***	0.079	0.038	-0.004	1.0000	ns	0.234	0.099	0.127	0.0000	***
MnSO4 x Met	0.697	0.110	-0.11	0.0006	***	0.078	0.014	-0.006	1.0000	ns	0.225	0.116	0.117	0.0004	***
MnSO4 x Met x SW7	0.775	0.047	-0.03	1.0000	ns	0.085	0.017	0.001	1.0000	ns	0.141	0.055	0.033	1.0000	ns
CuSO4	0.799	0.089	-0.01	1.0000	ns	0.093	0.033	0.009	1.0000	ns	0.109	0.092	0.001	1.0000	ns
CuSO4 x SW7	0.538	0.062	-0.27	0.0000	***	0.125	0.058	0.041	0.0010		0.336	0.079	0.229	0.0000	***
CuSO4 x Cys	0.692	0.110	-0.12	0.0002	***	0.069	0.034	-0.015	0.9996	ns	0.239	0.092	0.132	0.0000	***
CuSO4 x Cys x SW7	0.721	0.116	-0.09	0.0464	*	0.076	0.021	-0.008	1.0000	ns	0.203	0.126	0.096	0.0198	*
CuSO4 x Met	0.590	0.101	-0.22	0.0000	***	0.076	0.028	-0.007	1.0000	ns	0.333	0.119	0.226	0.0000	***
CuSO4 x Met x SW7	0.667	0.052	-0.14	0.0000	***	0.081	0.036	-0.003	1.0000	ns	0.252	0.069	0.145	0.0000	***
FABo	0.809	0.155	0	1.0000	ns	0.088	0.045	0.005	1.0000	ns	0.102	0.160	-0.005	1.0000	ns
FABo x SW7	0.688	0.131	-0.12	0.0001	***	0.095	0.086	0.011	1.0000	ns	0.217	0.121	0.109	0.0018	**
FABo x Cys	0.790	0.070	-0.02	1.0000	ns	0.094	0.041	0.011	1.0000	ns	0.115	0.069	0.008	1.0000	ns
FABo x Cys x SW7	0.780	0.115	-0.03	1.0000	ns	0.087	0.025	0.003	1.0000	ns	0.133	0.099	0.026	1.0000	ns
FABo x Met	0.553	0.103	-0.26	0.0000	***	0.087	0.028	0.004	1.0000	ns	0.360	0.113	0.252	0.0000	***
FABo x Met x SW7	0.761	0.100	-0.05	0.9810	ns	0.096	0.069	0.013	1.0000	ns	0.143	0.096	0.035	1.0000	ns

Table S4. Spike length / spike weight, spikelet number per spike / spike weight, grain number per spike / spike weight as affected by the treatments (experimental year 2021-2022). Mean: mean values; SD: standard deviation, Diff: the difference between the mean value of the treatment and that of the control, SC: significance code; ns: not statistically significant, * <0.05, **<0.01, ***<0.001.

Treatment	Spike length / spike weight					Spikelet number per spike / spike weight					Grain number per spike / spike weight				
	mean	SD	Diff	p-value	SC	mean	SD	Diff	p-value	SC	mean	SD	Diff	p-value	SC
Control	2.88	0.64				8.0	2.0				15.59	3.02			
SW7	2.20	0.30	-0.68	0.8961	ns	6.1	0.8	-1.88	0.9600	ns	11.30	3.01	-4.286	0.7895	ns
Cys	2.60	0.54	-0.28	1.0000	ns	7.2	1.4	-0.804	1.0000	ns	13.50	2.92	-2.086	1.0000	ns
Cys x SW7	2.90	0.38	0.014	1.0000	ns	7.0	1.5	-0.994	1.0000	ns	14.63	2.32	-0.962	1.0000	ns
Met	3.00	0.79	0.122	1.0000	ns	8.6	2.7	0.625	1.0000	ns	13.69	3.45	-1.9	1.0000	ns
Met x SW7	2.85	0.56	-0.03	1.0000	ns	7.0	2.3	-1.028	1.0000	ns	15.69	4.15	0.101	1.0000	ns
ZnSO4	3.36	0.65	0.48	0.9996	ns	8.6	2.5	0.608	1.0000	ns	15.55	3.79	-0.043	1.0000	ns
ZnSO4 x SW7	3.85	1.90	0.971	0.1823	ns	9.8	5.1	1.79	0.9796	ns	17.73	10.44	2.143	1.0000	ns
ZnSO4 x Cys	3.63	1.81	0.752	0.7532	ns	10.3	5.2	2.254	0.7418	ns	20.53	12.99	4.937	0.4719	ns
ZnSO4 x Cys x SW7	2.41	0.30	-0.47	0.9997	ns	7.1	1.3	-0.874	1.0000	ns	16.28	2.72	0.686	1.0000	ns
ZnSO4 x Met	3.35	1.71	0.467	0.9998	ns	8.6	4.5	0.615	1.0000	ns	17.90	10.16	2.306	1.0000	ns
ZnSO4 x Met x SW7	2.53	0.56	-0.35	1.0000	ns	6.3	1.8	-1.668	0.9931	ns	12.72	3.61	-2.874	0.9992	ns
FeSO4	2.43	0.64	-0.45	0.9999	ns	6.7	2.3	-1.325	0.9999	ns	11.46	2.64	-4.126	0.8501	ns
FeSO4 x SW7	2.83	0.80	-0.05	1.0000	ns	8.2	2.8	0.185	1.0000	ns	13.47	4.92	-2.123	1.0000	ns
FeSO4 x Cys	2.98	0.76	0.102	1.0000	ns	8.4	2.9	0.351	1.0000	ns	16.42	5.08	0.835	1.0000	ns
FeSO4 x Cys x SW7	3.29	1.25	0.407	1.0000	ns	7.8	3.3	-0.236	1.0000	ns	19.56	8.89	3.973	0.8975	ns
FeSO4 x Met	2.26	0.61	-0.62	0.9655	ns	6.0	1.9	-1.999	0.9165	ns	11.90	4.79	-3.693	0.9562	ns
FeSO4 x Met x SW7	2.47	0.54	-0.41	1.0000	ns	7.1	2.2	-0.875	1.0000	ns	15.02	3.64	-0.571	1.0000	ns
MnSO4	3.53	1.32	0.649	0.9423	ns	8.8	4.1	0.793	1.0000	ns	22.36	9.50	6.767	0.0196	*
MnSO4 x SW7	3.74	1.47	0.854	0.4591	ns	9.6	3.7	1.592	0.9968	ns	20.12	8.01	4.53	0.6786	ns
MnSO4 x Cys	2.43	0.83	-0.45	0.9999	ns	6.7	2.9	-1.271	1.0000	ns	14.52	6.14	-1.073	1.0000	ns
MnSO4 x Cys x SW7	3.33	0.89	0.445	0.9999	ns	9.1	2.8	1.121	1.0000	ns	15.38	5.75	-0.207	1.0000	ns
MnSO4 x Met	2.68	0.59	-0.2	1.0000	ns	7.1	2.0	-0.902	1.0000	ns	15.45	5.40	-0.137	1.0000	ns
MnSO4 x Met x SW7	2.81	0.75	-0.07	1.0000	ns	8.5	3.4	0.518	1.0000	ns	18.09	5.90	2.5	1.0000	ns
CuSO4	3.70	0.77	0.817	0.5688	ns	9.2	2.5	1.215	1.0000	ns	20.57	4.79	4.985	0.4481	ns
CuSO4 x SW7	2.73	0.95	-0.15	1.0000	ns	8.0	3.9	0.019	1.0000	ns	14.45	6.02	-1.136	1.0000	ns
CuSO4 x Cys	3.13	0.73	0.25	1.0000	ns	8.6	2.7	0.627	1.0000	ns	12.98	3.31	-2.608	0.9999	ns
CuSO4 x Cys x SW7	3.15	1.00	0.265	1.0000	ns	8.8	3.1	0.757	1.0000	ns	17.79	5.90	2.198	1.0000	ns
CuSO4 x Met	2.86	1.03	-0.02	1.0000	ns	7.8	3.6	-0.237	1.0000	ns	13.51	4.87	-2.075	1.0000	ns
CuSO4 x Met x SW7	2.69	1.52	-0.19	1.0000	ns	8.1	4.3	0.091	1.0000	ns	12.41	5.15	-3.177	0.9953	ns
FABo	4.13	1.93	1.253	0.0055	**	9.6	5.3	1.62	0.9957	ns	21.09	9.50	5.502	0.2275	ns
FABo x SW7	3.35	1.84	0.468	0.9998	ns	9.1	5.5	1.04	1.0000	ns	19.01	10.49	3.421	0.9847	ns
FABo x Cys	3.60	1.17	0.716	0.8374	ns	9.2	3.1	1.198	1.0000	ns	18.82	5.25	3.231	0.9938	ns
FABo x Cys x SW7	3.73	1.42	0.845	0.4832	ns	10.3	4.1	2.312	0.6894	ns	16.17	6.72	0.581	1.0000	ns
FABo x Met	3.02	1.41	0.14	1.0000	ns	8.9	3.3	0.847	1.0000	ns	16.32	5.97	0.733	1.0000	ns
FABo x Met x SW7	3.28	1.36	0.396	1.0000	ns	8.0	3.5	0.021	1.0000	ns	22.20	8.04	6.61	0.0280	*

Table S5. Pearson's Correlation Test providing Pearson's linear correlation coefficient, along with the estimated confidence interval, and the p-value for the hypothesis that the coefficient is a statistical ratio of zero. LB: lower bound, UB: upper bound.

Trait 1	Trait 2	Coefficient	p-value	LB	UB
AWS	GNS	0.230	1.870×10^{-01}	–0.112	0.516
AWS	GWS	0.220	1.930×10^{-01}	–0.115	0.513
AWS	SL	-0.021	9.040×10^{-01}	–0.347	0.310
AWS	SlNS	0.130	4.580×10^{-01}	–0.210	0.438
AWS	SW	0.410	1.260×10^{-02}	0.096	0.652
GNS	GWS	0.500	1.810×10^{-03}	0.208	0.713
GNS	SL	0.560	3.980×10^{-04}	0.282	0.750
GNS	SlNS	0.310	7.030×10^{-02}	–0.026	0.576
GNS	SW	0.280	1.040×10^{-01}	–0.058	0.554
GWS	SL	0.510	1.480×10^{-03}	0.218	0.718
GWS	SlNS	0.500	1.720×10^{-03}	0.210	0.714
GWS	SW	0.740	2.910×10^{-07}	0.539	0.858
SL	SlNS	0.590	1.500×10^{-04}	0.325	0.770
SL	SW	0.290	8.480×10^{-02}	–0.041	0.566
SlNS	SW	0.440	6.630×10^{-03}	0.136	0.674

Table S6. The percentage change in each trait's mean value relative to the reference treatment (control; Δ%), along with the statistical significance (experimental year 2021-2022). SC: significance code, SL: spike length, SlNS: spikelet number per spike, GNS: grain number per spike, SW: spike weight, AWS: awns weight per spike, CWS: chaff weight per spike, GWS: grain weight per spike, GW: grain weight. A scale has been adobted to visualize the tendencies for increasing or decreasing, when Δ% is not statistically significant. ns: not statistically significant, *: p-value < 0.05, **: p-value < 0.01, ***: p-value < 0.001. The p-values are provided in the corresponding Tables.

Treatment	SL		SlNS		GNS		SW		GWS		AWS		CWS		GW	
	Δ%	SC	Δ%	SC	Δ%	SC	Δ%	SC	Δ%	SC	Δ%	SC	Δ%	SC	Δ%	SC
SW7	-6	ns	-5	ns	-12	ns	19	ns	3	ns	7	ns	159	***	24	ns
Cys	-16	***	-15	ns	-20	***	-8	ns	-10	ns	-7	ns	8	ns	15	ns
Cys x SW7	2	ns	-12	ns	-7	ns	-1	ns	-6	ns	-7	ns	38	ns	2	ns
Met	-14	***	-10	ns	-28	***	-16	ns	-14	ns	-36	***	-11	ns	19	ns
Met x SW7	4	ns	-10	ns	3	ns	4	ns	-2	ns	-1	ns	52	ns	-4	ns
ZnSO4	0	ns	-8	ns	-17	*	-15	ns	-16	ns	-60	***	22	ns	4	ns
ZnSO4 x SW7	-1	ns	-10	ns	-19	**	-16	ns	-22	ns	-39	***	46	ns	0	ns
ZnSO4 x Cys	-9	ns	-6	ns	-9	ns	-22	ns	-24	*	-42	***	4	ns	-11	ns
ZnSO4 x Cys x SW7	-3	ns	3	ns	19	**	14	ns	2	ns	-11	ns	128	***	-15	ns
ZnSO4 x Met	1	ns	-7	ns	-3	ns	-2	ns	-29	***	-31	***	235	***	-24	ns
ZnSO4 x Met x SW7	6	ns	-5	ns	-3	ns	21	ns	12	ns	-13	ns	119	**	20	ns
FeSO4	-10	*	-11	ns	-22	***	9	ns	-20	ns	-29	***	264	***	4	ns
FeSO4 x SW7	-16	***	-12	ns	-27	***	-12	ns	-30	***	-23	*	137	***	0	ns
FeSO4 x Cys	-10	*	-9	ns	-9	ns	-12	ns	-22	ns	-23	ns	79	ns	-13	ns
FeSO4 x Cys x SW7	-10	*	-24	***	-4	ns	-14	ns	-32	***	-25	*	136	***	-28	ns
FeSO4 x Met	-18	***	-21	**	-23	***	5	ns	-14	ns	-26	*	174	***	19	ns
FeSO4 x Met x SW7	-5	ns	-1	ns	6	ns	11	ns	8	ns	-19	ns	62	ns	2	ns
MnSO4	2	ns	-10	ns	16	*	-10	ns	-8	ns	-21	ns	-21	ns	-19	ns
MnSO4 x SW7	3	ns	-3	ns	3	ns	-13	ns	-21	ns	-9	ns	50	ns	-22	ns
MnSO4 x Cys	-8	ns	-9	ns	-2	ns	12	ns	2	ns	-2	ns	103	*	7	ns
MnSO4 x Cys x SW7	-5	ns	-5	ns	-20	***	-14	ns	-28	**	-18	ns	93	ns	-4	ns
MnSO4 x Met	-8	ns	-11	ns	-4	ns	-1	ns	-16	ns	-6	ns	118	**	-9	ns
MnSO4 x Met x SW7	-17	***	-10	ns	-3	ns	-14	ns	-18	ns	-11	ns	14	ns	-13	ns
CuSO4	0	ns	-10	ns	1	ns	-22	ns	-23	*	-15	ns	-22	ns	-24	ns
CuSO4 x SW7	-16	***	-11	ns	-19	***	-3	ns	-35	***	31	***	219	***	-19	ns
CuSO4 x Cys	-7	ns	-8	ns	-29	***	-14	ns	-27	**	-27	**	103	*	4	ns
CuSO4 x Cys x SW7	-6	ns	-5	ns	-3	ns	-9	ns	-18	ns	-17	ns	67	ns	-15	ns
CuSO4 x Met	-7	ns	-10	ns	-19	***	-2	ns	-30	***	-14	ns	235	***	-13	ns
CuSO4 x Met x SW7	-19	***	-11	ns	-29	***	-3	ns	-21	ns	-12	ns	147	***	13	ns
FABo	6	ns	-12	ns	-1	ns	-15	ns	-17	ns	-16	ns	1	ns	-17	ns
FABo x SW7	-8	ns	-10	ns	-3	ns	-7	ns	-19	ns	-13	ns	93	ns	-19	ns
FABo x Cys	-17	***	-22	***	-19	***	-31	***	-33	***	-25	*	-22	ns	-17	ns
FABo x Cys x SW7	-7	ns	-6	ns	-26	***	-23	ns	-24	*	-21	ns	-14	ns	7	ns
FABo x Met	-24	***	-18	*	-25	***	-23	ns	-48	***	-20	ns	175	***	-30	ns
FABo x Met x SW7	-20	***	-29	***	0	ns	-25	*	-28	***	-18	ns	-4	ns	-28	ns

D+	50%		-
C+	30%	to	49%
B+	15%	to	29%
A+	0	to	14%
A-	-1%	to	-14%
B-	-15%	to	-29%
C-	-30%	to	-49%
D-	-40%		-

Table S7. The percentage change in each trait's mean value (MV) relative to the reference treatment (control; Δ%), along with the statistical significance (experimental year 2021-2022). SC: significance code GWS/SW: grain weight per spike/spike weight, AWS/SW: awns weight per spike/spike weight, CWS/SW: chaff weight per spike/spike weight, SlNS/SW: spikelet number per spike/spike weight, GNS/SW: grain number per spike/spike weight, SL/SW: spike length/spike weight/spike weight. A scale has been adobted to visualize the tendencies for increasing or decreasing, when Δ% is not statistically significant. ns: not statistically significant, *: p-value < 0.05, **: p-value < 0.01, ***: p-value < 0.001. The p-values are provided in the corresponding Tables.

Treatment	GWS / SW		AWS / SW		CWS / SW		SlS / SW		GNS / SW		SL / SW	
	Δ%	SC	Δ%	SC	Δ%	SC	Δ%	SC	Δ%	SC	Δ%	SC
SW7	-13	**	-12	ns	**109**	***	-23	ns	-27	ns	-24	ns
Cys	-1	ns	-1	ns	7	ns	-10	ns	-13	ns	-10	ns
Cys x SW7	-4	ns	-8	ns	35	ns	-12	ns	-6	ns	0	ns
Met	3	ns	-26	ns	-1	ns	8	ns	-12	ns	4	ns
Met x SW7	-5	ns	-6	ns	39	ns	-13	ns	1	ns	-1	ns
ZnSO4	2	ns	-54	***	28	ns	8	ns	0	ns	17	ns
ZnSO4 x SW7	-4	ns	-25	ns	50	ns	22	ns	14	ns	34	ns
ZnSO4 x Cys	-1	ns	-25	ns	24	ns	28	ns	32	ns	26	ns
ZnSO4 x Cys x SW7	-10	ns	-24	ns	**93**	*	-11	ns	4	ns	-16	ns
ZnSO4 x Met	**-24**	***	-27	ns	**204**	***	8	ns	15	ns	16	ns
ZnSO4 x Met x SW7	**-7**	ns	-30	ns	77	ns	-21	ns	-18	ns	-12	ns
FeSO4	**-25**	***	-36	ns	**217**	***	-17	ns	-26	ns	-16	ns
FeSO4 x SW7	**-19**	***	-10	ns	**150**	***	2	ns	-14	ns	-2	ns
FeSO4 x Cys	**-11**	*	-13	ns	**96**	**	4	ns	5	ns	4	ns
FeSO4 x Cys x SW7	**-20**	***	-4	ns	**153**	***	-3	ns	25	ns	14	ns
FeSO4 x Met	**-17**	***	-27	ns	**150**	***	-25	ns	-24	ns	-22	ns
FeSO4 x Met x SW7	-2	ns	-26	ns	40	ns	-11	ns	-4	ns	-14	ns
MnSO4	1	ns	4	ns	-8	ns	10	ns	43	ns	23	ns
MnSO4 x SW7	-10	ns	13	ns	65	ns	20	ns	29	ns	30	ns
MnSO4 x Cys	-8	ns	-11	ns	69	ns	-16	ns	-7	ns	-16	ns
MnSO4 x Cys x SW7	**-15**	***	-6	ns	**119**	***	14	ns	-1	ns	15	ns
MnSO4 x Met	-14	***	-7	ns	**110**	***	-11	ns	-1	ns	-7	ns
MnSO4 x Met x SW7	-4	ns	1	ns	32	ns	6	ns	16	ns	-2	ns
CuSO4	-1	ns	11	ns	2	ns	15	ns	32	ns	28	ns
CuSO4 x SW7	**-33**	***	49	*	**214**	***	0	ns	-7	ns	-5	ns
CuSO4 x Cys	**-14**	***	-18	ns	**123**	***	8	ns	-17	ns	9	ns
CuSO4 x Cys x SW7	-11	*	-10	ns	**90**	*	9	ns	14	ns	9	ns
CuSO4 x Met	**-27**	***	-10	ns	**211**	***	-3	ns	-13	ns	-1	ns
CuSO4 x Met x SW7	**-18**	***	-4	ns	**136**	***	1	ns	-20	ns	-7	ns
FABo	0	ns	5	ns	-5	ns	20	ns	35	ns	43	**
FABo x SW7	**-15**	***	13	ns	**103**	**	13	ns	22	ns	16	ns
FABo x Cys	-2	ns	12	ns	7	ns	15	ns	21	ns	25	ns
FABo x Cys x SW7	-4	ns	4	ns	24	ns	29	ns	4	ns	29	ns
FABo x Met	**-32**	***	4	ns	**236**	***	11	ns	5	ns	5	ns
FABo x Met x SW7	-6	ns	14	ns	34	ns	0	ns	42	*	14	ns

D+	50%	-		
C+	30%	to	49%	
B+	15%	to	29%	
**A+**	0	to	14%	
A-	-1%	to	-14%	
B-	-15%	to	-29%	
C-	-30%	to	-49%	
D-	-40%	-		

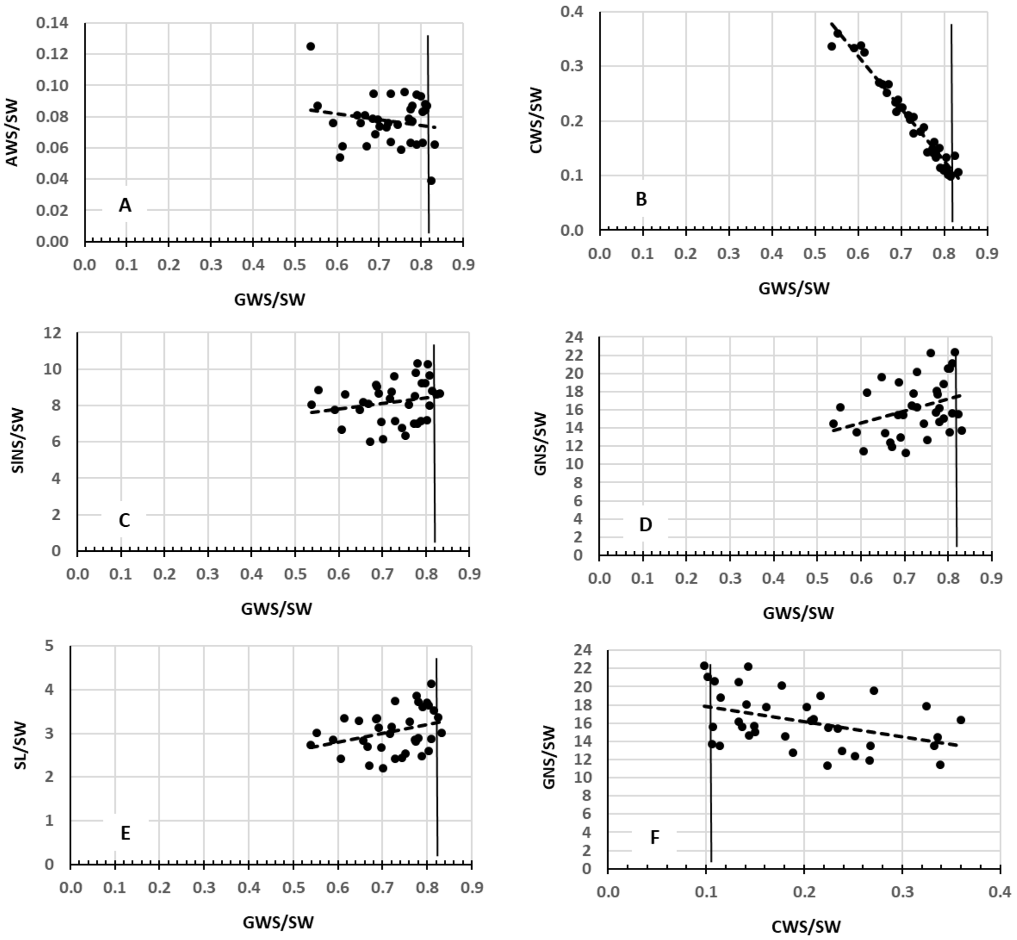

Figure S15. Relationships between GWS/SW and AWS/SW (A) , WS/SW (B), SlNS/SW (C), GNS/SW (D), and SL/SW (E), along with CWS/SW vs. GNS/SW (F). Line indicates the corresponding value of control treatment.
